# Exploiting the DNA Damaging Activity of Liposomal Low Dose Cytarabine for Cancer Immunotherapy

**DOI:** 10.3390/pharmaceutics14122710

**Published:** 2022-12-03

**Authors:** Jordan D. Lewicky, Alexandrine L. Martel, Nya L. Fraleigh, Emilie Picard, Leila Mousavifar, Arnaldo Nakamura, Francisco Diaz-Mitoma, René Roy, Hoang-Thanh Le

**Affiliations:** 1 Health Sciences North Research Institute, 56 Walford Road, Sudbury, ON P3E 2H2, Canada; 2 Cancer Research Center of Lyon, 28 rue Laennec, 69008 Lyon, France; 3 Glycosciences and Nanomaterial Laboratory, Université du Québec à Montréal, P.O. Box 8888, Succ. Centre-Ville, Montréal, QC H3C 3P8, Canada; 4 Armand-Frappier Santé Biotechnologie Research Centre, Institut National de la Recherche Scientifique, 531 Boulevard des Prairies, Laval, QC H7V 1B7, Canada; 5 Medicinal Sciences Division, NOSM University, 935 Ramsey Lake Road, Sudbury, ON P3E 2C6, Canada

**Keywords:** cancer immunotherapy, immunomodulation, cGAS-STING, DNA damage response, low dose cytarabine, mannosylated cationic liposome, delivery system

## Abstract

Perhaps the greatest limitation for the continually advancing developments in cancer immunotherapy remains the immunosuppressive tumor microenvironment (TME). The cyclic GMP-AMP synthase (cGAS)-stimulator of interferon genes (STING) axis is an emerging immunotherapy target, with the resulting type I interferons and transcription factors acting at several levels in both tumor and immune cells for the generation of adaptive T cell responses. The cGAS-STING axis activation by therapeutic agents that induce DNA damage, such as certain chemotherapies, continues to be reported, highlighting the importance of the interplay of this signaling pathway and the DNA damage response in cancer immunity/immunotherapy. We have developed a multi-targeted mannosylated cationic liposomal immunomodulatory system (DS) which contains low doses of the chemotherapeutic cytarabine (Ara-C). In this work, we show that entrapment of non-cytotoxic doses of Ara-C within the DS improves its ability to induce DNA double strand breaks in human ovarian and colorectal cancer cell lines, as well as in various immune cells. Importantly, for the first time we demonstrate that the DNA damage induced by Ara-C/DS translates into cGAS-STING axis activation. We further demonstrate that Ara-C/DS-mediated DNA damage leads to upregulation of surface expression of immune ligands on cancer cells, coinciding with priming of cytotoxic lymphocytes as assessed using an ex vivo model of peripheral blood mononuclear cells from colorectal cancer patients, as well as an in vitro NK cell model. Overall, the results highlight a broad immunotherapeutic potential for Ara-C/DS by enhancing tumor-directed inflammatory responses.

## 1. Introduction

In testament to the power of the immune system for the selective elimination of cancerous cells, the prevalence of cancer immunotherapy continues to increase [1]. Perhaps the most successful of the several immunotherapeutic approaches being developed are checkpoint inhibitors that modulate the aberrant immune regulation acting as immunosuppressive “brakes” hindering anti-cancer immune responses [2]. Despite applicability in various types of cancers, the benefits of checkpoint inhibitors have been limited to specific cohorts of patients [3], with accumulating evidence suggesting that patient responses are highly dependent on inherent tumor immunogenicity [4]. A major limiting factor for tumor immunogenicity is the surrounding tumor microenvironment (TME), where large populations of immunosuppressive cells, including tumor associated macrophages and regulatory T cells, contribute to a non-inflamed “cold” TME that inhibits the infiltration and activity of T cells within the tumor [5,6]. Strategies that can induce an inflamed “hot” TME and activate cytotoxic T lymphocytes (CTL) for cancer cell immunosurveillance are currently being sought [7].

**Figure 1:**
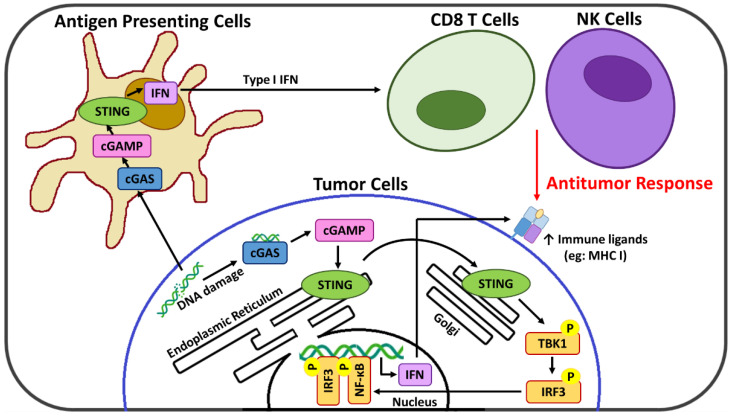
Summary of the cGAS-STING pathway and its role in cancer immunosurveillance.

Emerging evidence indicates that the cyclic GMP-AMP synthase (cGAS)-stimulator of interferon genes (STING) axis is a major innate immune pathway involved in generating spontaneous antitumor T cell responses [8,9] and thus a promising cancer immunotherapy target [10]. cGAS recognizes and responds to the presence of cytosolic DNA, where it catalyzes the formation of the cyclic GMP-AMP (cGAMP) dinucleotide second messenger [11] that activates STING at the endoplasmic reticulum and initiates its relocation to the cytoplasm [12]. Activated STING serves as a signaling platform to recruit TANK binding kinase-1 (TBK1) which after autophosphorylation catalyzes the phosphorylation of both the STING protein and interferon regulatory factor 3 (IRF3) [13]. This activated IRF3 translocates to the nucleus where it drives transcription of a subset of immune-stimulated genes, type 1 interferons (IFNs), as well as other transcription factors such as NF-κB that potentiate the expression of additional pro-inflammatory genes [14]. cGAS-STING signaling acts at several levels in both tumor and immune cells for the generation of adaptive T cell responses. In cancer cells, the cGAS-STING signaling axis is linked with remodeling of the immunosuppressive TME [15,16], and, via the resulting type 1 IFNs, upregulation of suppressed major histocompatibility complex (MHC) class I expression necessary for CTL recognition and targeting [17]. In immune cells, cGAS-STING activation links innate and adaptive immunity, ultimately promoting the infiltration and priming of CTL within the TME [16,18]. In addition, new evidence suggests that cGAS-STING activation may also promote TME infiltration of natural killer (NK) cells [19,20], which also play a critical role in immune activation against abnormal cells and are an emerging target of immunotherapy strategies [21,22]. Importantly, the cGAS-STING pathway also leads to cross-talk between tumor and immune cells, with antigen-presenting cells (APC) sensing tumor-derived cGAMP or damaged DNA that further enhances the STING-dependent antitumor immunity [10]. A summary of the cGAS-STING pathway is presented in Figure 1.

The promising immunotherapeutic potential of cGAS-STING activation has fueled the development of various classes of STING agonists [23]. Structural analogues of cGAMP have been shown to induce robust antitumor immunity but suffer from pharmacokinetic limitations and off-target toxicity that necessitate direct intratumoral injection [24]. Several non-cyclic dinucleotide agonists of diverse structure have been reported with the potential for systemic administration, however their selectivity and utility have yet to be confirmed [23]. Recently, the importance of the DNA damage response (DDR), and its interplay with the cGAS-STING axis in cancer immunity/immunotherapy is attracting attention [25,26]. The ability of therapeutic agents that induce DNA damage, such as certain chemotherapies and inhibitors of DDR components, to activate STING signaling continues to be demonstrated, where the fragmented DNA that results ultimately enters the cytoplasm and is recognized by cGAS [26,27,28].

The immunostimulatory activity of certain chemotherapeutics at low doses continues to be investigated for immunotherapeutic applications, with various effects on different immune cells including CTL [29,30], and those that modulate the TME [31,32,33,34]. We recently reported the immunomodulatory properties of low doses of the pyrimidine nucleoside analogue chemotherapeutic cytarabine (Ara-C) entrapped inside a novel multi-targeted mannosylated cationic liposome delivery system (Ara-C/DS) [35]. Ara-C is commonly used for treatment of acute myeloid leukemia (AML), but is known to suffer from low biological stability and cellular uptake that has ultimately limited its clinical use [36]. Ara-C is easily entrapped within the DS (>90% entrapment efficacy) which greatly improves its stability in human plasma [35]. The DS has been designed [35,37,38,39] to exploit and selectively target the overexpression of the mannose receptor in both cancer and immune cells [40,41], with the cationic particle charge and entrapped muramyl dipeptide (MDP) included for their known ability to promote positive antitumor immunological effects [42,43,44,45]. Ara-C/DS increased CD80 and decreased CD163 expression on immunosuppressive macrophages, and in addition it increased IFN-γ and IL-12p40 expression in peripheral blood mononuclear cells (PBMC) with no changes in IL-10 levels [35]. Increased MHC class I expression on breast, lung, and blood cancer cells was observed in response to Ara-C/DS treatment [35]. Ara-C/DS also led to the production of influenza-specific IFN-γ^+^ CD8 T cells in viral peptide-challenged lymphocytes from both humans and vaccinated mice [35]. This data suggest that Ara-C can be repurposed for immunotherapy applications when combined with DS, which serves to stabilize, deliver, and enhance Ara-C’s activity.

The chemotherapeutic activity of Ara-C comes from its ability to block cell proliferation by incorporating into the DNA, where it inhibits the replicative machinery and ultimately produces double strand breaks (DSB) [36]. Based on this mechanism and our previously published results [35], the Ara-C/DS system was investigated for its ability to activate the cGAS-STING axis. In this work, we confirm that entrapment within the DS improves the ability of Ara-C to induce DNA damage in human ovarian carcinoma cell lines (A2780 & its cisplatin-resistant counterpart A2780R), and colorectal carcinoma cell lines (HT-29 & HCT 116), as measured by the phosphorylation of histone H2AX, a marker of DNA DSB [46]. For the first time, we demonstrate that the DNA damage induced by Ara-C/DS translates into activation of the cGAS-STING axis, as evidenced by the phosphorylation of STING (P-STING), IRF3 (P-IRF3) and TBK1 (P-TBK1). In addition, we show that Ara-C/DS has similar activity in immune cells as represented by both THP-1 derived human macrophages and human PBMC. To confirm the downstream effects of the DDR and STING activation, we further demonstrate that Ara-C/DS is able to increase the surface expression on the cancer cell lines of the T cell ligand MHC I, as well as the NK cell ligands MHC class I chain-related protein A and B [MICA/B], and UL16-binding proteins [ULBP] 2, 5 and 6). Finally, Ara-C/DS increases cytotoxic T cell responses in PBMC isolated from colorectal cancer patients in the presence of tumor-associated antigens (TAA), and improves the cytotoxic activity of the NK cell line NK-92 in a co-culture model with HCT 116 cells. Overall, the results further highlight the broad immunotherapeutic potential of Ara-C/DS for the treatment of cancer.

## 2. Materials and Methods

### 2.1. Liposomal Formulation

The liposomal DS was comprised of multiple components, including an aromatic mannose lipid, dimethyldioctadecyl-ammonium bromide (DDAB) cationic lipid (Sigma-Aldrich, St Louis, MO, USA), cholesterol (Sigma-Aldrich, St Louis, MO, USA), and MDP (Sigma-Aldrich, St Louis, MO, USA). The aromatic mannose lipid was synthesized according to published procedures [39] and stored at −20 °C as solid powder. Cationic liposomal stocks were prepared using a heating and tert-butanol (TBA, Alfa Aesar, Ottawa, ON, Canada) method from published methods [47] by dissolving the aromatic mannose lipid, cholesterol, and DDAB (5:4:1 molar ratio, based on the optimal conditions for entrapment efficacy and stability of the formulations) in TBA at a concentration of 50 mg/mL for storage at 4 °C. Stock solutions of MDP (5 mg/mL) or Ara-C (5 mM) (both from Sigma-Aldrich, St Louis, MO, USA) were prepared in sterile ddH_2_O, aliquoted, and stored at −20 °C. All Ara-C/DS and DS formulations were prepared fresh according to our optimized methods.

For all in vitro assays, the formulations were prepared by combining 0.5 µL of 5 mM Ara-C stock (final concentration: 25 µM), 1 µL of 5 mg/mL MDP stock (final concentration: 50 µg/mL), and 1 µL of 50 mg/mL cationic liposome stock (final concentration: 0.5 mg/mL) and adding ddH_2_O to a final volume of 100 µL. The formulations were further diluted in ddH_2_O or media as needed for experimental conditions. Concentrations of DS for assays are reported as the amount of cationic liposome, in which MDP is entrapped at a 1:10 ratio.

### 2.2. Transmission Electron Microscopy (TEM)

The cationic liposome formulation was prepared as above to a final concentration of 0.5 mg/mL in aqueous solution, and was stored at room temperature for 24 h or two weeks. To prepare the TEM grid, 50 µL of the cationic liposome formulation was added to parafilm, and grids were immersed for 20 min prior to drying using bibulous paper. Grids were further immersed in a drop of a 3% phosphotungstic acid solution (PTA, pH = 6.0) for 1 min before drying. Imaging was performed at 75 kV on a Hitachi-7100 transmission electron microscope (Hitachi Limited, Hitachi, Japan) equipped with an XR-100 camera (Advanced Microscopy Techniques Corporation, Danvers, MA, USA).

### 2.3. Cell Lines

HCT 116, HT-29, THP-1, and NK-92 were provided by Dr. Hoyun Lee (HSNRI, Sudbury, ON, Canada). A2780 (parental) and A2780R (cisplatin resistant) were provided by Dr. Barbara Vanderhyden (Ottawa Hospital Research Institute, OHRI, Ottawa, ON, Canada). HCT 116, HT-29, A2780, A2780R and THP-1 cells were grown in RPMI-1640 (HyClone, Logan, UT, USA) supplemented with 10% heat-inactivated (HI) FBS (Gibco, Grand Island, NY, USA) and 100 units/mL streptomycin/penicillin (HyClone, Logan, UT, USA). When required, adherent cells were collected using 0.25% Trypsin-EDTA (Gibco, Grand Island, NY, USA). NK-92 cells were cultured in MEM Alpha medium (Corning, Manassas, VA, USA) with 12.5% horse serum (Gibco, Carlsbad, CA, USA), 12.5% HI FBS, 0.2 mM inositol (Sigma-Aldrich, St. Louis, MO, USA), 0.02 mM folic acid (Sigma-Aldrich, St. Louis, MO, USA), 0.1 mM of 2-mercaptoethanol (Gibco, Grand Island, NY, USA), and 200 U/mL of IL-2 (Sigma-Aldrich, St. Louis, MO, USA). All cells were maintained at 37 °C and 5% CO_2_.

### 2.4. Human Blood Samples

Blood from healthy donors over the age of 18 was collected in accordance to a protocol approved by the Health Sciences North (HSN) Research Ethics Board (REB) (#18-061). Blood from treatment-naïve colorectal cancer patients was collected according to protocols approved by HSN REB (#19-022, 18-104). Immediately after collection, PBMC were isolated by Ficoll-Paque (GE Healthcare, Uppsala, Sweden) density gradient. PBMC were cryopreserved in the vapor phase of liquid nitrogen in vials containing 0.5–1 × 10^7^ cells/mL in a solution of 90% HI FBS and 10% dimethyl sulfoxide (DMSO, Corning, Manassas, VA, USA).

### 2.5. Immunoblotting

For Western blot experiments, 10^6^ cells/mL (3 mL in a 6-well plate) of THP-1 cells were differentiated to macrophages for 24 h in media containing 100 ng/mL phorbol 12 myristate 13 acetate (PMA, Sigma-Aldrich, St-Louis, MO, USA), after which cells were washed and rested in new media without PMA for an additional 24 h prior to treatment. For HCT 116, HT-29, A2780 and A2780R, 5 × 10^5^ cells/mL were seeded (2–3 mL in 6-well plate) and left to adhere overnight prior to treatment. Cryopreserved PBMC from 2 healthy individuals were thawed, seeded at 2 × 10^6^ cells/mL (2 mL in 12-well plate) in X-Vivo 15 (Lonza, Walkersville MD, USA) and rested overnight before treatment. All cells were subsequently treated with Ara-C/DS (1 µg/mL of DS loaded with 50 nM of Ara-C, or 50 µg/mL of DS loaded with 2.5 µM of Ara-C), its components, or 1 µg/mL 2′,3′-cGAMP (cGAMP, InvivoGen, San Diego, CA, USA) for 6 or 24 h.

Protein lysates were collected from cell lines using a 0.1% SDS lysis buffer containing 50 mM TRIS pH 7.4, 2 mM sodium orthovanadate, 1 mM sodium fluoride, and complete Protease Inhibitor (all chemicals from Fisher Scientific, Fairlawn, NJ, USA). Protein was quantified using a Pierce BCA assay (ThermoFisher Scientific, Rockford, IL, USA) and denatured at 95 °C for 5 min in 5X Lane Marker Reducing Sample Buffer (ThermoFisher Scientific, Rockford, IL, USA). Equal amounts of protein (15 µg for 24 h samples, 45 µg for 6 h samples) were separated on 12% SDS-PAGE and transferred onto a nitrocellulose membrane (BioRad, Mississauga ON). Blots were incubated overnight at 4 °C with antibodies (Cell Signaling Technology, Danvers, MA, USA) specific for either phosphorylated histone γ-H2AX (Ser 139, D7T2V) or P-TBK1 (Ser 127, D52C2), P-IRF3 (Ser 386, E7J86), and P-STING (Ser 366, D7C3S). Glyceraldehyde 3-phosphate dehydrogenase (GAPDH) (D4C6R) was used as a loading control. 2.5% bovine serum albumin (Sigma Aldrich, St Louis, MO, USA) and 5% milk (BioRad, Hercules, CA, USA) blocking buffers were used to dilute primary and secondary antibodies, respectively. Proteins were detected by either goat anti-rabbit IgG HRP-conjugated (ab205718 Abcam, Eugene, OR, USA) or goat anti-mouse IgG HRP-conjugated (ab6789 Abcam, Eugene, OR, USA) antibodies and electrochemiluminescence reagent (Cytiva ECL Select, Marlboro, MA, USA) using the FluorChem Q imaging system (Protein Simple, San Jose, CA, USA). Densitometry values were calculated within the FluorChem Q software, normalized to the GAPDH loading control, and represented relative to the non-treated control.

### 2.6. Sulforhodamine B (SRB) Assay

Inhibition of cancer cell growth by Ara-C/DS was determined by sulforhodamine B (SRB) colorimetric assay according to previously published protocols [35]. Briefly, cells were plated at 5 × 10^4^ cells/mL (100 µL in a 96-well plate), rested overnight, and treated with titrated doses of Ara-C or Ara-C/DS. After 48 h of incubation, cells were fixed in 10% trichloroacetic acid (TCA, Fisher Scientific, Fair Lawn, NJ, USA) and stained with 0.4% SRB dye (Alfa Aesar, Heysham, UK). After washing and drying, 200 μL of 10 mM Tris solution (Fisher Scientific, Fair Lawn NJ, USA) were added to the plates and the absorbance was read at 510 nm with the Synergy H4 Hybrid Microplate Reader (Agilent, Santa Clara CA, USA). The % growth was determined in relation to a 0% cell growth (untreated cells fixed at 0 h) and a 100% cell growth (untreated cells fixed at 48 h).

### 2.7. Immune Cell Ligands and 7-Aminoactinomycin D (7-AAD) Analysis

HT-29, HCT 116, A2780 and A2780R were seeded at 5 × 10^4^ cells/mL (1 mL in a 24-well plate) and rested overnight before treatment with Ara-C/DS (1 µg/mL of DS loaded with 50 nM of Ara-C), its components, or cGAMP (1 µg/mL) for 48 h. Cells were collected and stained with anti-HLA-ABC-FITC (MHC I, Biolegend, San Diego, CA, USA), anti-MICA/B-FITC (R&D Systems, Minneapolis, MN, USA) or anti-ULBP2/5/6-PE (R&D Systems, Minneapolis MN, USA) and analyzed using the Cytomics FC-500 (Beckman Coulter, Fullerton, CA, USA) where 10^4^ events were measured and singlet live cells were gated. Dead cells were identified by 7-AAD staining (Biolegend, San Diego, CA, USA). Data was analyzed using the CXP Analysis Software.

### 2.8. Tumor-Associated Antigen (TAA)-Reactive CD8 T Cell Determination

TAA-Reactive T cells were quantified using previously published methods [35,48]. PBMC from 3 colorectal cancer patients were seeded at 4 × 10^6^ cells/mL in a 24-well plate and expanded with 1 µg/mL PepMix™ Peptide Pools (JPT Peptide Technologies, Berlin, Germany) with or without Ara-C/DS (2 µg/mL of DS loaded with 100 nM Ara-C). Three different TAA PepMix™ common to colorectal cancer were tested, including mucin-1 (MUC1), human telomerase reverse transcriptase (hTERT), and carcinoembryonic antigen (CEA). PBMC were maintained in X-Vivo 15 media supplemented with IL-4 (5 ng/mL), IL-7 (5 ng/mL) and IL-2 (starting day 3, 40 U/mL) (all supplements from PeproTech, Rocky Hill, NJ, USA), with media changed every 2–3 days. After 12 days, PBMC were collected, washed, aliquoted, and re-stimulated with each individual TAA PepMix™ in the presence of Golgi Plug (1/1000 dilution, BD Biosciences, San Diego CA, USA) for 12 h. PBMC without re-stimulation served as a negative control and PBMC stimulated with 1 µg/mL of PMA and ionomycin (Sigma-Aldrich, St-Louis, MO, USA) served as a positive control. PBMC were stained for flow cytometry with anti-CD3-AF700, anti-CD8-PerCPCy5.5, and a fixable viable dye eFluor780 (Biolegend, San Diego, CA, USA) prior to permeabilization with a Cytofix/Cytoperm kit (BD Biosciences, San Diego, CA, USA) and staining with anti-IFN-γ-APC (Biolegend, San Diego, CA, USA). Data was analyzed on the CytoFLEX Flow Cytometer (Beckman Coulter, Fullerton, CA, USA) and using the Kaluza Analysis Software. Singlet viable CD3^+^ CD8^+^ cells were gated, from which the IFN-γ^+^ cell population was identified. Responders are defined as having a distinct population of IFN-γ^+^ CD8 T cells with a minimum 2-fold increase in cytokine-positive cells as compared to the unstimulated control, and had to have a positive response to the PMA/ionomycin control.

### 2.9. NK-92 Co-Cultures

HCT 116 were plated at 5 × 10^4^ cells/mL (100 µL in a 96-well plate) and rested overnight. Cells were left untreated or treated with Ara-C/DS (10 nM Ara-C, 0.2 µg/mL DS), with or without different targets: effector ratios of HCT116: NK-92 (1:0, 1:5, or 1:10). The total volume per well was 200 µL, and comprised of 50% complete RPMI-1640 (HCT 116 media) and 50% complete MEM Alpha (NK-92 media). After 48 h, the suspension NK-92 cells was removed from the plate by gently washing the wells, before fixing the adherent HCT 116 cells in 10% TCA. The SRB assay protocol was used to stain the HCT 116 and quantify their growth in the presence of NK-92 cells.

### 2.10. Statistical Analysis

Statistical analyses were performed in GraphPad Prism 5 using one- or two-way ANOVA with a Tukey or Bonferroni Post Hoc Test, respectively.

## 3. Results & Discussion

**Figure 2:**
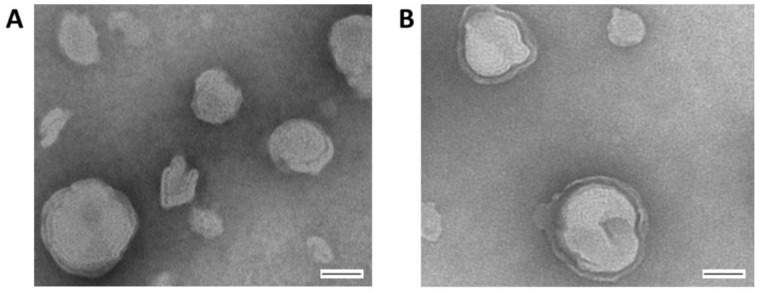
Transmission electron micrographs (TEM) of the cationic liposome. Solutions of the cationic liposome (0.5 µg/uL in ddH_2_O) were analyzed after incubation at room temperature for (**A**) 24 h, or (**B**) 2 weeks. The preparation was negatively stained with 3% phosphotungstic acid and imaging performed at 75 kV. Bar = 100 nm.

Prior to performing bioactivity assays, the structure of the liposomal formulation containing the aromatic mannose lipid, cholesterol, and DDAB (5:4:1 molar ratio) was imaged via TEM (Figure 2). This analysis confirmed that the above mixture in water formed a multilayer particle with an average size below 200 nm that is stable for at least 2 weeks, making it suitable for entrapping Ara-C, which is further supported by our previous report [35].

**Figure 3:**
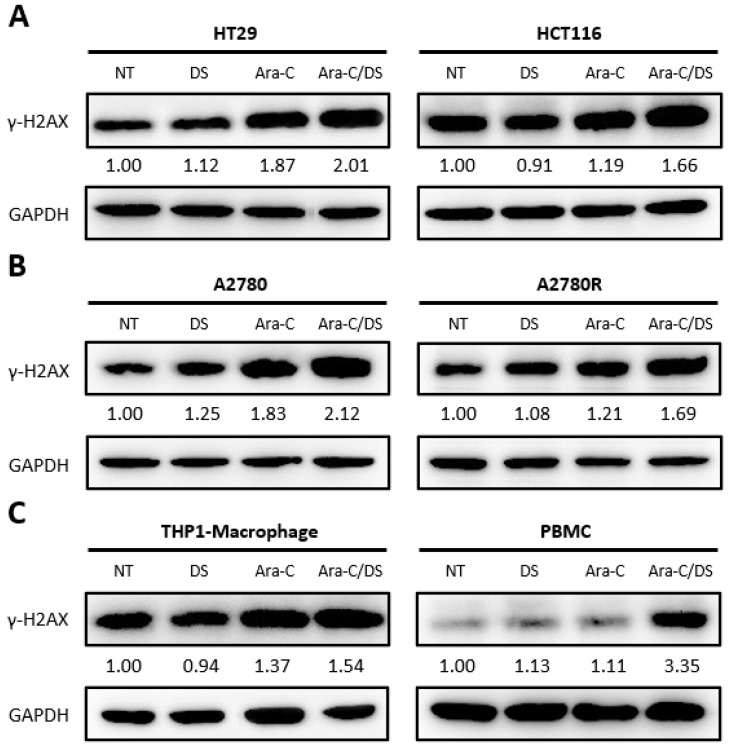
Assessment of DNA damage induction by Ara-C/DS and its components. Presented are immunoblots of histone γ-H2AX and GAPDH in (**A**) colorectal cells cell lines (HT-29 & HCT 116), (**B**) ovarian cancer cell lines (A2780 & A2780R), and (**C**) immunological cells (THP-1 derived macrophages & PBMC) after treatment for 24 h with Ara-C (50 nM), the DS (1 µg/mL), or their combination (Ara-C/DS). Results shown are representative of three replicate experiments. NT = non-treated. Densitometry values have been normalized to the GAPDH loading control, and are relative to the NT control. The uncropped bolts are shown in Supplementary Materials.

Ara-C, as a pyrimidine nucleoside chemotherapeutic, is incorporated into the genomic DNA during cellular replication where it prevents further nucleotide chain elongation, ultimately leading to stalled replication forks and the formation of DSB [36]. H2AX is a member of the histone H2A family, and its phosphorylation at serine 139 (γ-H2AX) in response to genomic DNA damage is an integral aspect of the DDR [49]. Detection of γ-H2AX is a proven approach for quantifying DNA DSB as its levels are directly associated with the degree of DNA damage [50]. Ara-C has previously been shown to upregulate γ-H2AX levels in both cancer and immune cells at doses as low as 100 nM [51]. γ-H2AX levels induced by the DS (1 µg/mL), Ara-C (50 nM), or their combination (Ara-C/DS) after 24 h were assessed in human ovarian cancer (A2780 & A2780R) and colorectal cancer (HT-29 & HCT 116) cell lines, as well as immunological human cells including THP-1 derived macrophages and PBMC (Figure 3). The general trend was that, compared to the non-treated controls, γ-H2AX levels were: (i) on par in cells treated solely with the DS, indicating that it does not cause DNA DSB; (ii) higher when treated with Ara-C; (iii) highest with the Ara-C/DS combination, further confirming the ability of the DS to improve intracellular Ara-C delivery.

**Figure 4:**
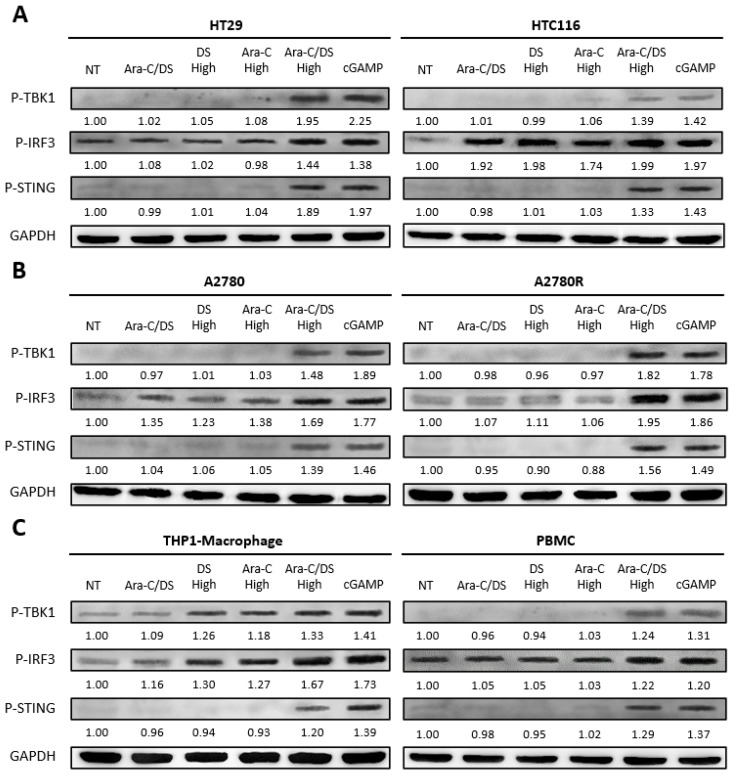
Assessment of cGAS-STING axis activation by Ara-C/DS and its components. Presented are immunoblots of P-TBK1, P-IRF3, P-STING, and GAPDH in (**A**) colorectal cells cell lines (HT-29 & HCT 116), (**B**) ovarian cancer cell lines (A2780 & A2780R), and (**C**) immunological cells (THP-1 derived macrophages & PBMC) after treatment for 6 h with standard dose Ara-C/DS (50 nM Ara-C, 1 µg/mL DS), high dose Ara-C (2.5 µM), high dose DS (50 µg/mL), the high dose Ara-C/DS combination (2.5 µM Ara-C, 50 µg/mL DS), or the cGAMP STING agonist (1 µg/mL) as a positive control. Results shown are representative of three replicate experiments. NT = non-treated. Densitometry values have been normalized to the GAPDH loading control, and are relative to the NT control. The uncropped bolts are shown in Supplementary Materials.

With the ability of the DS to improve Ara-C induced DNA damage confirmed, cGAS-STING axis activation was next evaluated via immunoblot analyses for P-IRF3, P-TBK1, and P-STING levels. The cell lines and PBMC were treated for 6 h with the same Ara-C/DS concentration (50 nM Ara-C, 1 µg/mL DS), in addition to 50x higher doses (2.5 µM Ara-C, 50 µg/mL DS), either alone or in combination (Figure 4). The 6 h time point was chosen based on a previous report in which cGAS-STING axis activation by DNA-damaging chemotherapeutics occurred in as little as 2 h [52]. Potential cGAS-STING axis activation with the lower Ara-C/DS treatment was only observed in the HCT 116 colorectal cancer cell line, where elevated levels of P-IRF3 were observed. However, neither P-TBK1 nor P-STING were observed, suggesting the potential of non-cGAS-STING dependent activation of IRF3. In each case, the higher Ara-C/DS dose resulted in elevated levels of all three cGAS-STING axis markers which were approximately on par with those induced by the cGAMP (1 µg/mL) dinucleotide positive control. In nearly all cases, neither the high doses of the DS or Ara-C induced phosphorylation of any of the markers, with the exception of the THP-1 derived macrophages, where elevated levels of P-IRF3 and P-TBK1 were observed relative to the untreated control. While not unexpected for the Ara-C treatment, cGAS-STING axis activation by the DS may be explained by reports on the ability of cationic liposomes and virus-like particles to activate STING independent of cGAS and cGAMP in a process known as unscheduled membrane fusion [53,54]. Nevertheless, the fact that cGAS-STING axis activation is only observed with the higher dose of Ara-C when entrapped in the DS further demonstrate the enhanced intracellular delivery that the Ara-C/DS combination offers. The fact that cGAS-STING activation was not observed with the lower Ara-C/DS dose may be a consequence of the sensitivity of the immunoblot assay, or that the 6 h time point may need to be extended to match that of the DNA damage assay, where this dose of the treatment showed definitive ability to cause DNA DSB (Figure 3). Future studies will look to address this. Overall, these are the first results to link the DNA damaging activity of the Ara-C chemotherapeutic with cGAS-STING axis activation, further adding to the list of chemotherapeutics and DDR inhibitors reported to have this activity [26,27,28]. The data is also in line with a recent report showing that nanoparticles loaded with platinum and photosensitizing agents induced DNA damage in gastric cancer, leading to the activation of the cGAS-STING pathway and eventual antitumor immunity [55]. This further confirms that DNA-damaging agents and nanoparticle carriers offer a promising strategy to potentially combat immune refractory tumors.

**Figure 5:**
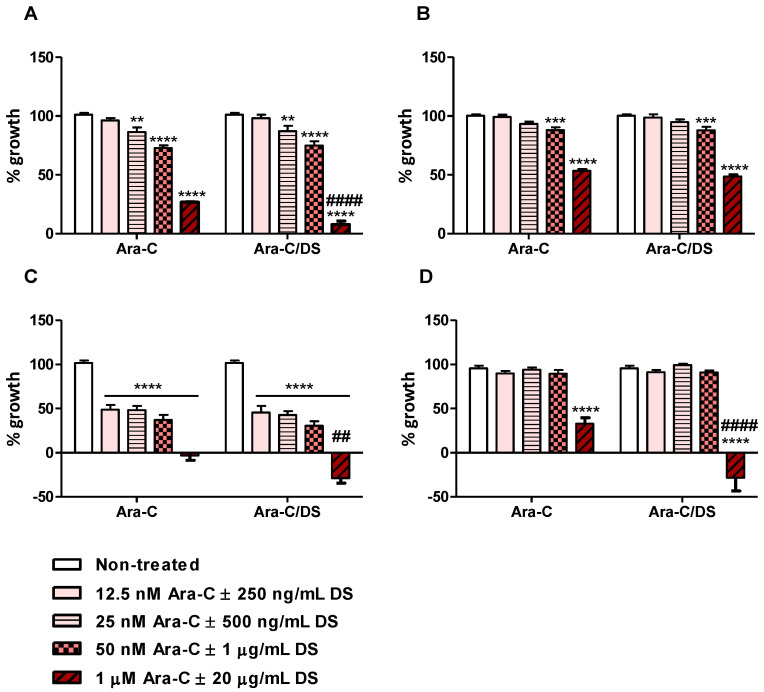
Cytostatic potential of AraC/DS. Cell growth of (**A**) HCT 116, (**B**) HT29, (**C**) A2780 and (**D**) A2780R treated with titrated doses of AraC or AraC/DS for 48 h was assessed by SRB assay (data presented as the average of two independent experiments performed in triplicate, *n* = 6 ± SEM). Statistical differences were calculated by twoway ANOVA with Bonferroni multiple comparison post hoc tests where **** *p* < 0.0001, *** *p* < 0.001, and ** *p* < 0.01 and compared each doses to the non-treated condition, and #### *p* < 0.0001 and ## *p* < 0.01 compared Ara-C/DS to Ara-C at the respective dose.

Although the immunotherapeutic potential of Ara-C/DS is linked to its DNA-damaging ability, with cGAS-STING axis activation being dose dependent, we importantly are using low Ara-C doses as to not impair or kill the immune cells. Ara-C up to 500 nM, alone or entrapped in DS at doses up to 10 µg/mL, was already shown to have negligible toxicity (<10% cell death) on PBMC and THP-1 [35]. Doses up to 100 µg/mL of DS, and Ara-C/DS (500 nM Ara-C, 10 µg/mL DS) were also shown to be hemocompatible, and did not induce red blood cell lysis [35]. Here, the impact of Ara-C/DS on the cell growth of the colorectal and ovarian cancer cell lines was investigated. Matching the dose used in the DNA damage analysis (Figure 3), Ara-C/DS at a dose of 50 nM Ara-C and 1 µg/mL DS impeded cell growth of HCT 116, HT-29 and A2780, while the cisplatin resistant cell line A2780R showed cross-chemotherapy resistance, with normal growth being observed at this dose (Figure 5). For the two colorectal cancer cell lines, doses ≤25 nM of Ara-C were required in order to observe normal cell growth, while the growth of A2780 was still reduced by nearly half even at the dose of 12.5 nM of Ara-C. At the highest dose of Ara-C used in this assay (1 µM), the growth of all tested cell lines, including the resistant A2780R, was significantly reduced. While DS doses below 1 µg/mL did not impact the cytostatic properties of Ara-C in any of the cell lines, pairing 1 µM of Ara-C with 20 µg/mL of DS further decreased the cell growth of HCT 116, A2780, and A2780R. This may be partly due to high doses of charged liposomes impacting cell adhesion on the negatively charged tissue culture-treated plates. In the case of A2780 and A2780R, the highest tested dose of Ara-C/DS led to a negative cell growth indicative of cell death. Interestingly, in Figure 4, only marginal differences were observed in the amount of protein collected for the different treatments, suggesting that 2.5 µM of Ara-C was not directly killing the cells at 6 h. However, the SRB data (Figure 5) strongly imply that this dose would eventually induce cell death at longer time points.

**Figure 6:**
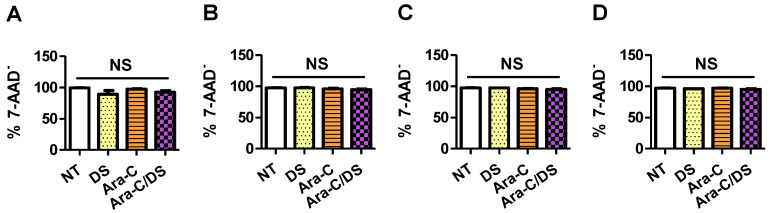
Cytotoxic potential of Ara-C/DS at low dose. Cell viability of (**A**) HCT 116, (**B**) HT-29, (**C**) A2780 and (**D**) A2780R treated with Ara-C/DS, or its components (50 nM Ara-C, 1 µg/mL DS) was assessed by 7-AAD stain after 48 h (data presented as the average of six independent experiments, *n* = 6 ± SEM). Statistical differences were calculated by one-way ANOVA with Tukey’s post hoc tests. NS: not significantly different.

Importantly, the SRB assay measures cell proliferation by staining cellular protein content [56]. Therefore, a cell growth between 0 and 100% may not be correlated to cell death. Instead, processes which are known to occur following the DDR, such as cell cycle disruption and senescence, may lead to reduced cell proliferation without killing the cells [57]. Therefore, the percentage of dead cells following a 48 h treatment with Ara-C/DS (50 nM Ara-C, 1 µg/mL DS) or its components was assessed by flow cytometry analysis of 7-AAD stained cells. 7-AAD is a membrane impermeant dye that is excluded from live cells and can therefore be used to identify dead or dying cells that have compromised membranes. This experiment aimed to determine if the Ara-C and DS doses used in the DNA damage experiments (Figure 3), which led to the reduced cell growth of HCT 116, HT-29, and A2780 (Figure 5), also led to cell death. For all the tested cell lines, no statistical differences were observed between the different treatment groups when quantifying the number of dead cells by 7-AAD (Figure 6). This data importantly confirms that low dose treatment of Ara-C/DS does not lead to direct cell death, but impacts cell replication, further supporting the use of Ara-C as an immunostimulant with limited concomitant toxicity linked to the DDR. As a crucial step in the repurposing of Ara-C for immunotherapy, future studies will carefully evaluate the toxicity of Ara-C/DS using animal models to establish in vivo doses capable of inducing immune responses without killing or impairing the immune cells responsible for tumor clearance.

**Figure 7:**
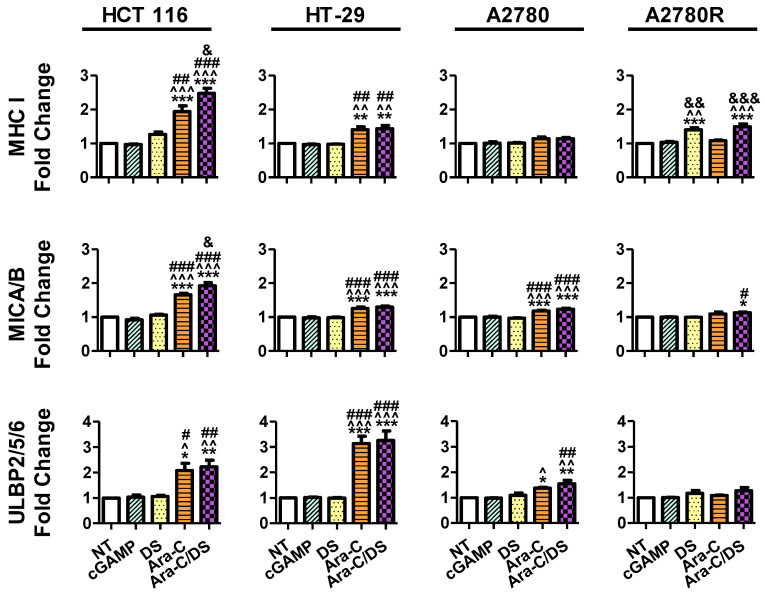
Change in surface expression of T and NK cell ligands following Ara-C/DS treatment. Cells were treated with Ara-C/DS, its components (50 nM Ara-C, 1 µg/mL DS) or cGAMP (1 µg/mL) for 48 h prior to analysis of MHC I, MICA/B and ULBP2/5/6 surface expression by flow cytometry (fold change calculated relative to non-treated cells, data represented as the average of three independent experiments, ± SEM). Statistical analysis performed with a one-way Anova with a Tukey’s HSD where *** *p* < 0.001, ** *p* < 0.01 and * *p* < 0.05 as compared to non-treated (NT), ^^^ *p* < 0.001, ^^ *p* < 0.01 and ^ *p* < 0.05 as compared to cGAMP, ### *p* < 0.001, ## *p* < 0.01 and # *p* < 0.05 as compared to DS, and &&& *p* < 0.001, && *p* < 0.01 and & *p* < 0.05 as compared to Ara-C.

**Figure 8:**
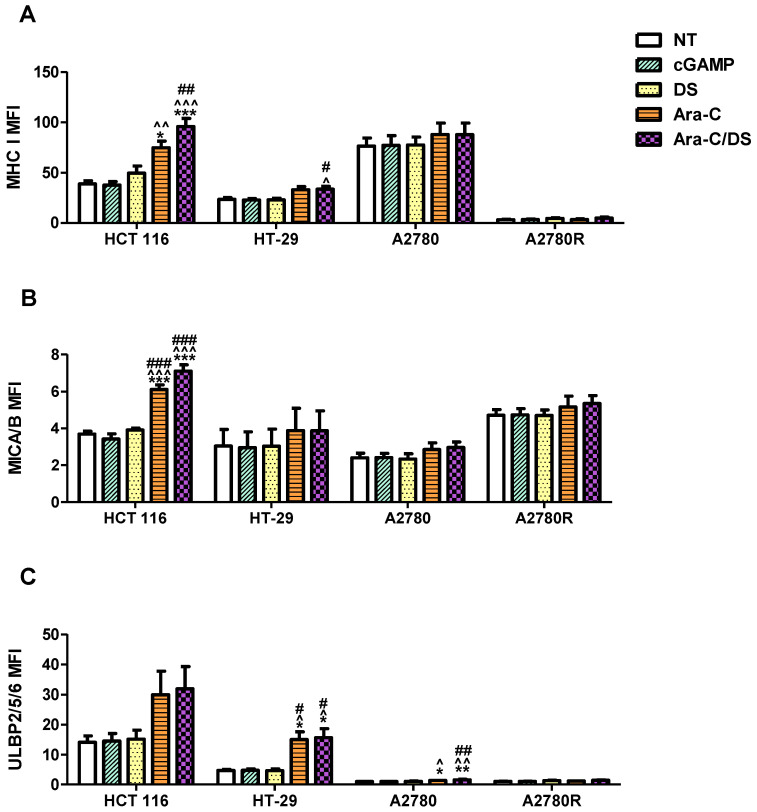
Comparison of the surface expression of immune ligands on HCT 116, HT-29, A2780 and A2780R. Cells were treated with Ara-C/DS, its components (50 nM Ara-C, 1 µg/mL DS) or cGAMP (1 µg/mL) for 48 h prior to analysis of (**A**) MHC I, (**B**) MICA/B and (**C**) ULBP2/5/6 surface expression by flow cytometry. The reported MFI was normalized to a non-stained control (data represented as the average of three independent experiments, ± SEM). Statistical analysis performed with a one-way Anova with a Tukey’s HSD for each respective cell line where *** *p* < 0.001, ** *p* < 0.01 and * *p* < 0.05 as compared to non-treated (NT), ^^^ *p* < 0.001, ^^ *p* < 0.01 and ^ *p* < 0.05 as compared to cGAMP, ### *p* < 0.001, ## *p* < 0.01 and # *p* < 0.05 as compared to DS.

While clear STING activation was observed in all cell types at the higher Ara-C/DS dose, activation at the lower non-cytotoxic dose at the chosen 6 h time point was only evident in the HCT 116 cell line. As previously mentioned, lengthening assay time to match that of the DNA damage assay may be required to observe changes in the STING pathway at lower doses. To better understand the mechanism of low dose Ara-C/DS, downstream targets of DDR-dependent STING activation, including upregulation of various immune cell ligands, were investigated. MHC I is part of the MHC family of molecules that are often downregulated on cancer cells as a way to escape CD8 T cell recognition [17,58,59]. Enhancing surface expression of MHC I has therefore been identified as a major cancer immunotherapy target to improve both TAA presentation and T cell recognition, with evidence showing that STING activation leads to increased MHC I expression through the release of type I IFN or the activation of NF-κB [17]. Alternatively, MICA/B (MHC class I chain-related protein A and B) and ULBP1-6 (UL16-binding proteins 1–6) are important cell surface ligands of the natural-killer group 2, member D (NKG2D) receptor and function as a “kill” signal for NK cells [60]. Both direct STING agonists and DNA damaging agents have been proposed as enhancers of anticancer NK cell activity, which are in part mediated through changes in NKG2D ligands [61]. Here, the surface expression of MHC I, MICA/B, and ULBP2/5/6 was assessed on HCT 116, HT-29, A2780 and A2780R following low dose treatment with Ara-C/DS (50 nM Ara-C, 1 µg/mL DS), as compared to the STING agonist cGAMP (1 µg/mL) (Figure 7 and Figure 8).

First, the fold change in surface ligands following treatment was assessed as compared to the receptor levels of the non-treated control for each respective cell lines (Figure 7). Unexpectedly, the cGAMP did not enhance the expression of any of the ligands at a concentration of 1 µg/mL (1.4 µM). To our knowledge, the modulating role of cGAMP on immune cell ligands expression for the cell lines used herein has not been previously assessed. However, cGAMP was shown to increase MHC I expression on a variety of melanoma cell lines, albeit using a higher 10 µg/mL [62]. cGAMP was also shown to increase ULBP2/5/6 expression on pancreatic cancer cell lines at a concentration of 4 µg/mL [63]. Therefore, it is possible that the dose used in our study to activate the cGAS-STING axis was still too low to observe a significant change in surface protein expression. As expected based on the mechanistic studies presented above, DS alone also had no effect on ligand expression, aside from a 1.4-fold increase in MHC I on A2780R. Ara-C increased all three ligands on both HCT 116 and HT-29, and increased MICA/B and ULBP2/5/6 on A2780. MHC I expression on HCT 116 and A2780R, and MICA/B expression on HCT 116 were also further enhanced by entrapping Ara-C within DS. Overall, the trends in Figure 7 coincide with the DNA damage data shown in Figure 3, where (i) DS alone has little to no effect, (ii) Ara-C induces DNA damage and immune ligand surface expression upregulation, and (iii) DS further enhances the responses to Ara-C. Interestingly, the changes in surface expression of the various proteins vary greatly depending on the different cell lines. Ara-C/DS induced the greatest 2.5-fold change in MHC I expression on HCT 116 as compared to a 1.5-fold change on HT-29 and A2780R, and a non-significant 1.1-fold change on A2780. HCT 116 also had the greatest increase in MICA/B expression (1.9-fold change), as compared to HT-29, A2780, and A2780R (1.3-, 1.2-, and 1.1-fold change, respectively). However, ULBP2/5/6 was most strongly upregulated in HT-29 (3.3-fold change), followed by HCT-116 (2.2-fold change) and A2780 (1.6-fold change), with a non-significant 1.3-fold change on A2780R. Overall, both A2780 and A2780R were less sensitive to the activity of Ara-C/DS as compared to the colorectal cancer cell lines, however due to the small number of cell lines used, no conclusion can be drawn concerning a potential link between cancer type and immune cell ligand modulation. Future work will aim to screen a wider panel of cell lines to answer this question.

As an additional consideration, there is also a wide difference in the overall expression of the surface proteins depending on the cell line used, as indicated by varying mean fluorescence intensity (MFI, normalized to non-stained cells) (Figure 8). Of note, while the fold change in markers remains consistent across experiments (Figure 7), the MFI is more variable which is at least in part due to small changes in flow cytometer calibration, but also because higher passages of the cells may lead to phenotypic differences [64]. While the trends observed in Figure 8 match those seen in Figure 7, it becomes clear that while Ara-C/DS may double, or even triple the levels of surface proteins, the overall expression of immune ligands remains entirely dependent on the initial levels found on the cells. For example, ULBP2/5/6 expression, as assessed by MFI (Figure 8), is significantly increased in both HT-29 and A2780, however the overall protein expression is far lower on A2780. Similarly, HT-29 had the greatest fold increase in ULBP2/5/6 expression (3.3-fold increase, Figure 7), however the overall expression of this protein remains highest on HCT 116 (Figure 8). It is well established that cancer cells can regulate immune ligands as a way to escape immune attacks, including downregulation or shedding of the ligands which explains the variation seen across the different cell lines [60,65,66]. Unsurprisingly, the parental A2780 cell line and its cisplatin resistant A2780R counterpart show very different ligand profile, further confirming potential phenotypic changes over time which may happen in response to repeated therapy and resistance development. Cancer phenotype may therefore play a considerable role in the efficacy of STING-based therapies. Overall, while the threshold of surface markers required to produce targeted T and NK cell responses in vivo remains unclear, it is expected that cancers with the highest expression of MHC I, MICA/B, and ULBP2/5/6 would respond best to immunotherapy. Nevertheless, the data supports that the DDR induced by Ara-C and Ara-C/DS is essential to produce an observable increase in T and NK cell ligands on cancer cells in vitro. The data coincides with previous reports showing that Ara-C increases expression of the NKG2D ligand ribonucleic acid export 1 (RAE1) through the DDR [67], while other genotoxic drugs also enhanced NKG2D ligand expression on cancer cells [68]. Unsurprisingly, the DDR has also previously been shown to upregulate MHC I [69]. Notably, the chemotherapeutic agent cisplatin, was shown to both activate STING and upregulate MHC I expression on the surface of mouse ovarian cancer cell lines [70]. Our data, alongside literature reports, reinforce that activation of STING through the DDR may improve tumor immunogenicity, with subsequent immune-clearance of the cancerous cells through increases in immune-based ligands that act as “kill” signal for both T and NK cells. This process has the potential to improve both infiltration and activity of the immune cells within the immunosuppressive TME.

**Figure 9:**
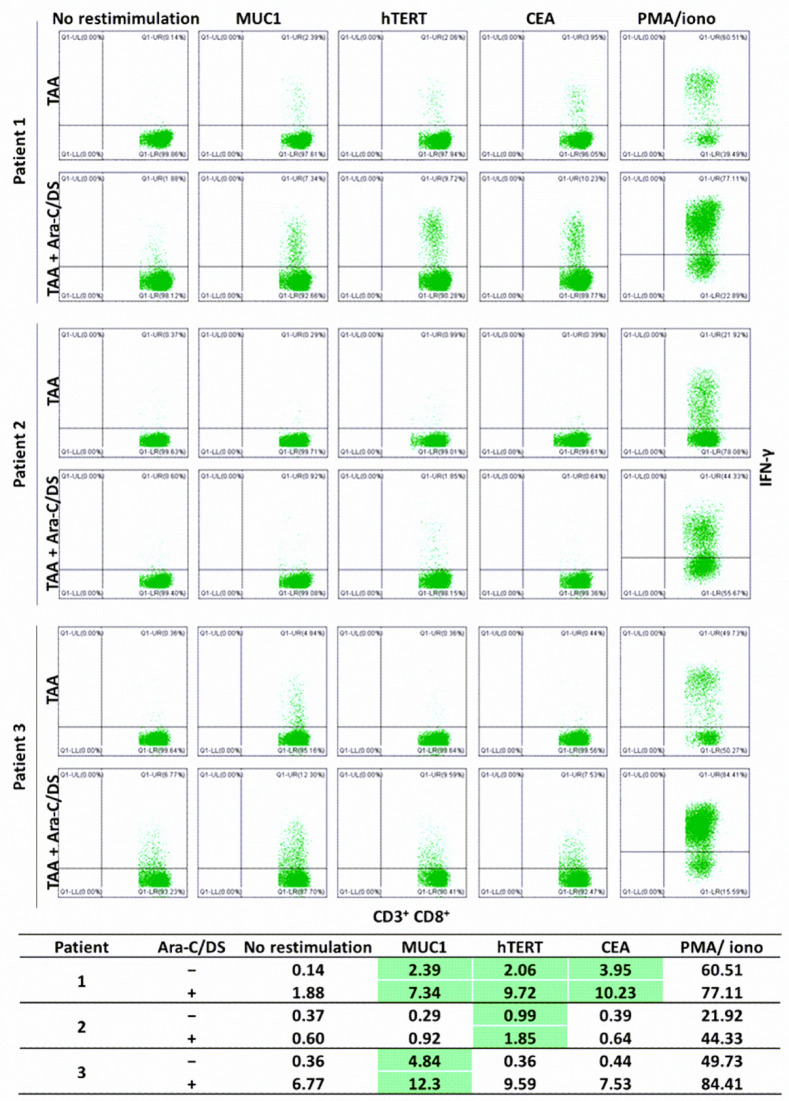
TAA-specific IFN-γ^+^ CD8 T cell responses in PBMC from 3 colorectal cancer patients. PBMC were incubated with TAA PepMix with or without Ara-C/DS (100 nM Ara-C, 2 μg/mL DS) and re-stimulated with TAA PepMix on day 12. After 12 h, CD8 T cells were analyzed for intracellular IFN-γ by flow cytometry. PMA/ionomycin was used as a positive control and unstimulated cells were used as a negative control. IFN-γ^+^ CD8^+^ percentages are summarized in the table where responders are highlighted in green.

Next, based on the mechanistic data showing cGAS-STING activation in immune cells (Figure 4), we aimed to assess the role of Ara-C/DS in activating T and NK cells. Using an established protocol [48], antigen-reactive T cells from colorectal cancer patients were expanded and quantified in response to TAA (Figure 9). To do so, PBMC were first treated with JPT’s PepMix™ Peptide Pools for selected TAA, alone or in the presence of Ara-C/DS. PepMix™ Peptide Pools are a mixture of overlapping peptides covering the entire TAA protein sequences, allowing for stimulation of PBMC independently of their human leukocyte antigen (HLA). CEA, MUC1 and hTERT were chosen as they are common TAA overexpressed in colorectal cancer [71,72]. After 12 days of incubation, the PBMC were collected, aliquoted, and restimulated with each TAA PepMix™, allowing for quantification of antigen-reactive CD8 T cells as measured by intracellular IFN-γ. It was identified that patient 1 responded to all three TAA, patient 2 only responded to hTERT, and patient 3 only responded to MUC1, as determined by a clear defined IFN-γ^+^ population with at least doubling of the responses as compared to non-restimulated cells. In all cases, Ara-C/DS increased the percentage of IFN-γ^+^ CD8 T cells in response to TAA. In patient 1, a 3.1-fold increase, 4.7-fold increase and 2.6-fold increase were observed in Ara-C/DS-treated cells combined with MUC1, hTERT and CEA, respectively, as compared to the TAA alone. In patient 2 and 3, Ara-C/DS had a 1.9-fold and 2.5-fold increased response to hTERT and MUC1, respectively. Ara-C/DS was also shown to increase the responses when combined with PMA/ionomycin. This data is in accordance with our previously published findings showing that Ara-C/DS improves responses to viral antigens [35]. The background levels of IFN-γ^+^ CD8 T cells in non-restimulated cells ranged from 0.14–0.37% without Ara-C/DS, but was increased to 0.60–6.77% with Ara-C/DS. This is somewhat unsurprising, as in vivo STING activation is known to lead to the cross-priming of tumor-reactive T cells [8,73]. While the ex vivo effects in PBMC are less defined, a recent study showed that STING agonists enhance cytokine responses in PBMC-derived dendritic cells (DC), which in turn can modulate CD8 T cell responses [74]. In the model used here, the antigens would be uptaken by APC, including DC, and presented to the T cells, with the STING activation in APC (Figure 4) further activating T cells via the release of type I IFN [18]. This results in greater priming of the T cells at 12 days, with peptide restimulation leading to enhanced memory responses in the presence of Ara-C/DS. While this data is promising, it is important to note that T cell responses are only useful if they are specific and directed at the cancer cells, underlining the importance of also upregulating MHC I surface expression on cancer cells (Figure 7). While measuring T cell-mediated cytotoxicity towards cancer cells will be required to confirm our findings, the data presented above continue to suggest that Ara-C/DS can modulate the immune system in a STING-dependant manner.

**Figure 10:**
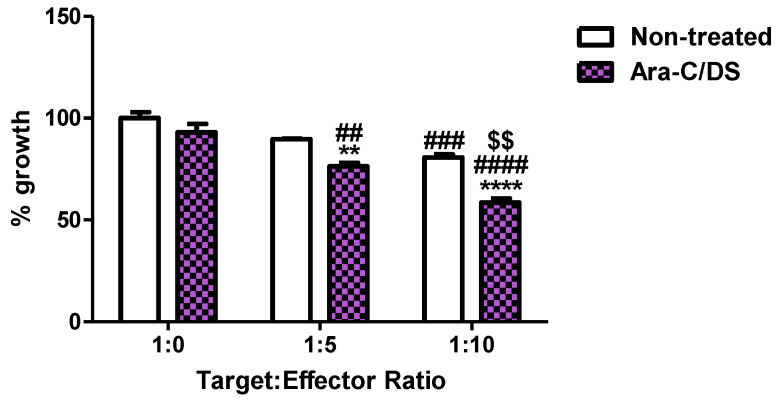
NK-92 cytotoxicity in a co-culture model with HCT 116. NK-92 and HCT 116 were co-cultured at various target:effector cell ratios alone or in the presence of Ara-C/DS (10 nM Ara-C, 0.2 µg/mL DS). After 48 h, NK-92 cells were removed and the growth of HCT 116 was measured using the SRB assay (data represented as a triplicate ± SEM). Statistical analysis was performed using a 2-way ANOVA with Bonferroni post hoc test where **** *p* < 0.0001 and ** *p* < 0.01 compares Ara-C/DS treated cells to non-treated cells for the respective target:effector ratios; #### *p* < 0.0001, ### *p* < 0.001 and ## *p* < 0.01 compares the 1:5 and 1:10 target: effector ratios to the cultures devoid of NK-92 (1:0 ratio) for each respective treatment; and $$ *p* < 0.01 compares the 1:10 target: effector ratios to the 1:5 target: effector ratios for each respective treatment.

Finally, the activity of Ara-C/DS on NK cell cytotoxicity was assessed in a preliminary model co-culturing the NK cell line NK-92 with HCT 116 (Figure 10). Due to Ara-C/DS being cytostatic, this assay used a dose of 10 nM of Ara-C entrapped in 0.2 µg/mL of DS which was identified as having no impact on HCT 116 proliferation (Figure 5). Figure 10 shows that the growth of HCT 116 is significantly reduced when co-cultured with NK-92 at a target: effector ratio of 1:10, but not at a ratio of 1:5. However, when treated with Ara-C/DS, the growth was statistically decreased at the 1:5 and 1:10 target: effector ratios, but not when NK-92 cells were omitted, suggesting that the HCT 116 are being killed by NK-92 as opposed to their growth being inhibited by Ara-C/DS. As with untreated cells, the 1:10 ratio led to the greatest reduction in HCT 116 cells. Furthermore, both the 1:5 and 1:10 target:effector cultures treated with Ara-C/DS led to significantly reduced growth of HCT 116 as compared to non-treated cultures, cementing the role of Ara-C/DS as a stimulator of NK cells that is also capable of enhancing the presence of NKG2D ligands on HCT 116 (Figure 7). Of note, the SRB assay, as previously mentioned, only measures reduction in cell growth and not the actual number of cells which are dying. However, a recent report confirmed that cGAMP enhanced NK-92 cytotoxicity against pancreatic cancer cells lines [63], suggesting that Ara-C/DS may function similarly. The activity of STING agonists has been further confirmed to be NK cell-dependent in various murine cancer models of the brain, skin, breast and colon [19,20,75]. To better assess the interactions between NK and cancer cells treated with Ara-C/DS, future studies will use lactate dehydrogenase (LDH) release to measure cytotoxicity, while concurrently measuring the levels of NKG2D on NK cells, its associated ligands on tumor cells, as well as soluble ligands such as MICA/B which have been shed in the media.

## 4. Conclusions

Overall, this work demonstrates for the first time that the immunomodulatory activity of Ara-C/DS is linked, in part, to its ability to activate the cGAS-STING signaling axis. Entrapment of low doses of Ara-C in the mannosylated cationic liposomal DS improved the ability of the chemotherapeutic to induce DNA DSB in various ovarian and colorectal cancer cell lines, as well as THP-1 derived macrophages and PBMC (Figure 3). This DNA damage translated into cGAS-STING axis activation in a dose-dependent fashion, with phosphorylation of TBK1, IRF3, and STING observed (Figure 4). We confirmed that the low dose of Ara-C (50 nM) entrapped in the DS, while cytostatic (Figure 5), does not lead to cell death (Figure 6), supporting its use as an immunostimulant devoid of DDR associated toxicity. To various degrees, Ara-C/DS was able to enhance cancer surface expression of T and NK cell ligands MHC I, MICA/B, and ULBP2/5/6 (Figure 7 and Figure 8), potentially leading to enhanced immune cell clearance through increased tumor immunogenicity. Enhanced IFN-γ^+^ CD8 T cell memory responses to TAA were also observed in PBMC isolated from colorectal cancer patients when treated with Ara-C/DS (Figure 9). Finally, Ara-C/DS improved the cytotoxic potential of the NK cell lines NK-92 against target HCT 116 cells (Figure 10), which may at least in part be due to the upregulation of MICA/B and ULBP2/5/6 on HCT 116. Taken together, these results support that Ara-C/DS mediated cGAS-STING axis activation leads to improved antigen presentation and subsequent cytotoxic lymphocyte responses. While very promising, it is important to note that STING activation can also play a dual role in cancer immunity, particularly when dysregulated chronic activation occurs, leading to auto-inflammation, TME enhancement, and eventual tumor progression [9,76,77]. This highlights the important of performing in vivo studies to paint a comprehensive picture of the careful balance required to activate the immune system without causing chronic inflammation in order to achieve cancer clearance.

Current work is aiming to confirm the immunotherapy efficacy of Ara-C/DS in cancer models both in vivo and ex vivo. Using syngeneic mouse models of ovarian and colorectal cancers, some of which are resistant to immunotherapy, we are investigating the immunomodulatory potential of Ara-C/DS as compared to checkpoint inhibitors by measuring the survival of mice, as well as phenotyping lymphoid organs and tumors after treatment. Importantly, these studies allow for the assessment of the essential tumor microenvironment and its role in treatment efficacy. Additionally, co-cultures of patient-derived tumor organoids with patient-matched PBMC are being developed to assess the killing potential of cytotoxic lymphocytes treated with Ara-C/DS. These experiments also aim to compare Ara-C/DS to STING agonists to confirm the potential benefits of exploiting the DDR for STING activation, as the data presented herein suggests that Ara-C has a greater impact on the upregulation of immune ligands on cancer cells as compared to cGAMP. We are additionally investigating a variety of other agents involved in the DDR as potential STING modulators. Together, these experiments will provide a better understanding of the immunotherapeutic potential of Ara-C/DS, and other drugs involved in the DDR, to treat immune refractory tumors that respond poorly to current immune-based treatments.

## Data Availability

All data is contained in the manuscript and accompanying Supplementary Materials.

## Supplementary Materials

The following supporting information can be downloaded at: https://www.mdpi.com/article/10.3390/pharmaceutics14122710/s1, which contains all original images for the Western blot analyses.
