# Supplementary material for: Exploiting the DNA Damaging Activity of Liposomal Low Dose Cytarabine for Cancer Immunotherapy
Source: Pharmaceutics. 2022 Dec 3;14(12):2710. doi: 10.3390/pharmaceutics14122710 (PMC9782803; doi:10.3390/pharmaceutics14122710)

SUPPLEMENTAL INFORMATION

Exploiting the DNA damaging activity of liposomal low dose cytarabine for cancer immunotherapy

Jordan D. Lewicky [1,†], Alexandrine L. Martel [1,†], Nya L. Fraleigh [1], Emilie Picard [1,2], Leila Mousavifar [3], Arnaldo Nakamura [4], Francisco Diaz-Mitoma [5], René Roy [3,*], Hoang-Thanh Le [1,5,*]

[1] Health Sciences North Research Institute, 56 Walford Road, Sudbury, ON P3E 2H2, Canada
[2] Cancer Research Center of Lyon, 28 rue Laennec, 69008 Lyon, France
[3] Glycosciences and Nanomaterial Laboratory, Université du Québec à Montréal, P.O. Box 8888, Succ. Centre-Ville, Montréal, QC H3C 3P8, Canada
[4] Armand-Frappier Santé Biotechnologie Research Centre, Institut National de la Recherche Scientifique, 531 Boulevard des Prairies, Laval, QC H7V 1B7, Canada
[5] Medicinal Sciences Division, NOSM University, 935 Ramsey Lake Road, Sudbury, ON P3E 2C6, Canada
† These authors contributed equally to this work.
* Correspondence: roy.rene@uqam.ca (R.R.); hle@hsnri.ca (H.-T.L)

Figure 1 Raw Western Blot Images

A2780 & A780R, 24 hours, γ-H2AX…………………………………………………… 3
A2780 & A2780R, 24 hours, GAPDH…………………………………………………..… 4

HCT 116 & HT-29, 24 hours, γ-H2AX…………………………………….…………… 5
HCT 116 & HT-29, 24 hours, GAPDH…………..…………………………….……………….. 6

PBMC & THP1-Macrophage, 24 hours, γ-H2AX………………….…………………….. 7
PBMC & THP1-Macrophage, 24 hours, GAPDH……………………………….…….. 8

Figure 2 Raw Western Blot Images

A2780, 6 hours, P-TBK1…………………………..………………………………………… 9
A2780, 6 hours, P-IRF3…………………………………………………….……………….. 10
A2780, 6 hours, P-STING……………………………………………………………….….. 11
A2780, 6 hours, GAPDH……………………………….……………………………………... 12

A2780R, 6 hours, P-TBK1……………………………………………………….……….. 13
A2780R, 6 hours, P-IRF3……………………………………………………..…………... 14
A2780R, 6 hours, P-STING…………………………..……………………………….... 15
A2780R, 6 hours, GAPDH……………………………………………..…………………… 16

HCT 116, 6 hours, P-TBK1……………………………………………………………………. 17
HCT 116, 6 hours, P-IRF3……………………………………………………………………... 18
HCT 116, 6 hours, P-STING…………….……………………………...…………………... 19

HCT 116, 6 hours, GAPDH………………………………………….……………………... 20

HT-29, 6 hours, P-TBK1…………………………………………….……………………. 21
HT-29, 6 hours, P-IRF3………………………………………………………………….….. 22
HT-29, 6 hours, P-STING………………………………………………………………….. 23
HT-29, 6 hours, GAPDH…………………………………………………………………... 24

THP1-Macrophage, 6 hours, P-TBK1…………………………………………………….. 25
THP1-Macrophage, 6 hours, P-IRF3……………………………………………………... 26
THP1-Macrophage, 6 hours, P-STING…………………………………………………... 27
THP1-Macrophage, 6 hours, GAPDH…………………………………………………… 28

PBMC, 6 hours, P-TBK1………………………………………………………………… 29
PBMC, 6 hours, P-IRF3…………………………………………………………………….. 30
PBMC, 6 hours, P-STING………………………………………………………………….. 31
PBMC, 6 hours, GAPDH…………………………………………………………………... 32

Figure 1 Raw Western Blot Images

A2780 & A2780R, 24 hours, γ-H2AX:

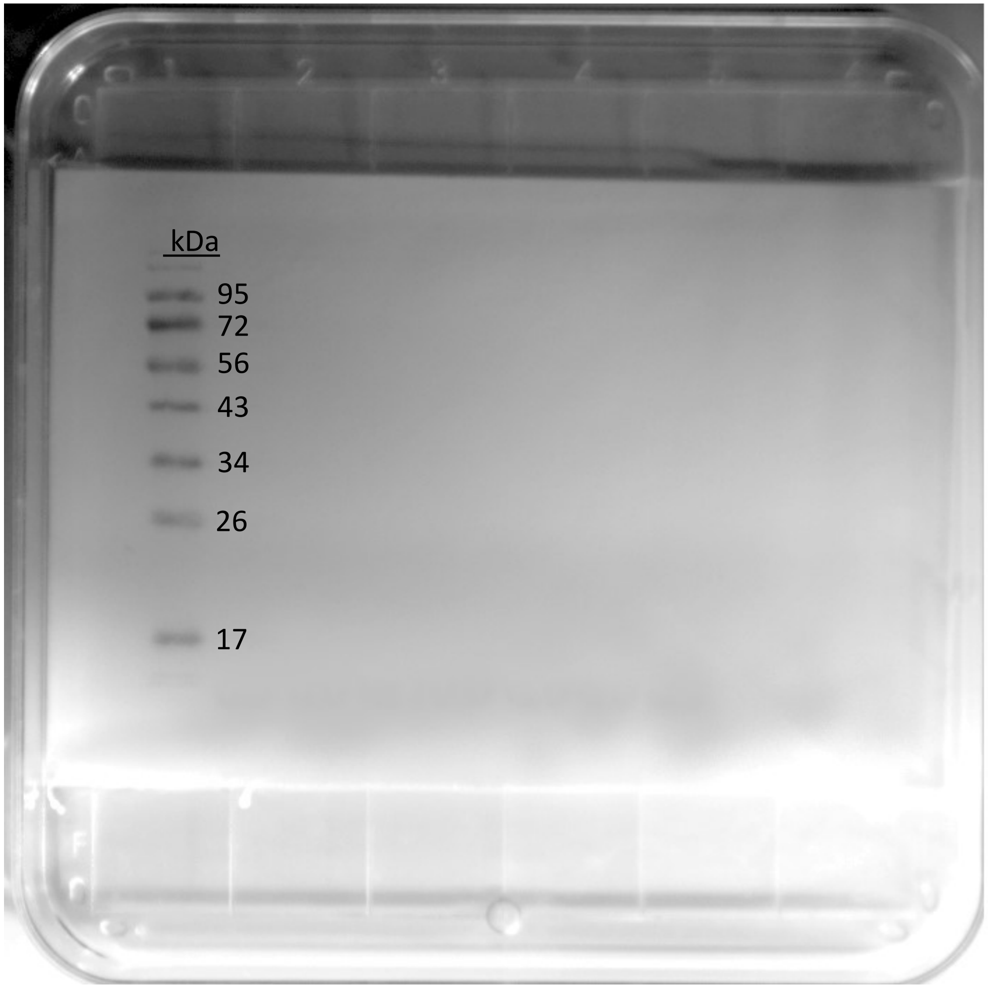

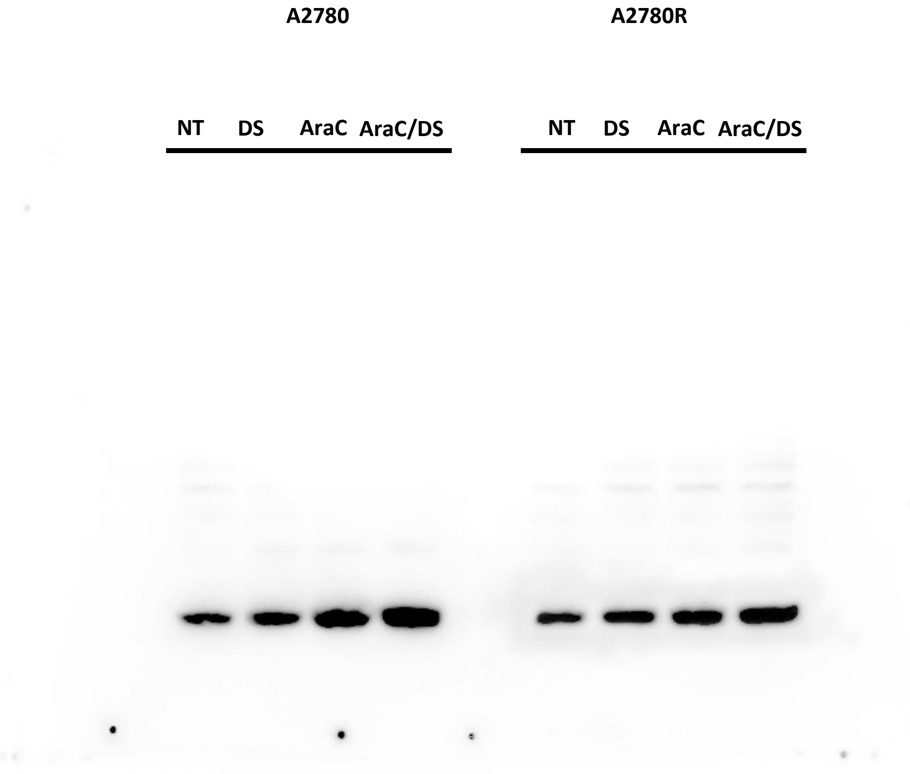

A2780 & A2780R, 24 hours, GAPDH:

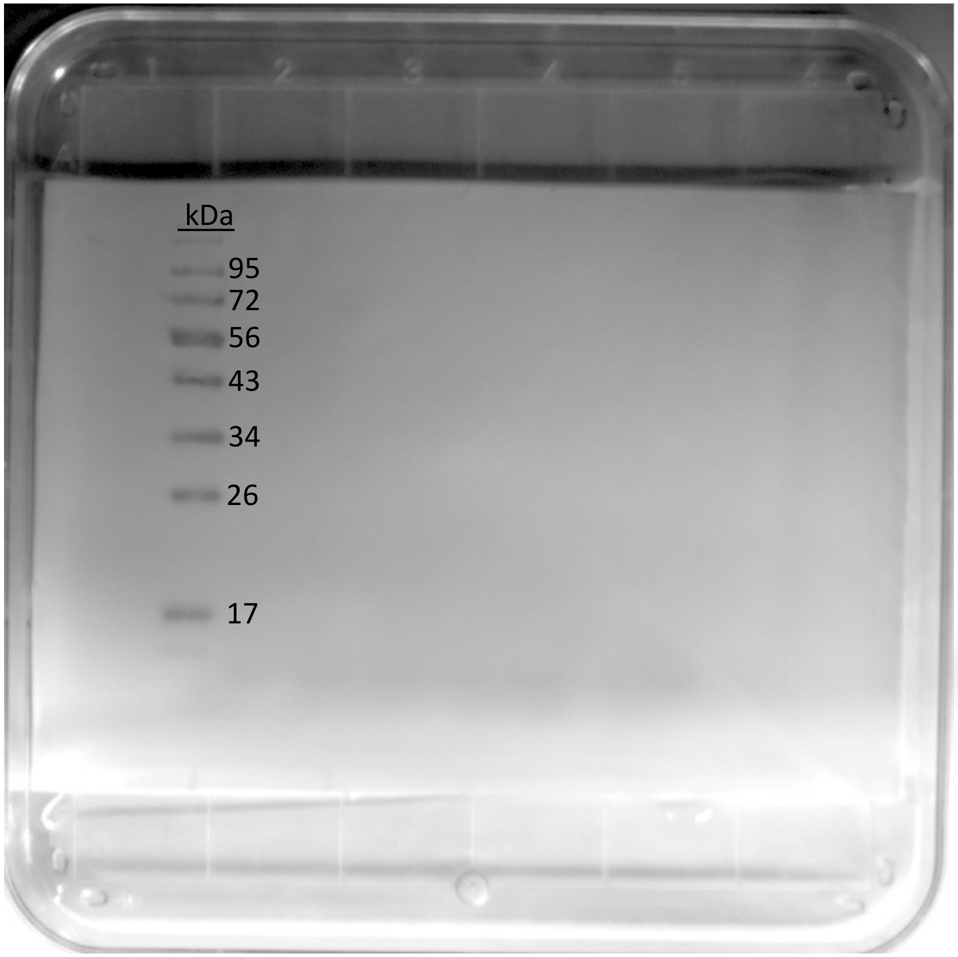

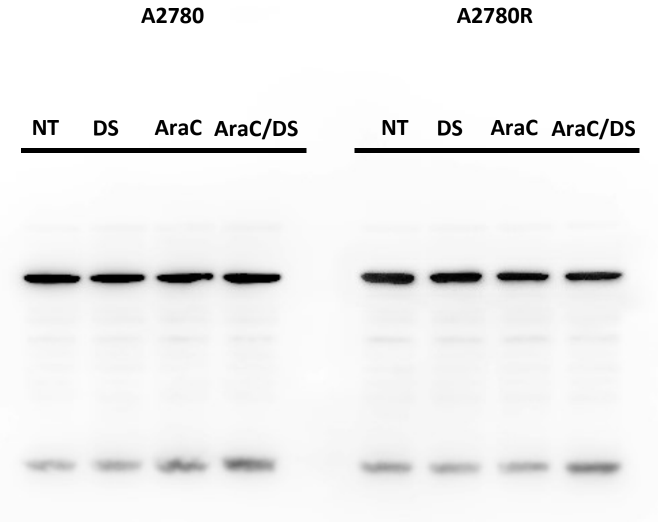

HCT 116 & HT-29, 24 hours, γ-H2AX:

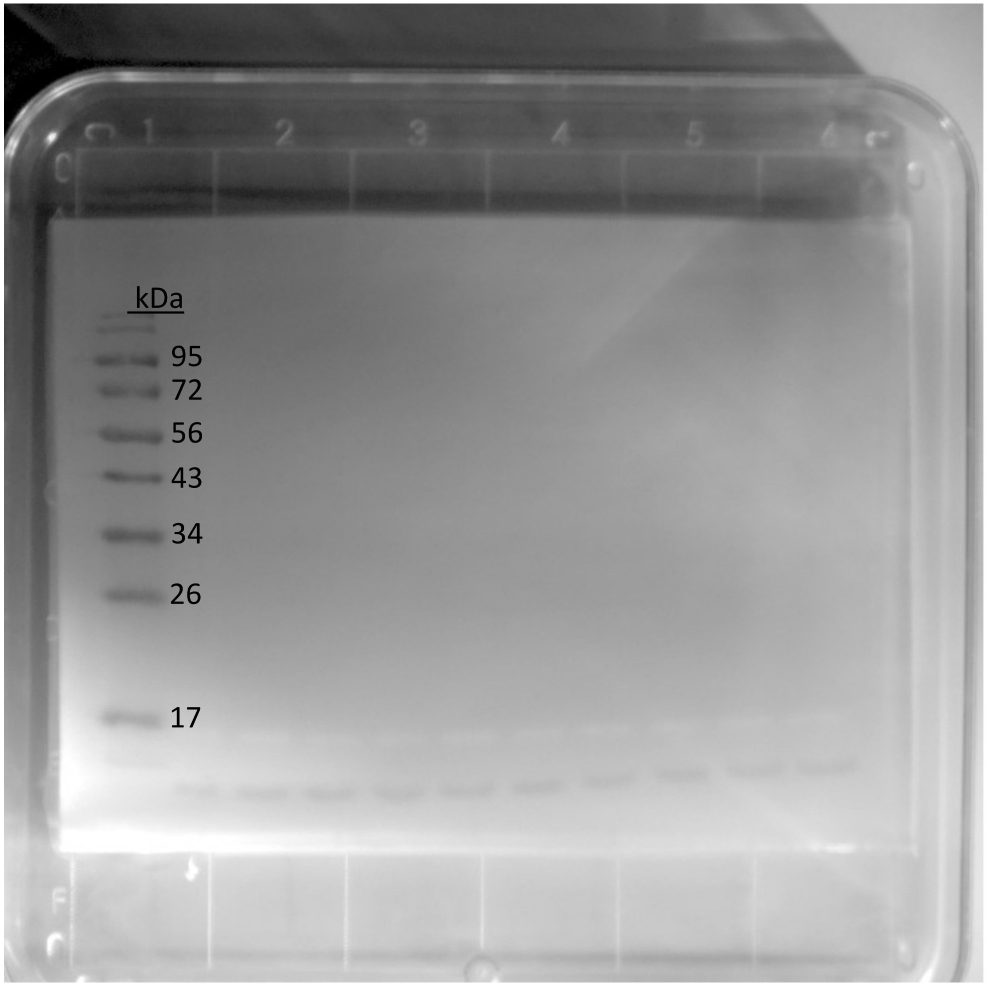

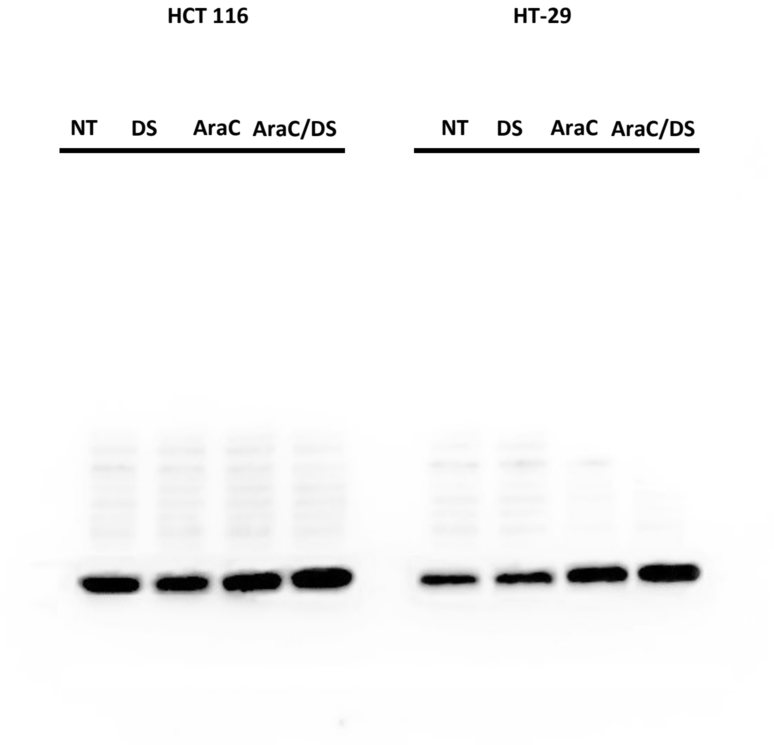

HCT 116 & HT-29, 24 hours, GAPDH:

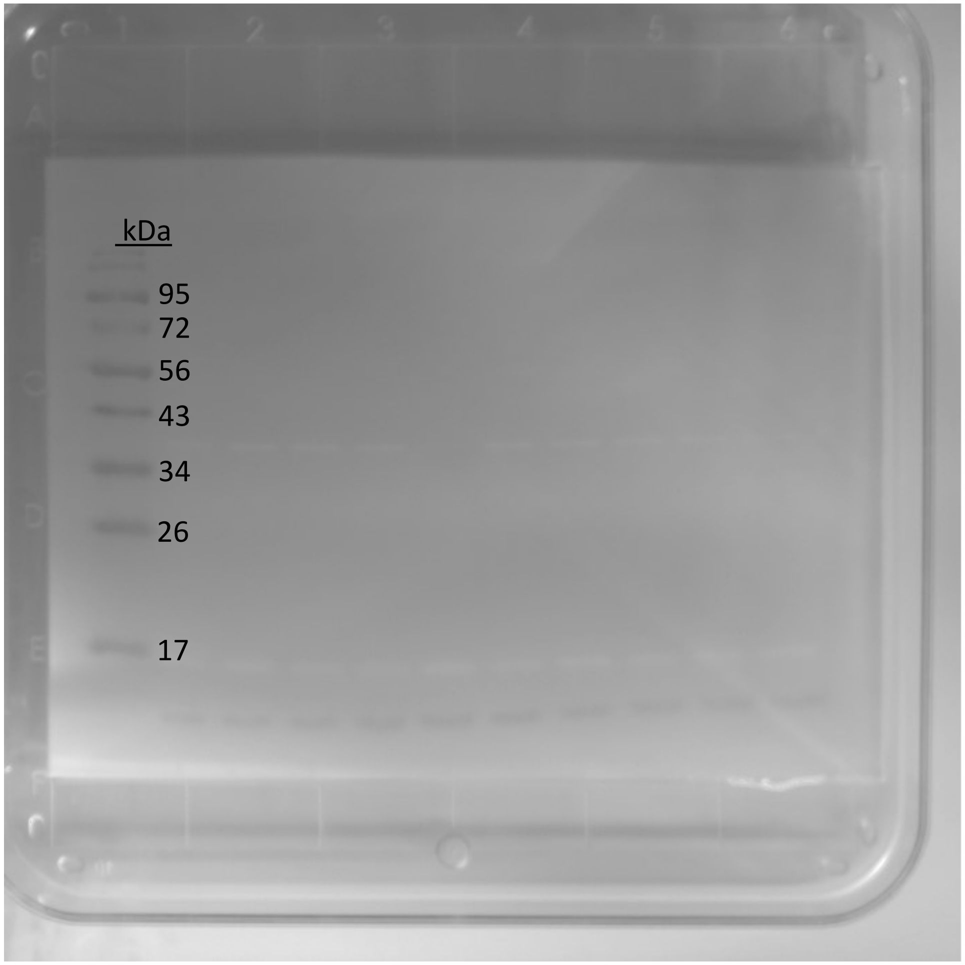

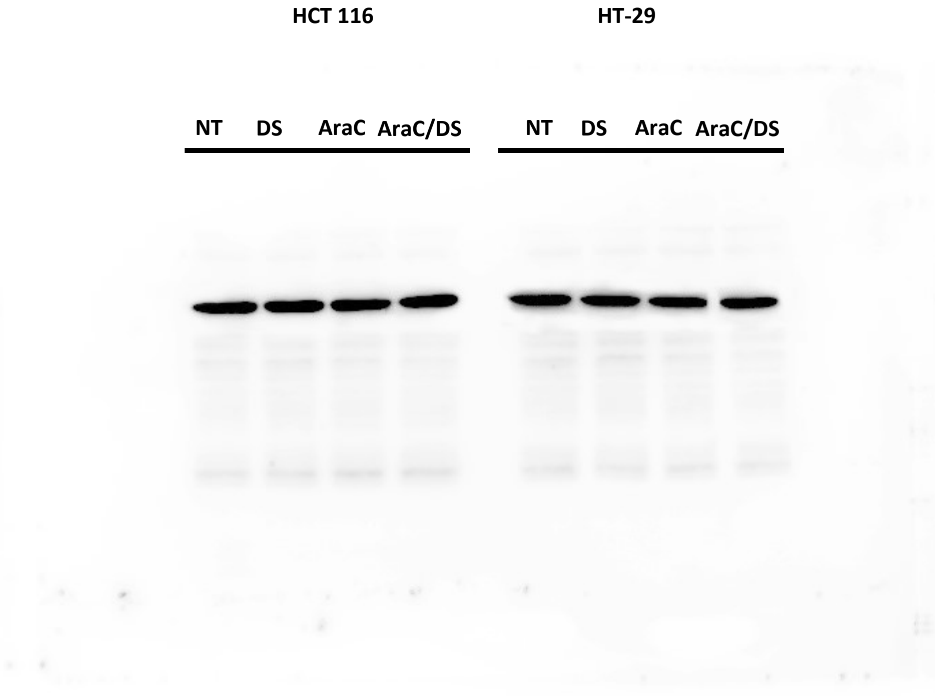

PBMC & THP1-Macrophage, 24 hours, γ-H2AX:

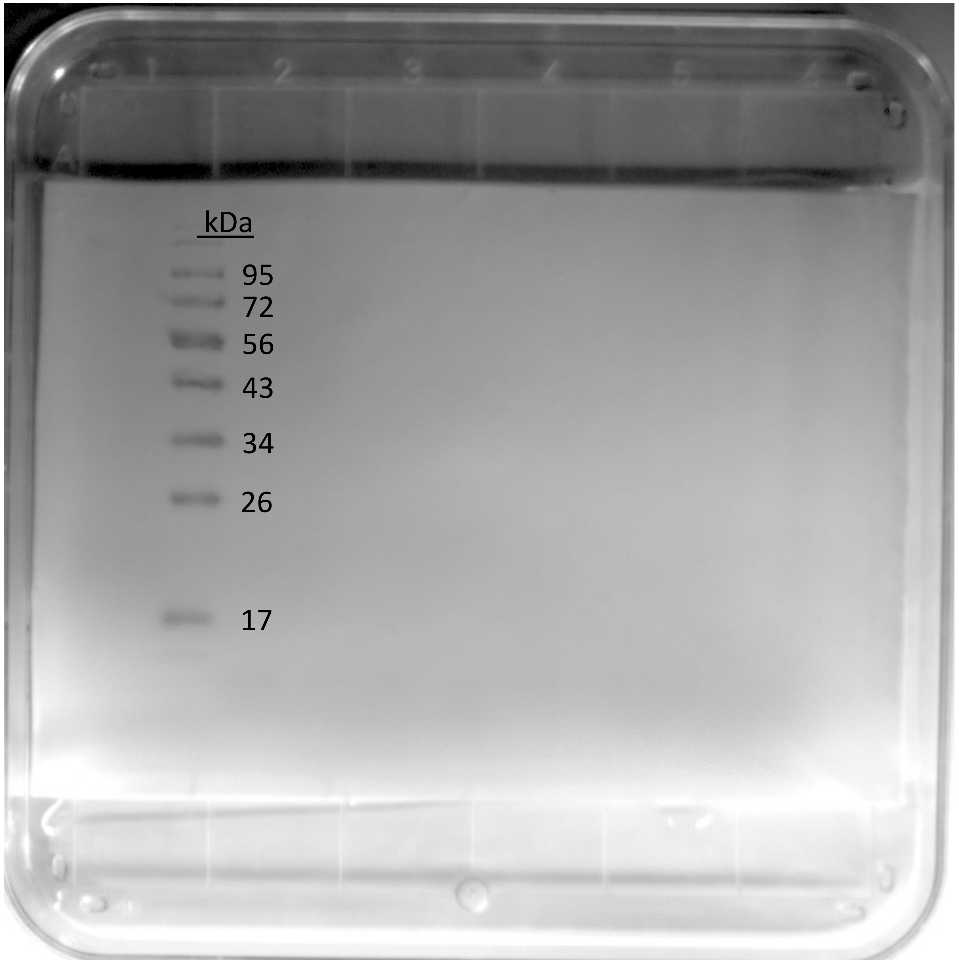

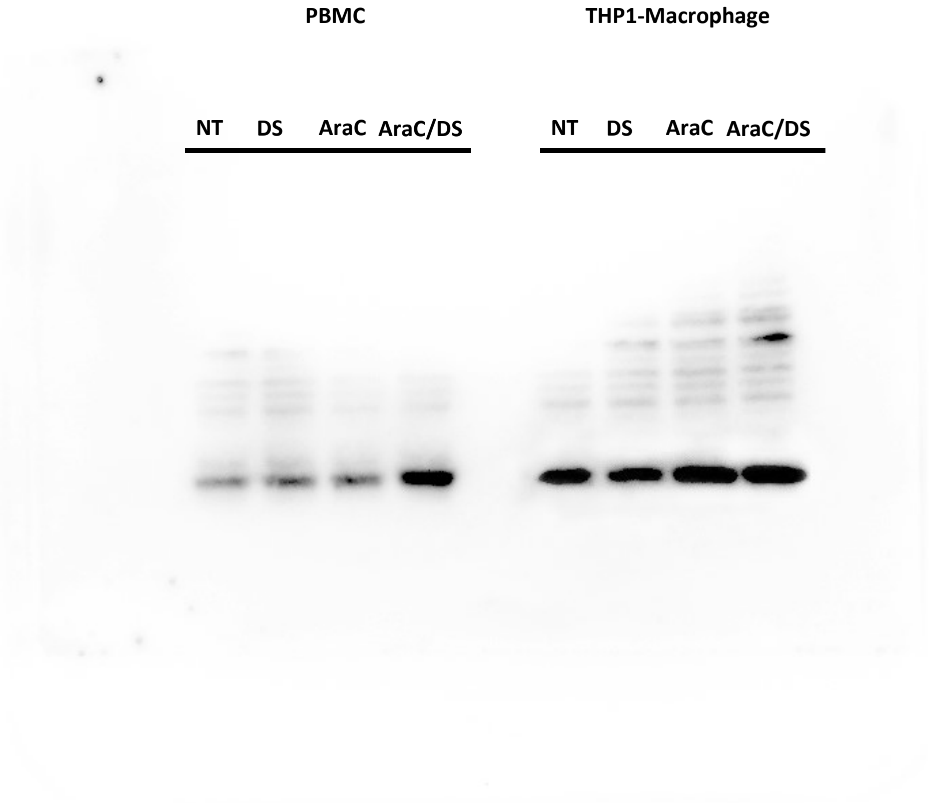

PBMC & THP1-Macrophage, 24 hours, GAPDH:

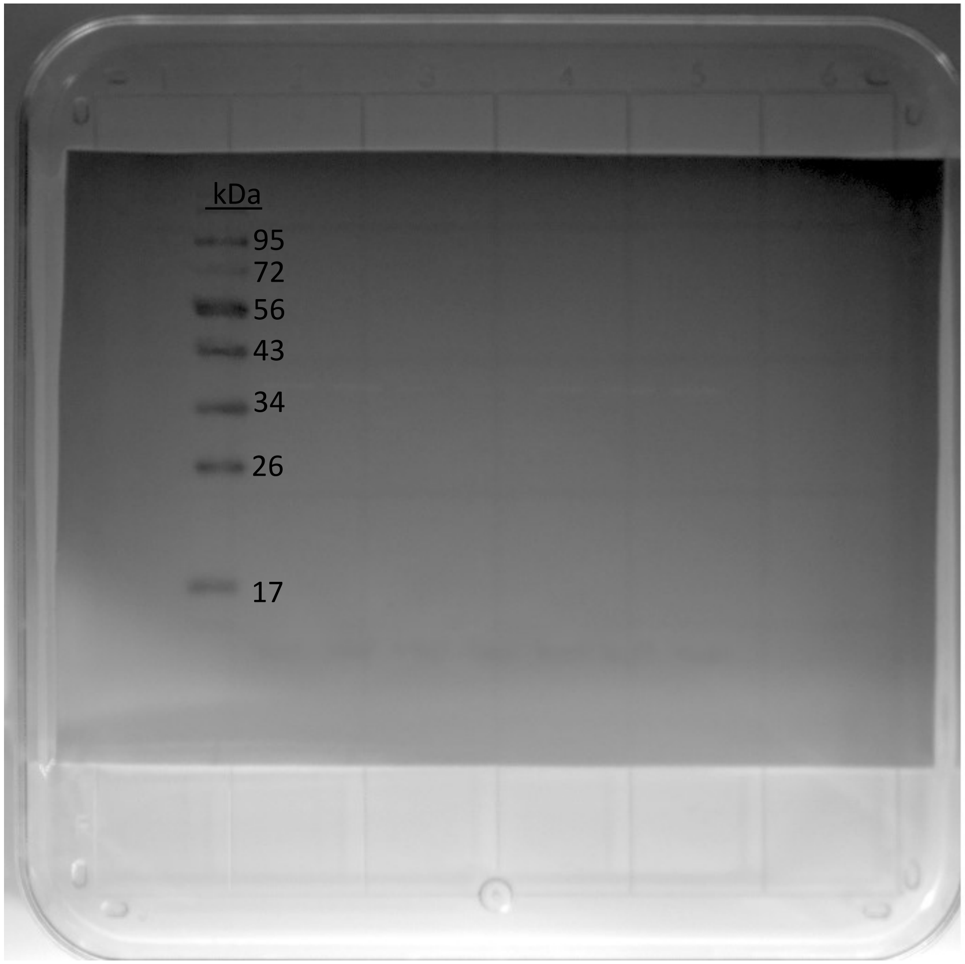

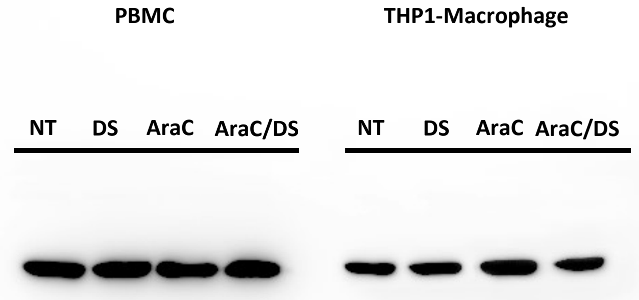

Figure 2 Raw Western Blot Images

A2780, 6 hours, P-TBK1:

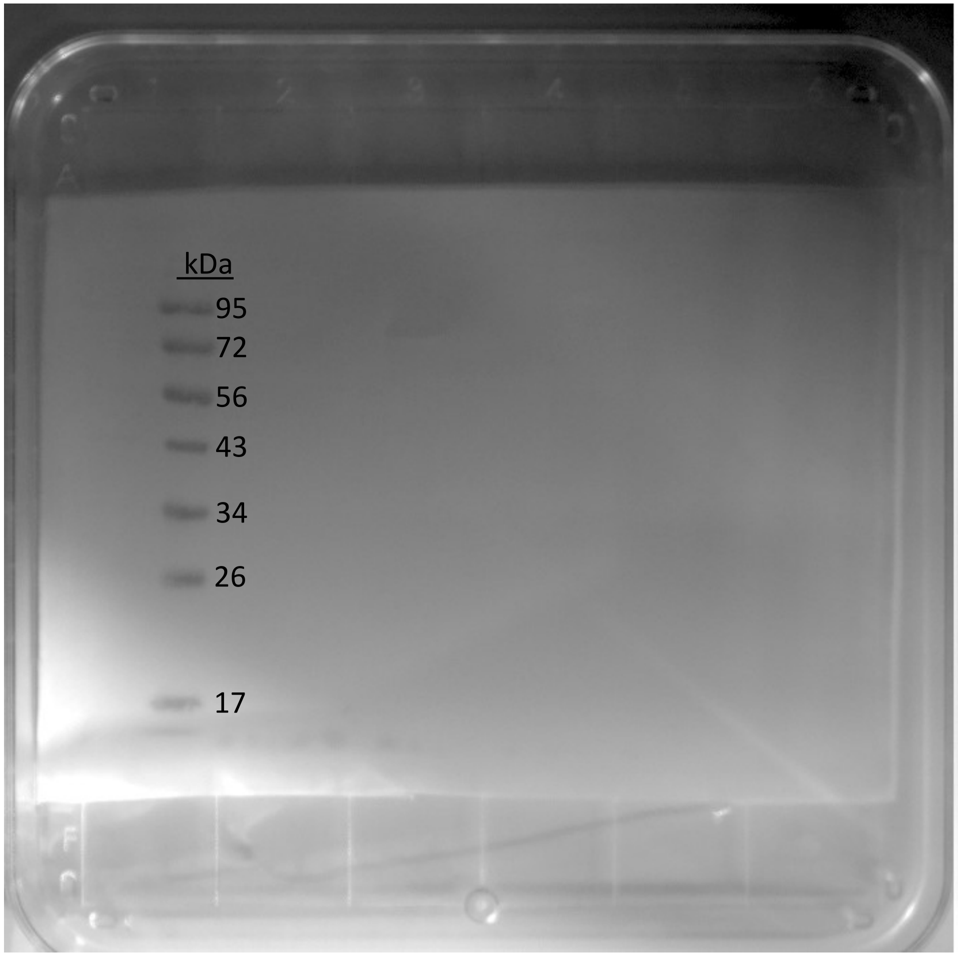

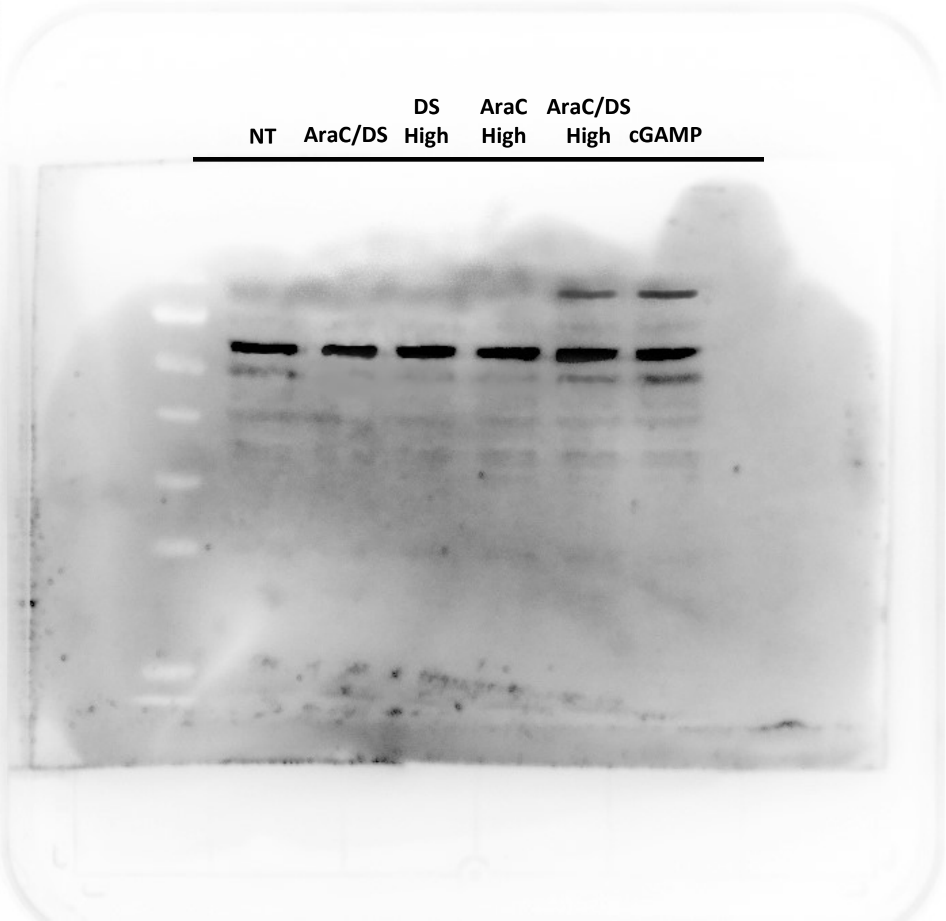

A2780, 6 hours, P-IRF3:

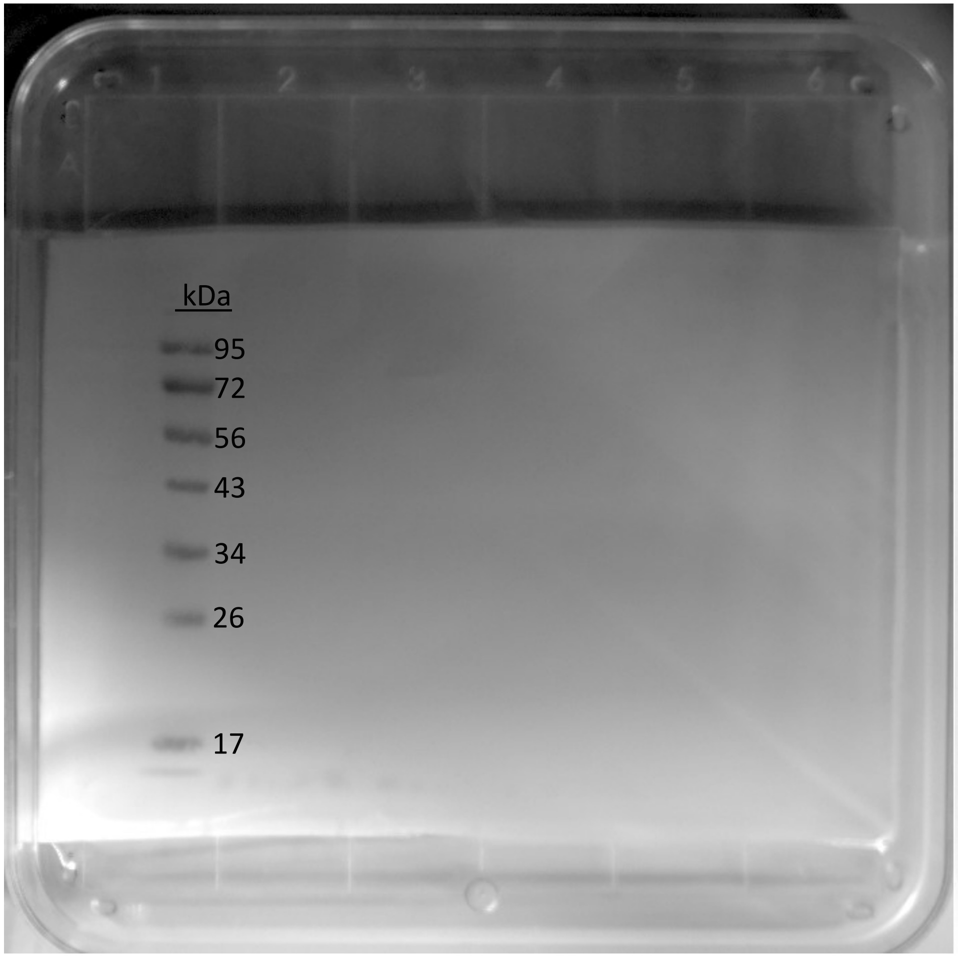

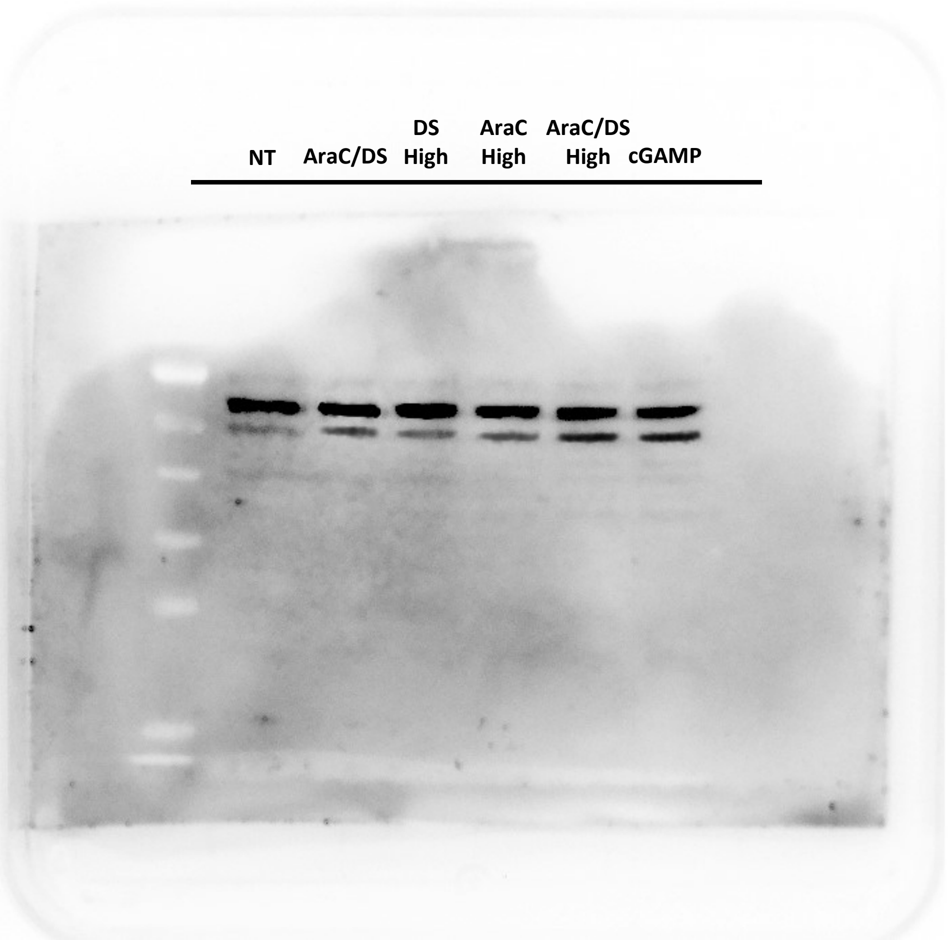

A2780, 6 hours, P-STING:

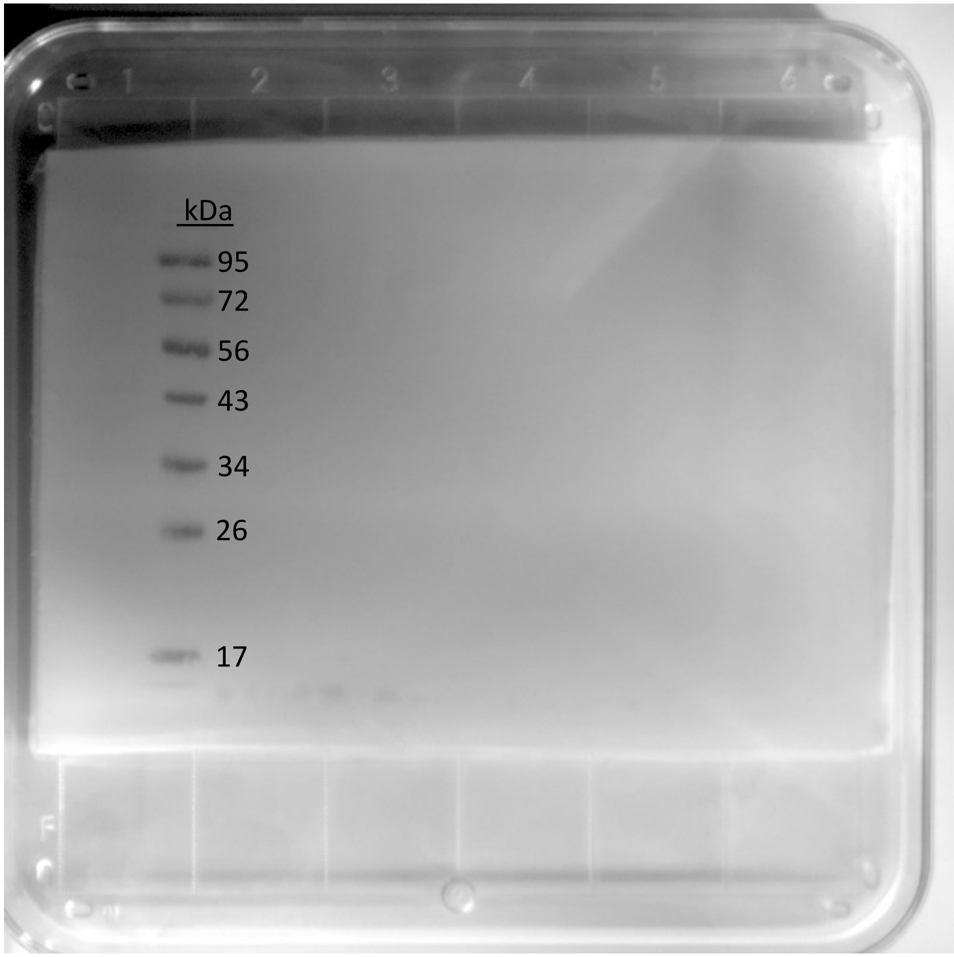

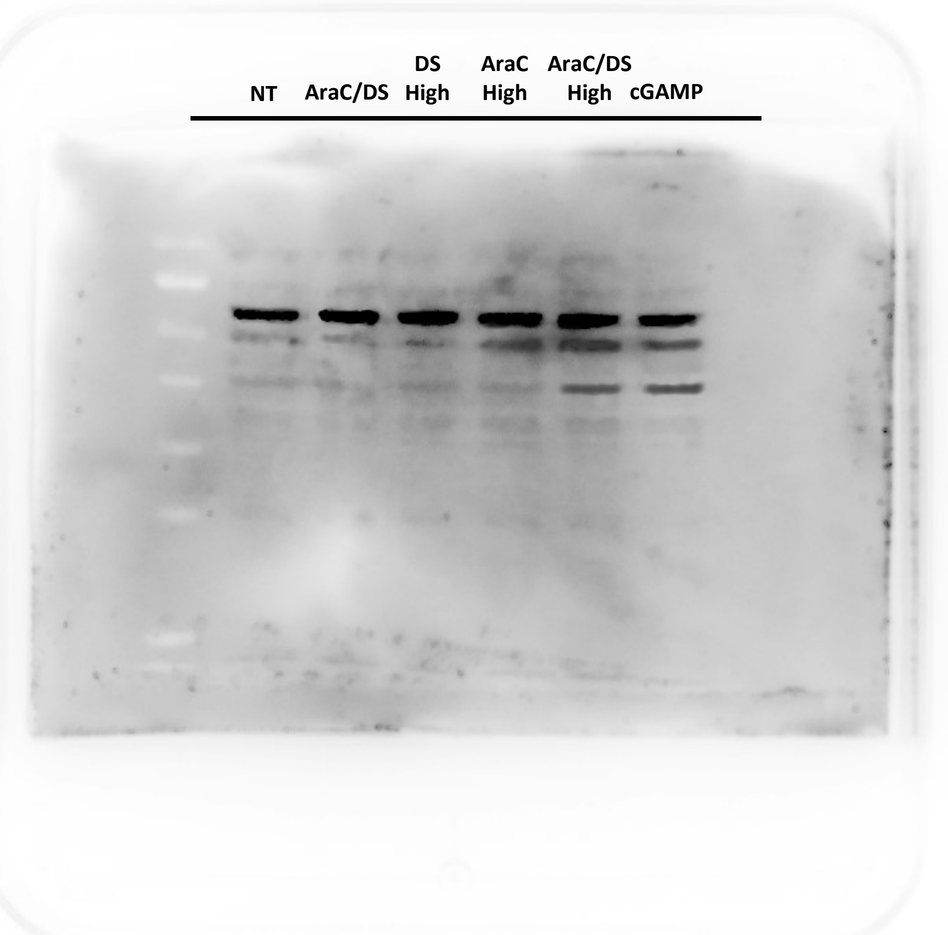

A2780, 6 hours, GAPDH:

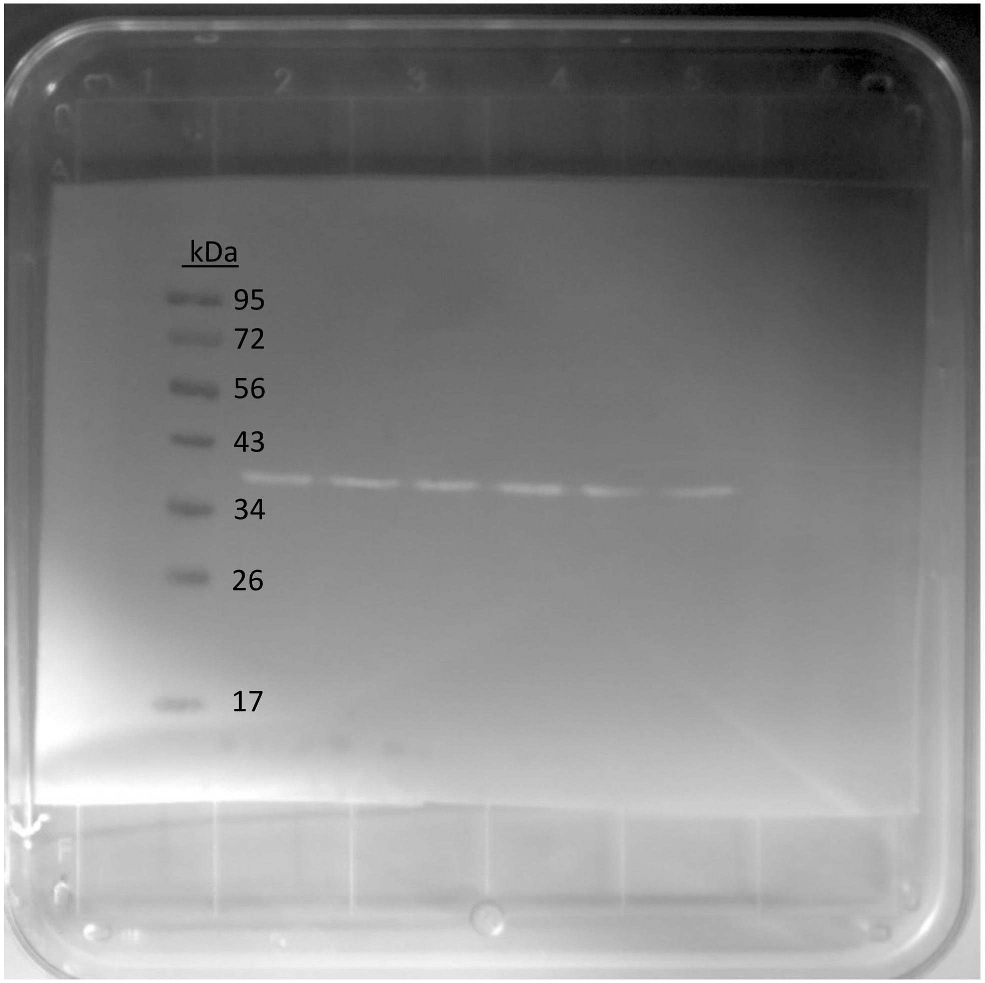

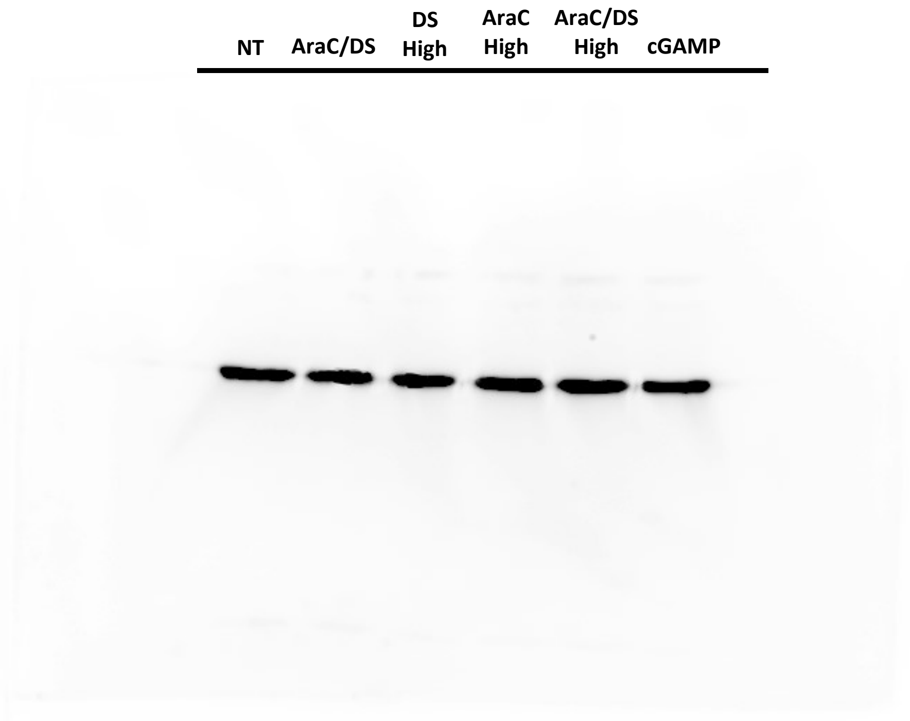

A2780R, 6 hours, P-TBK1:

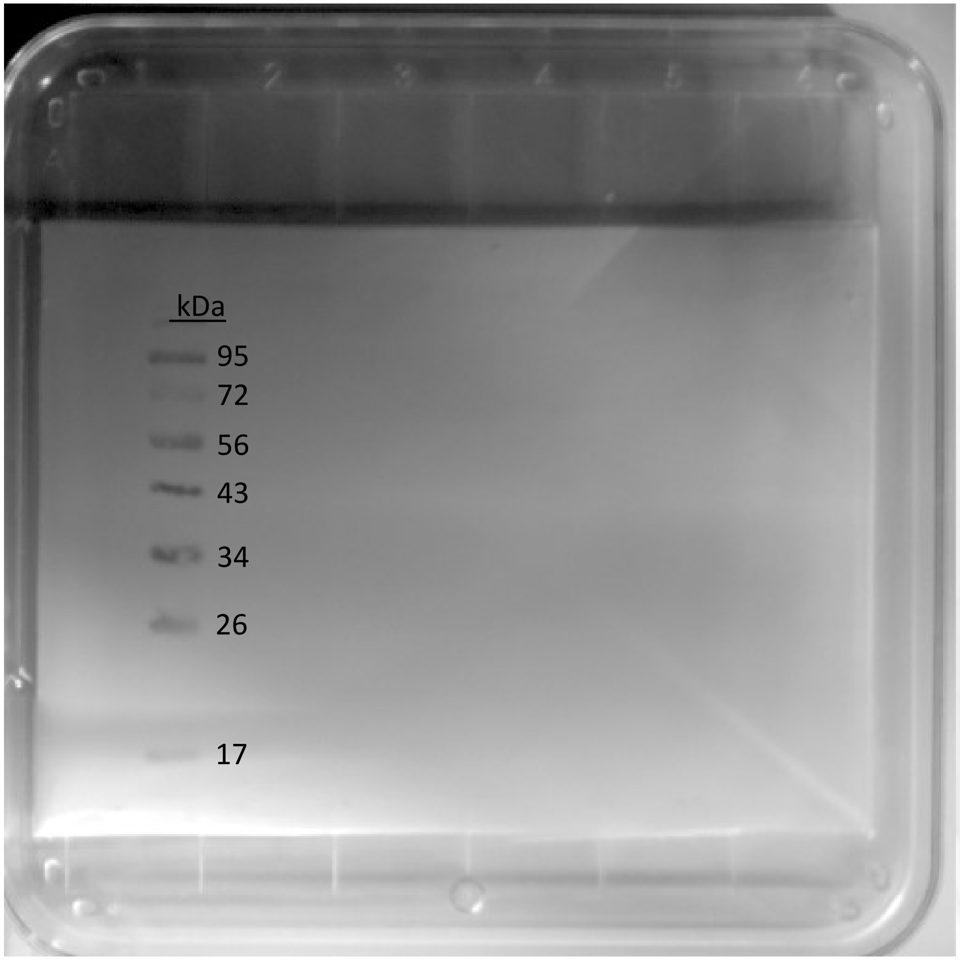

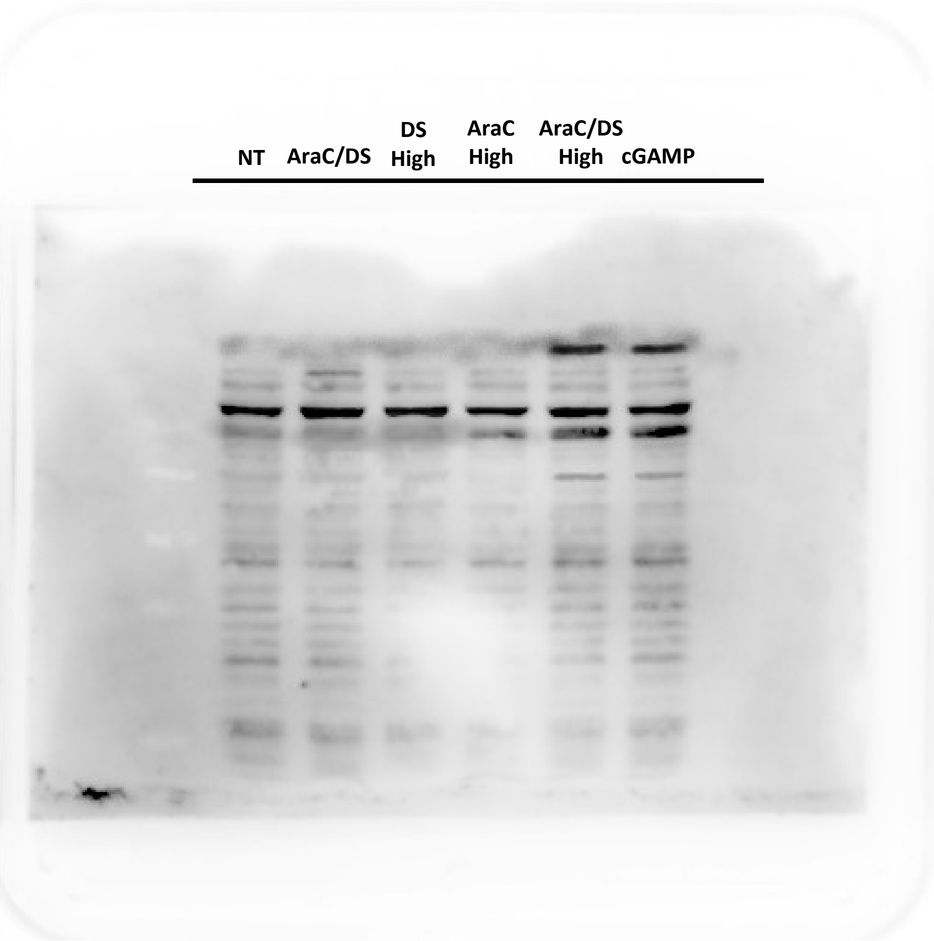

A2780R, 6 hours, P-IRF3:

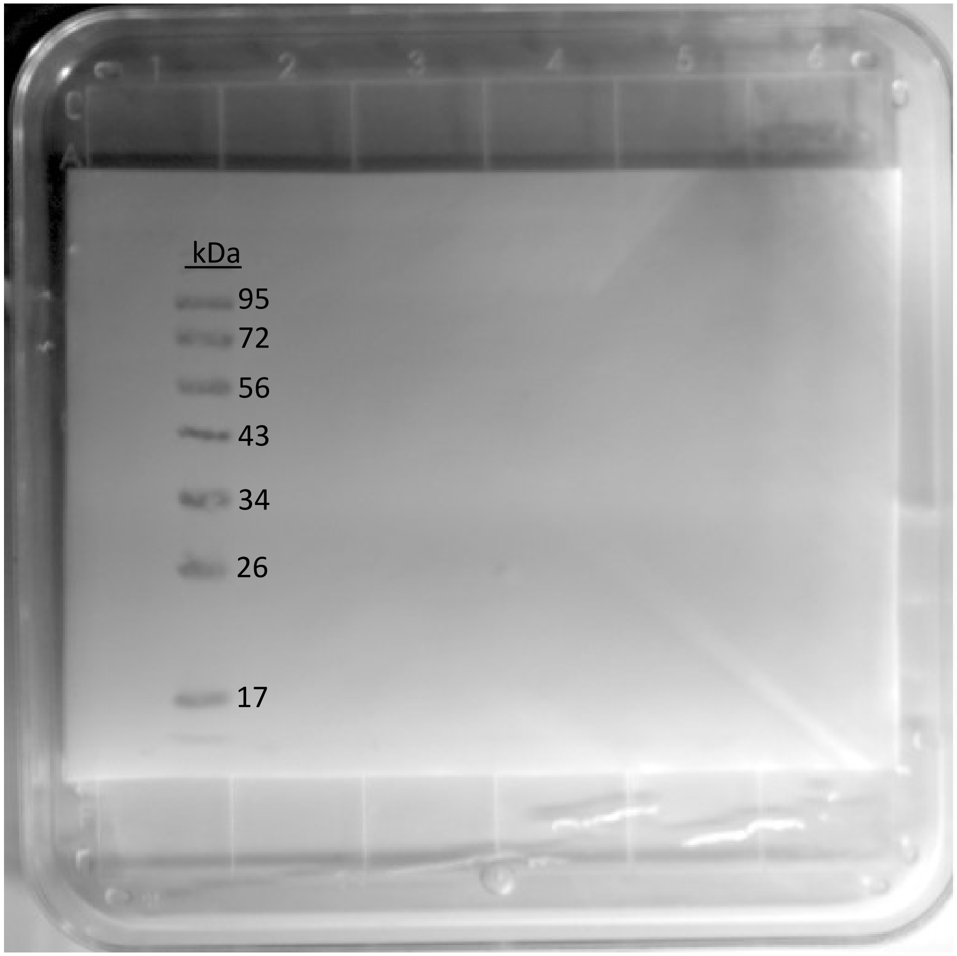

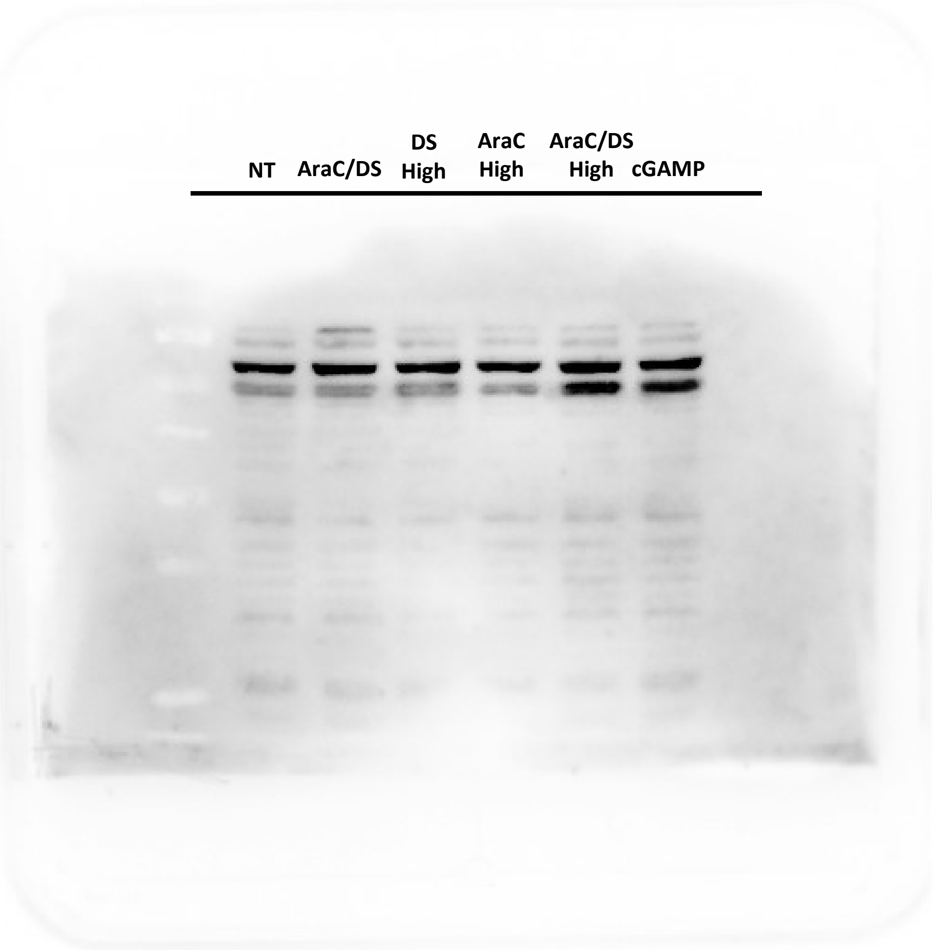

A2780R, 6 hours, P-STING:

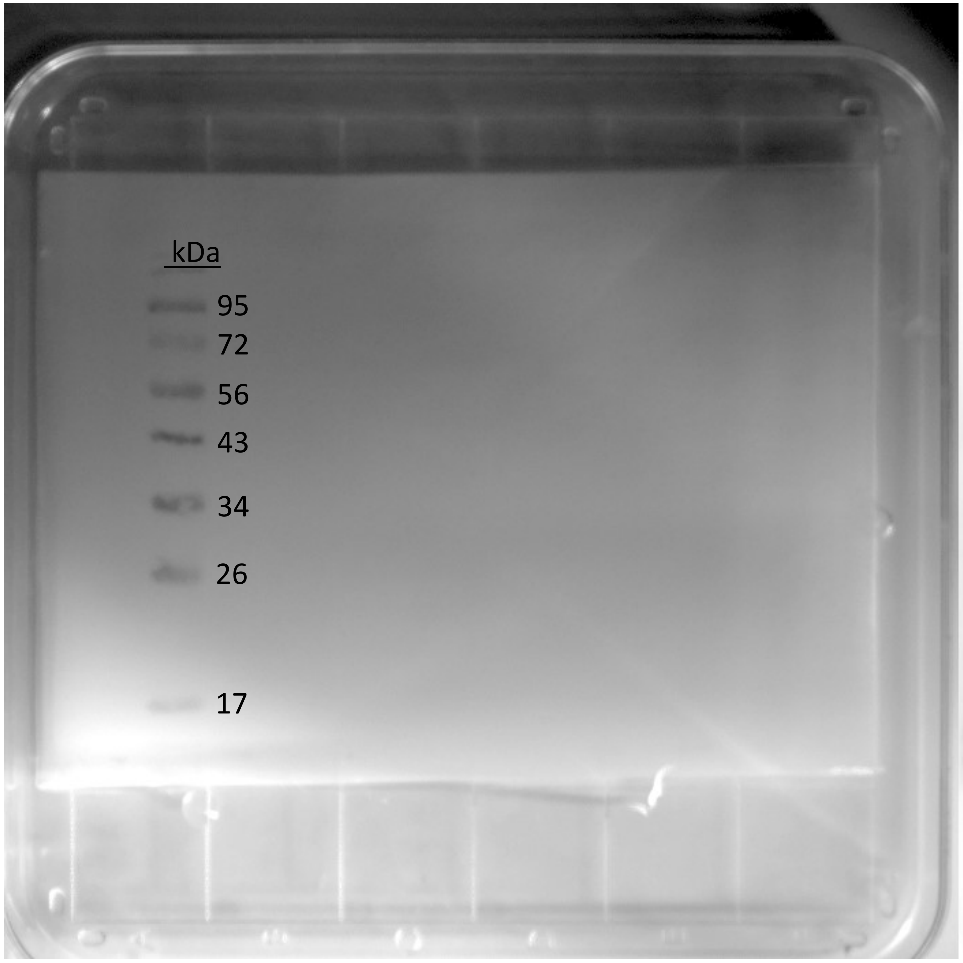

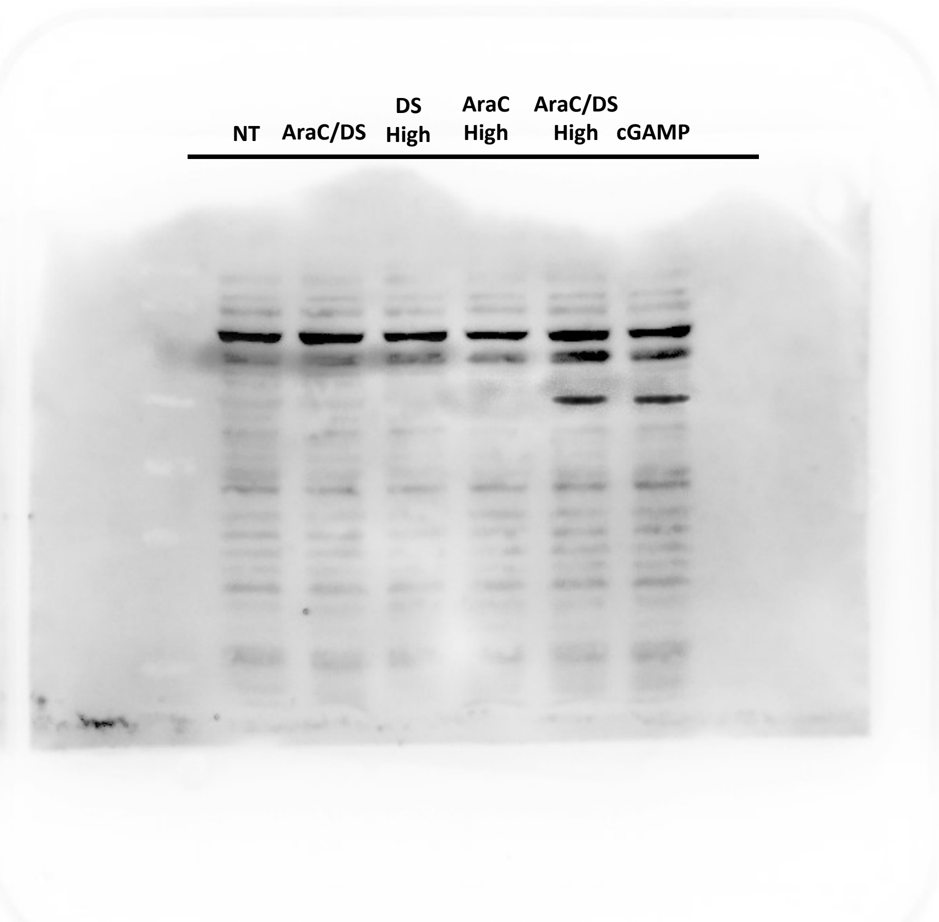

A2780R, 6 hours, GAPDH:

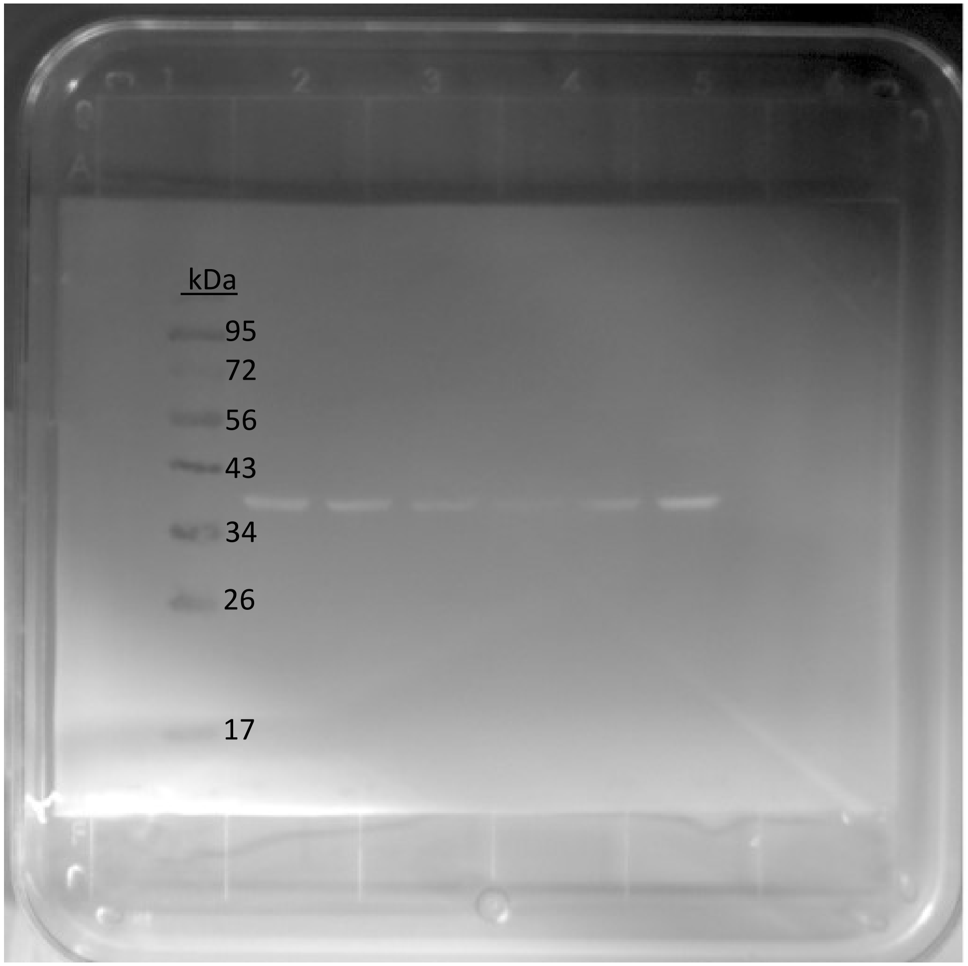

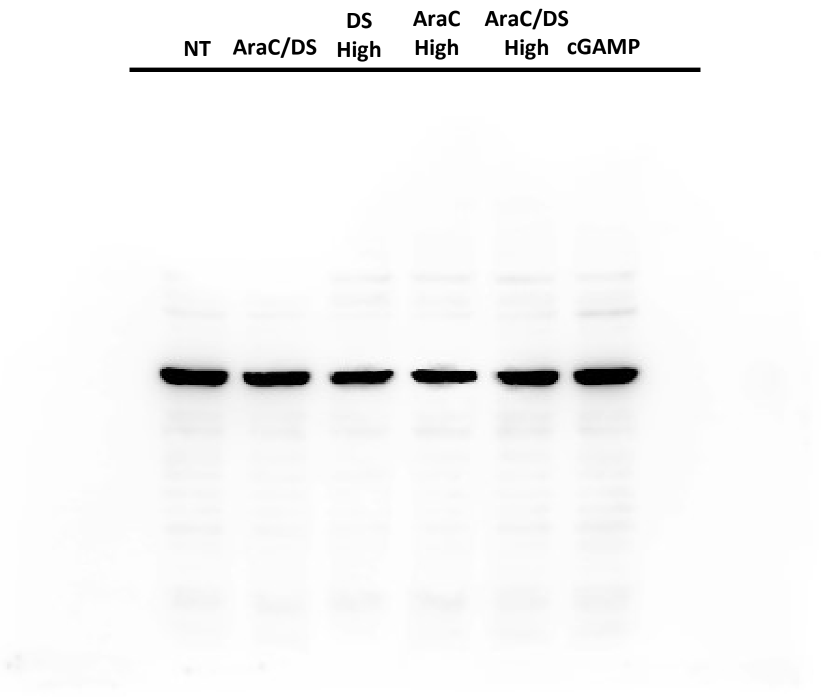

HCT 116, 6 hours, P-TBK1:

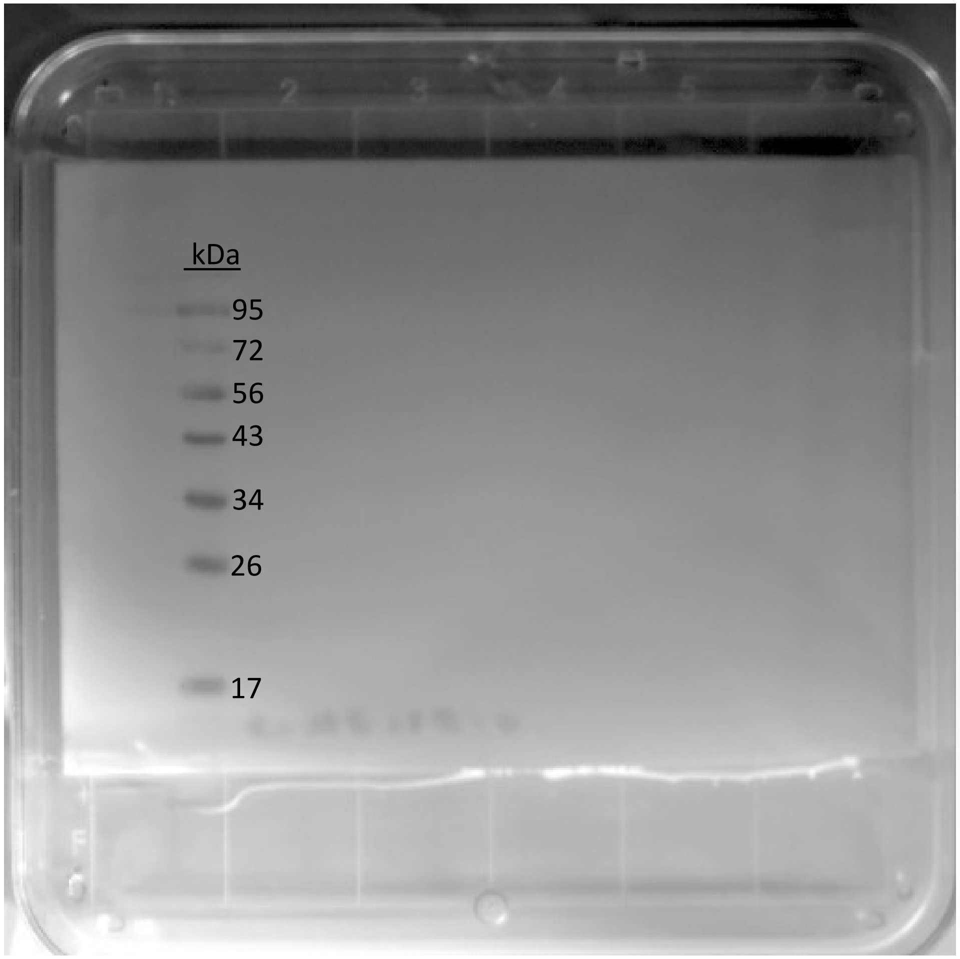

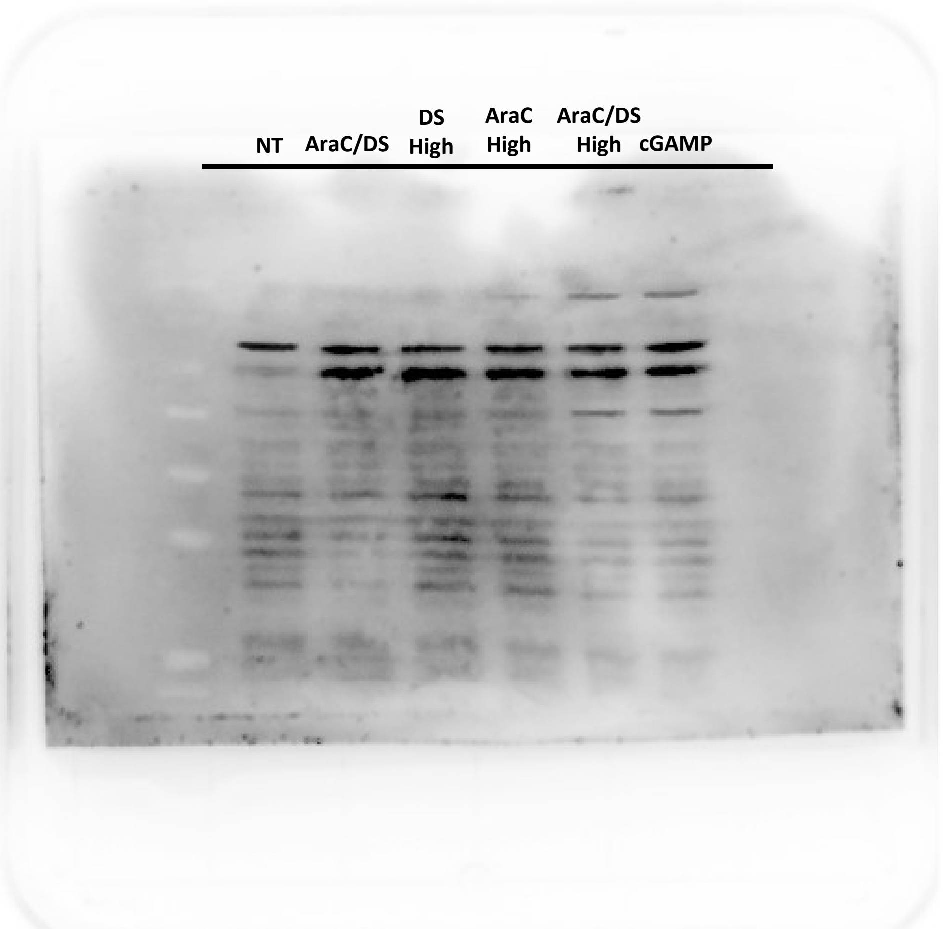

HCT 116, 6 hours, P-IRF3:

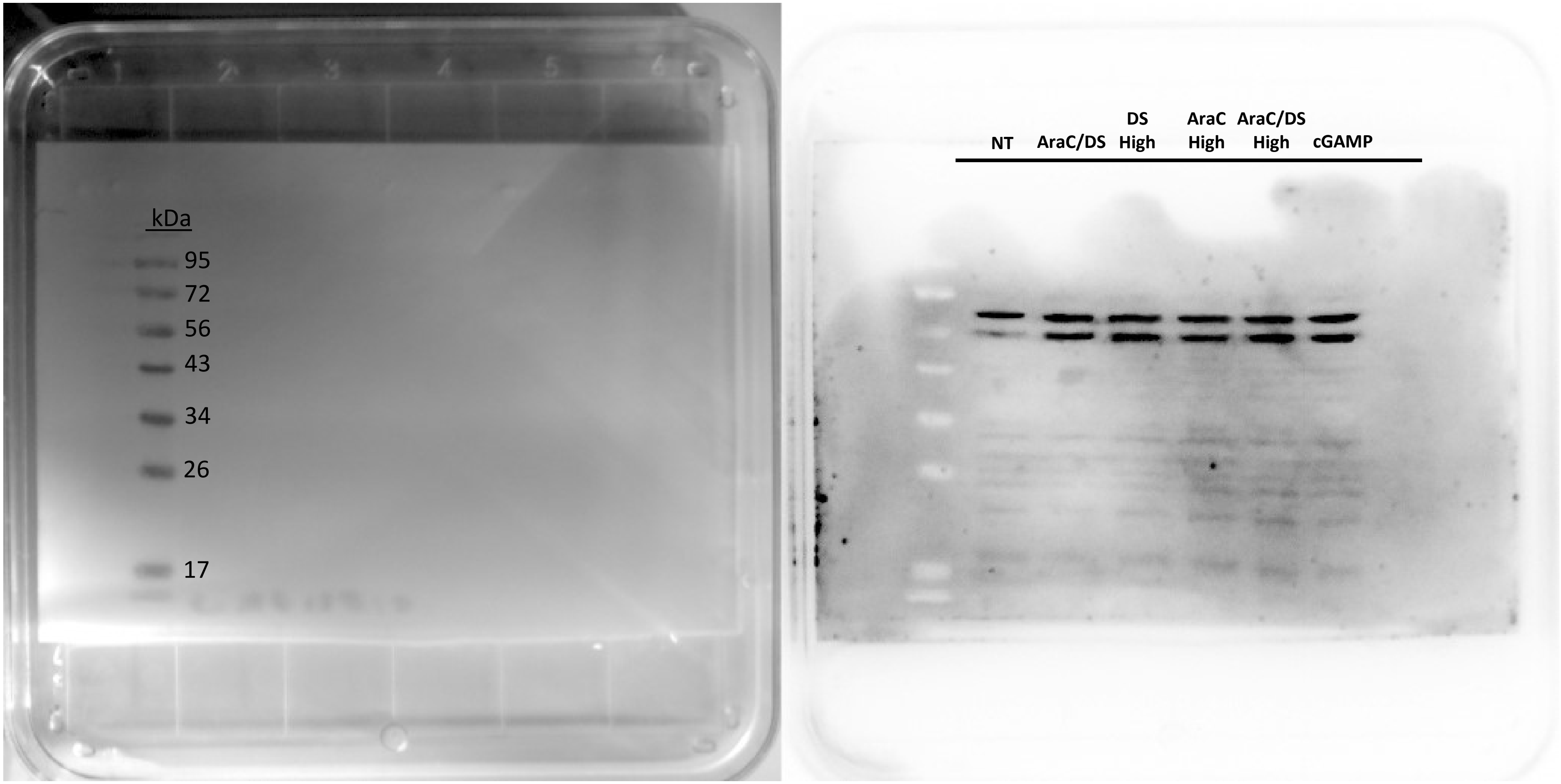

HCT 116, 6 hours, P-STING:

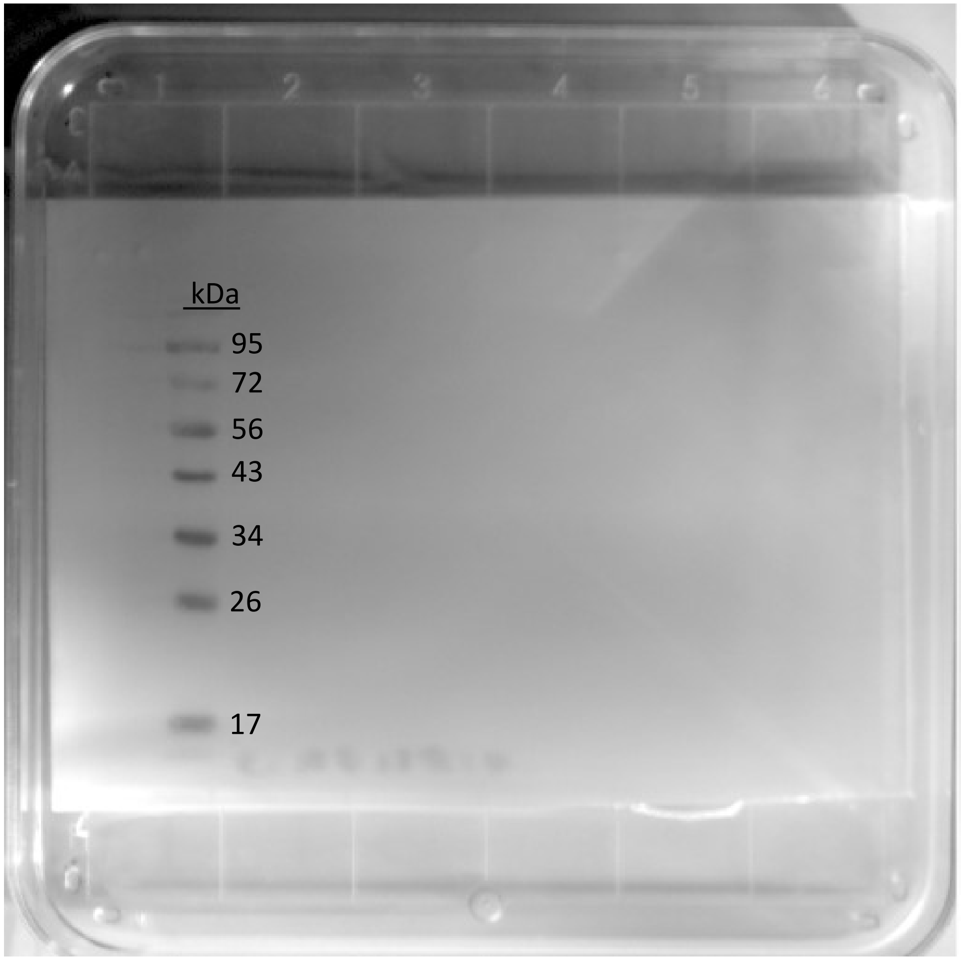

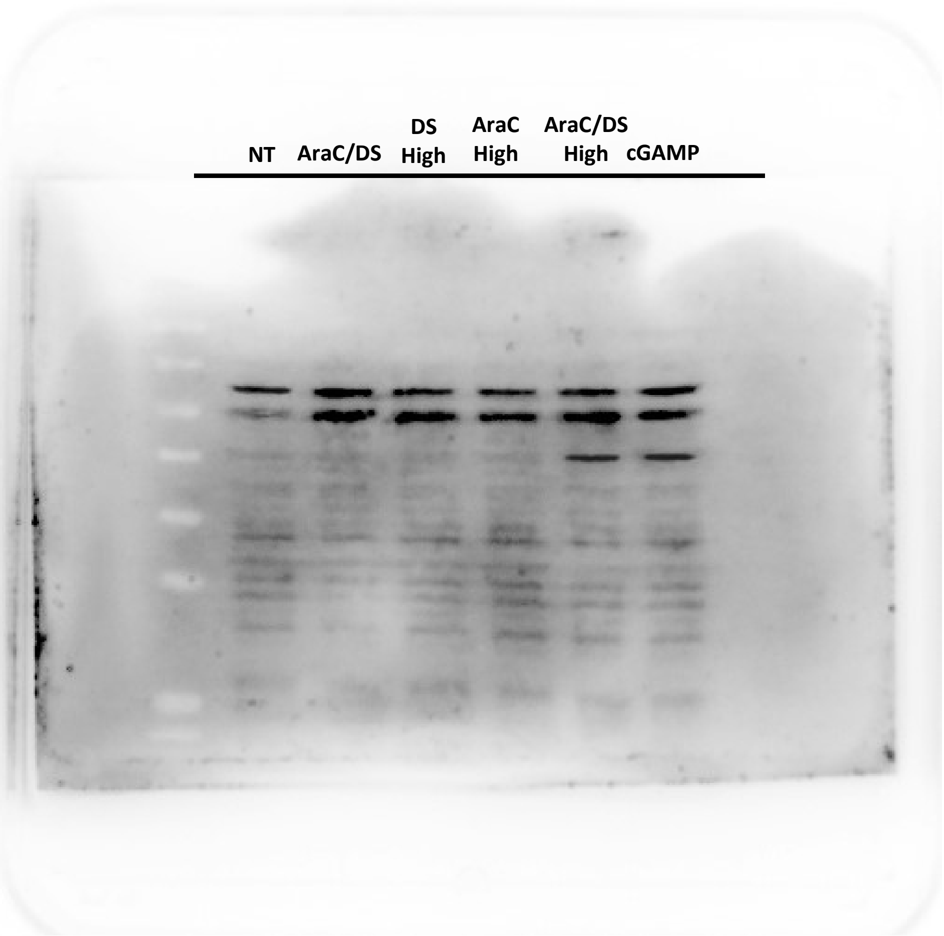

HCT 116, 6 hours, GAPDH:

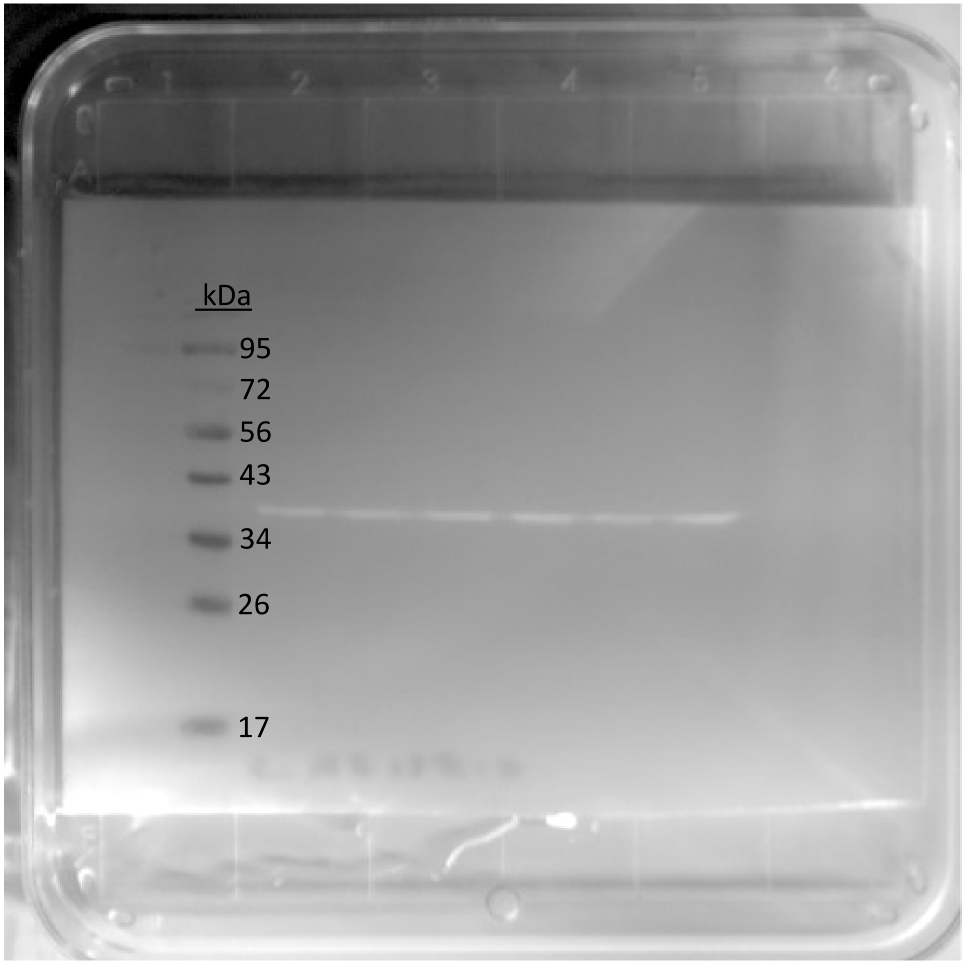

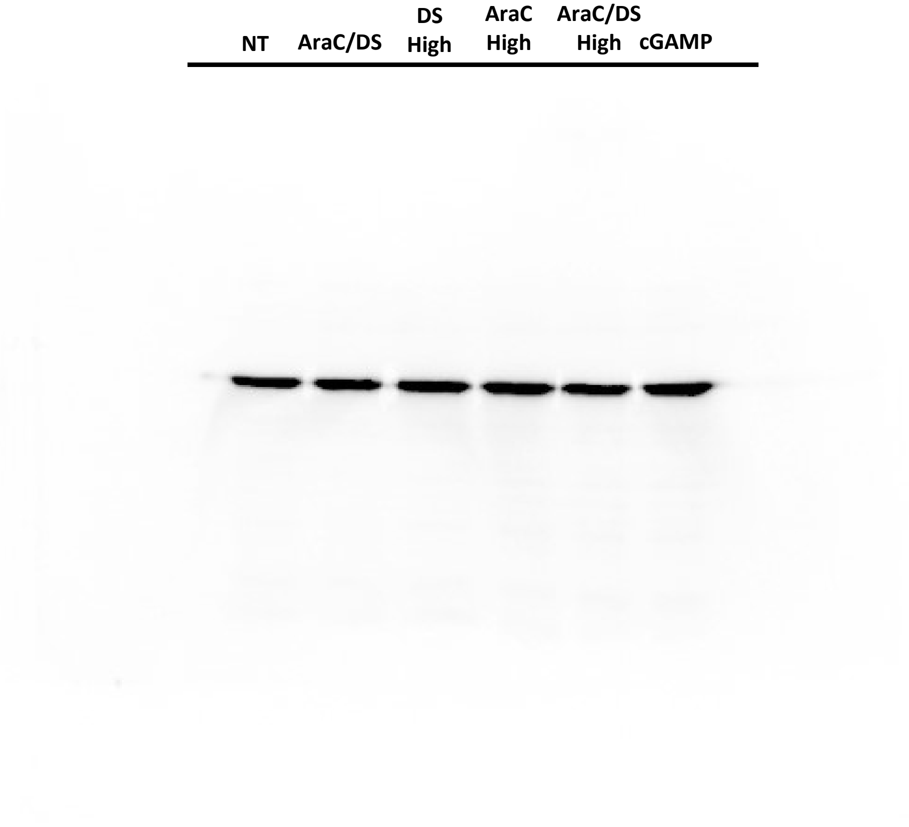

HT-29, 6 hours, P-TBK1:

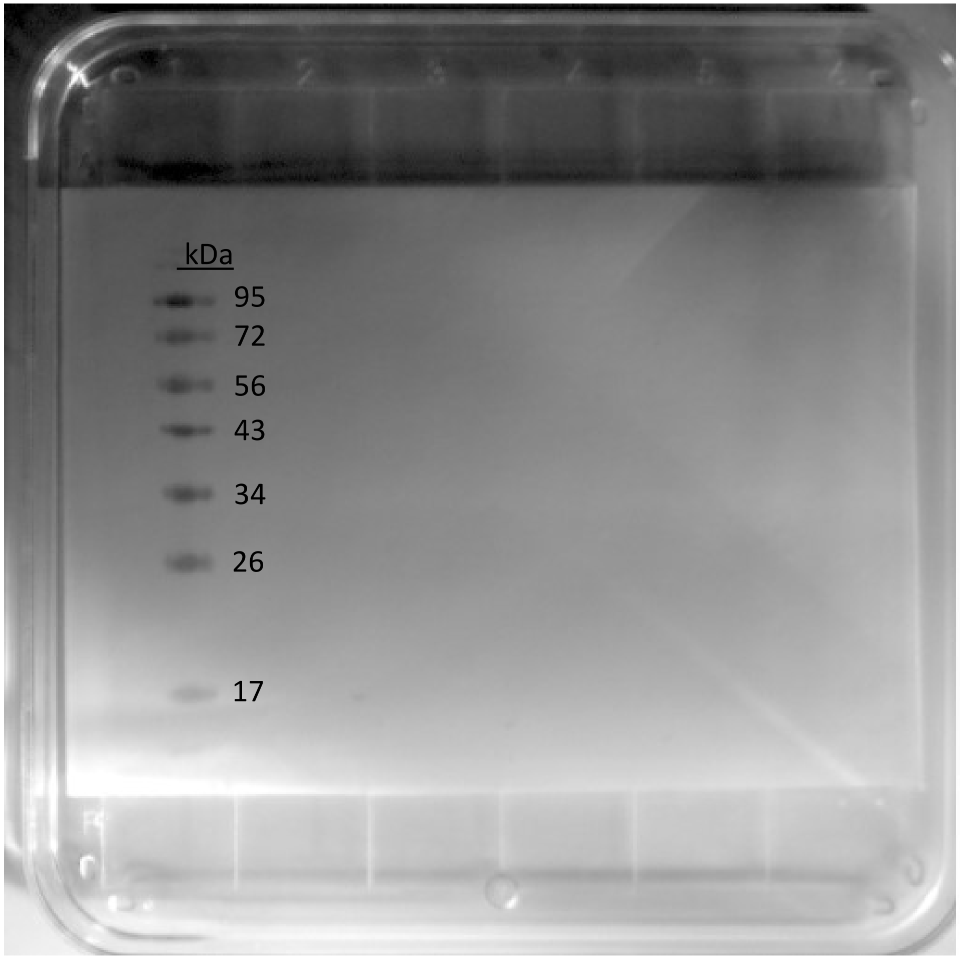

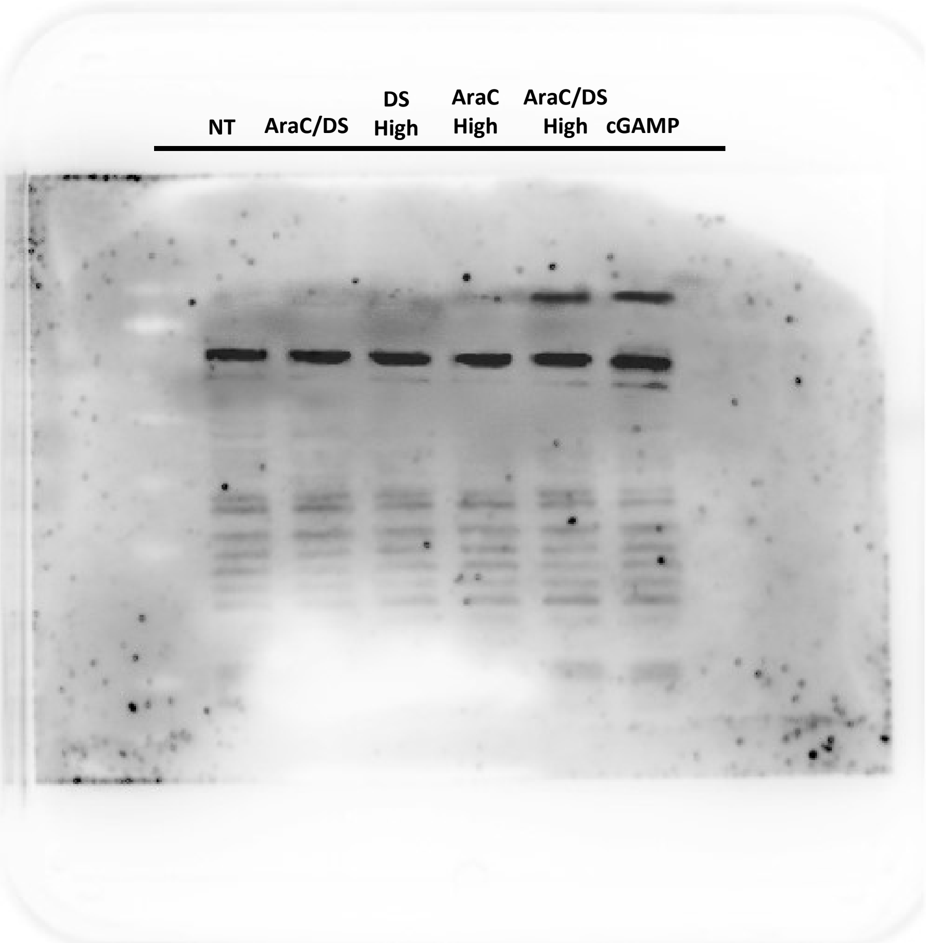

HT-29, 6 hours, P-IRF3:

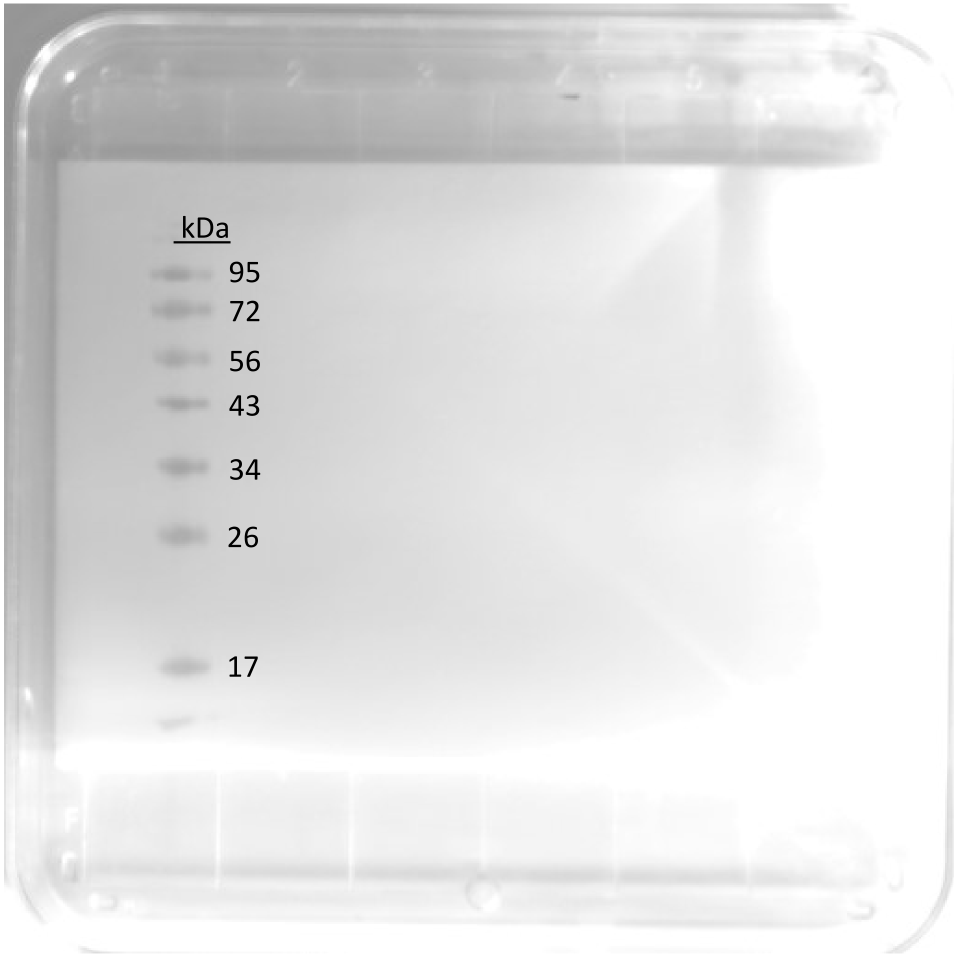

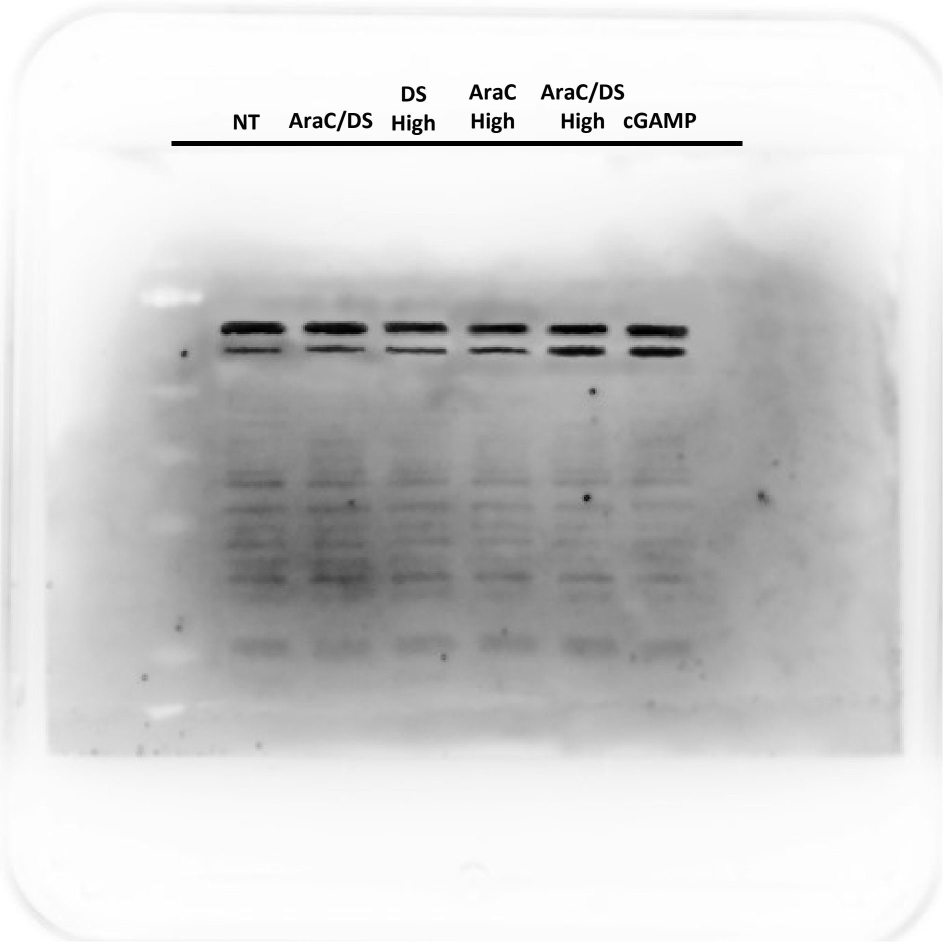

HT-29, 6 hours, P-STING:

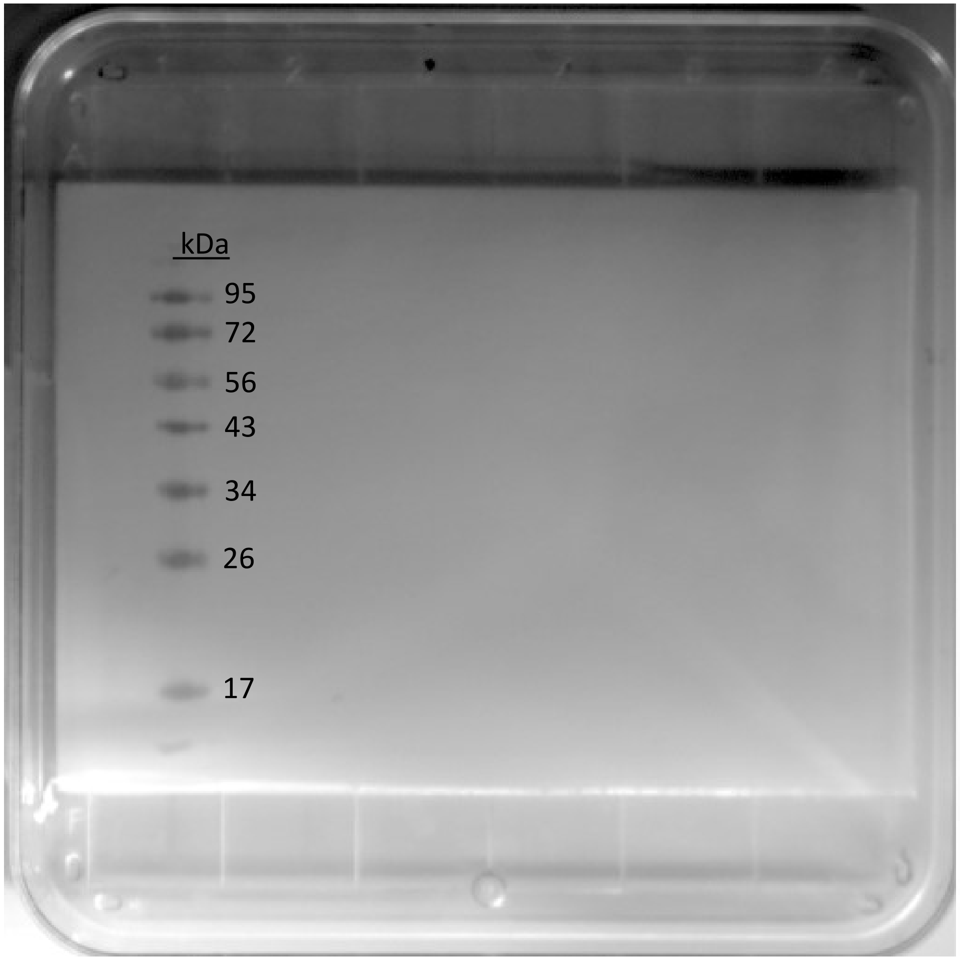

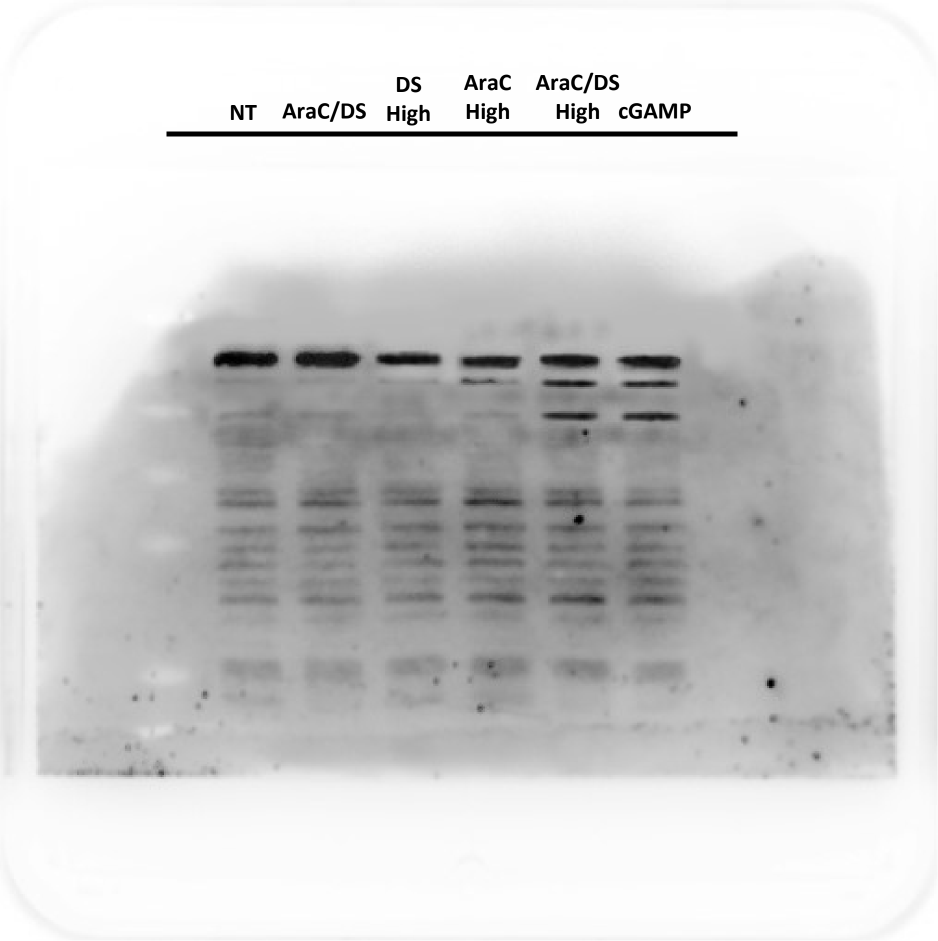

HT-29, 6 hours, GAPDH:

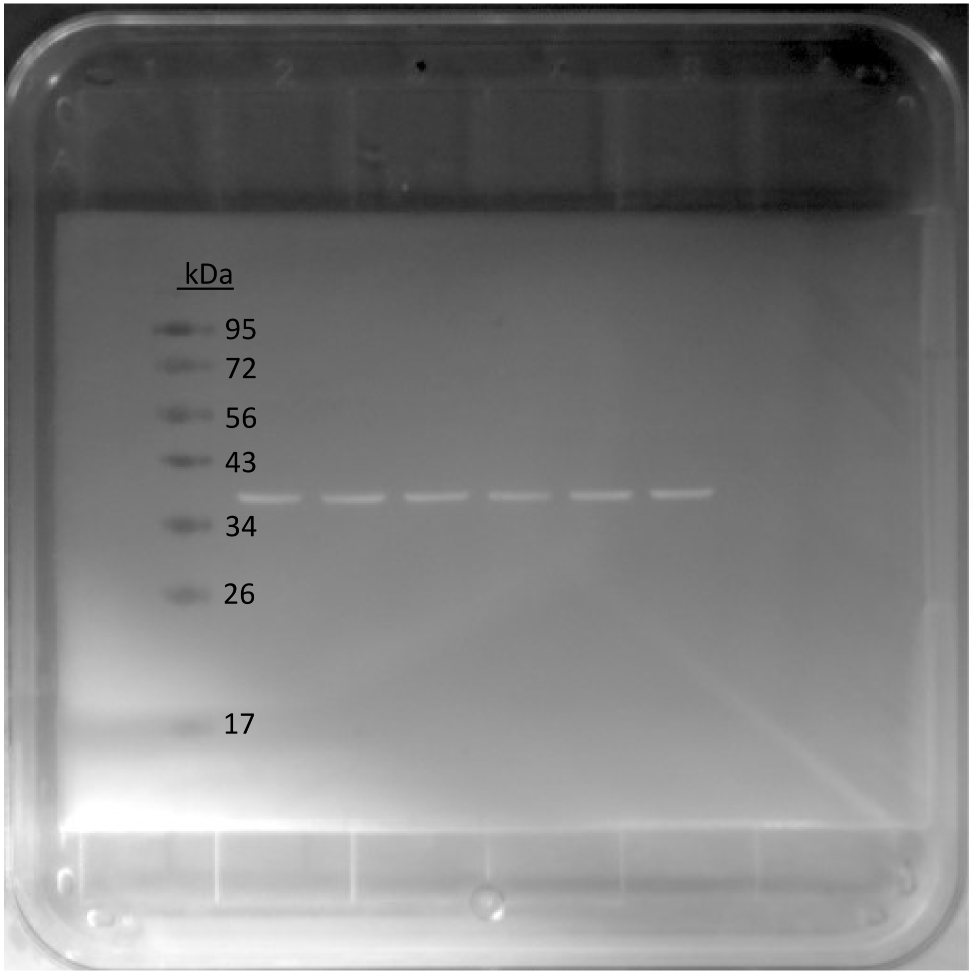

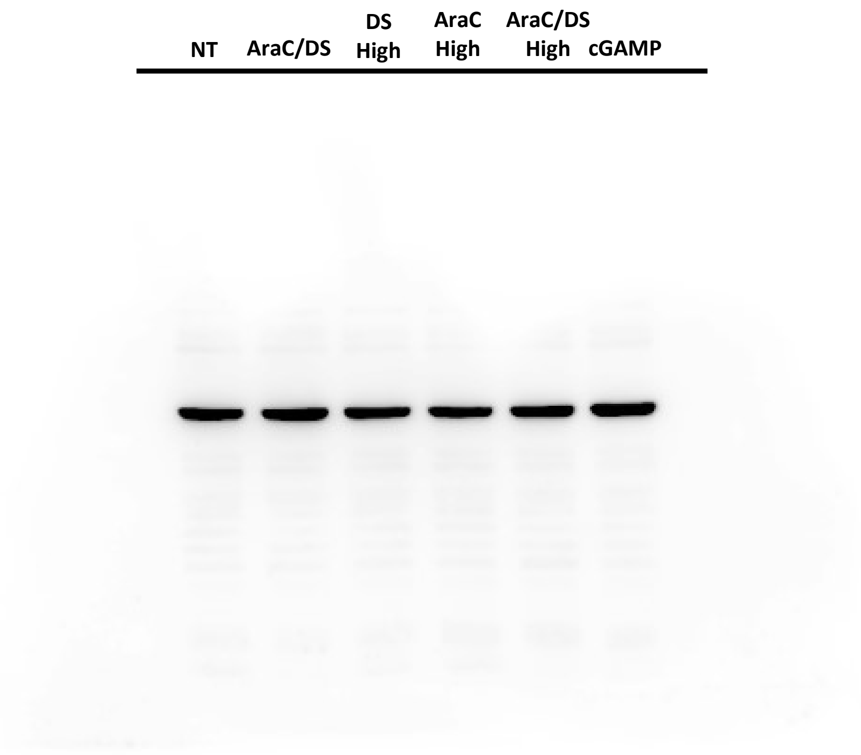

THP1-Macrophage, 6 hours, P-TBK1:

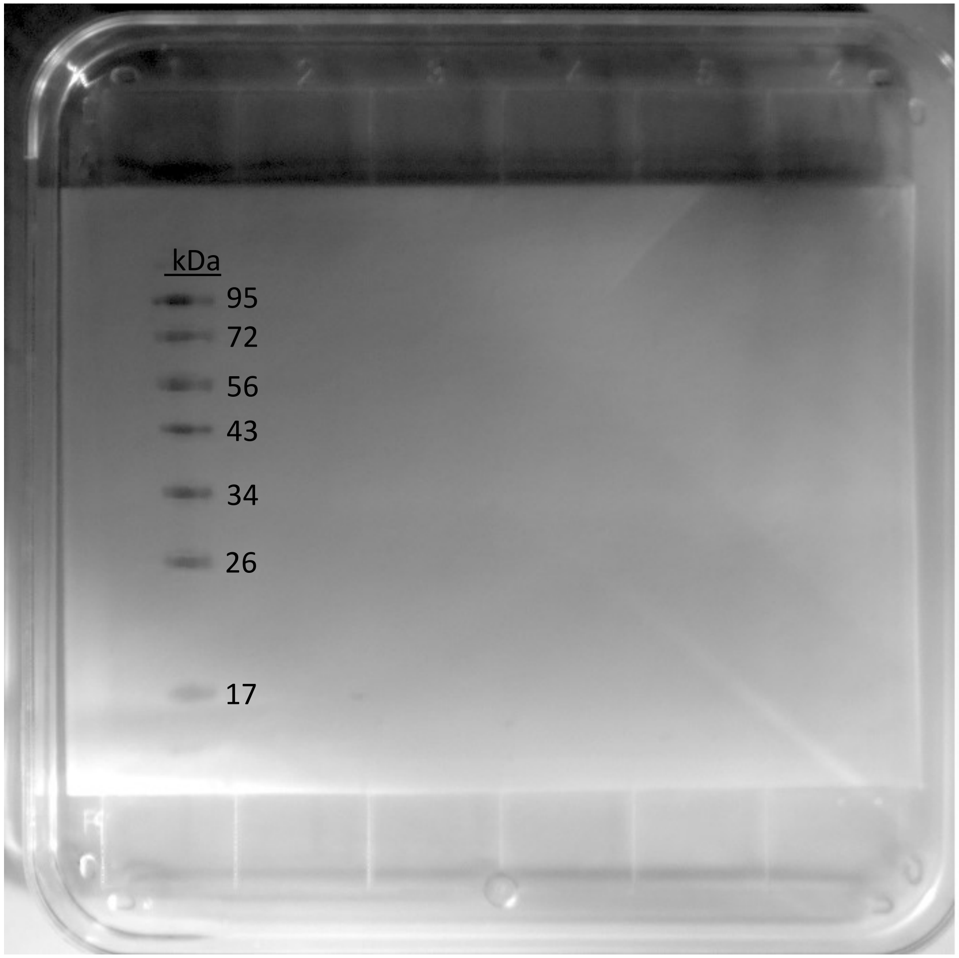

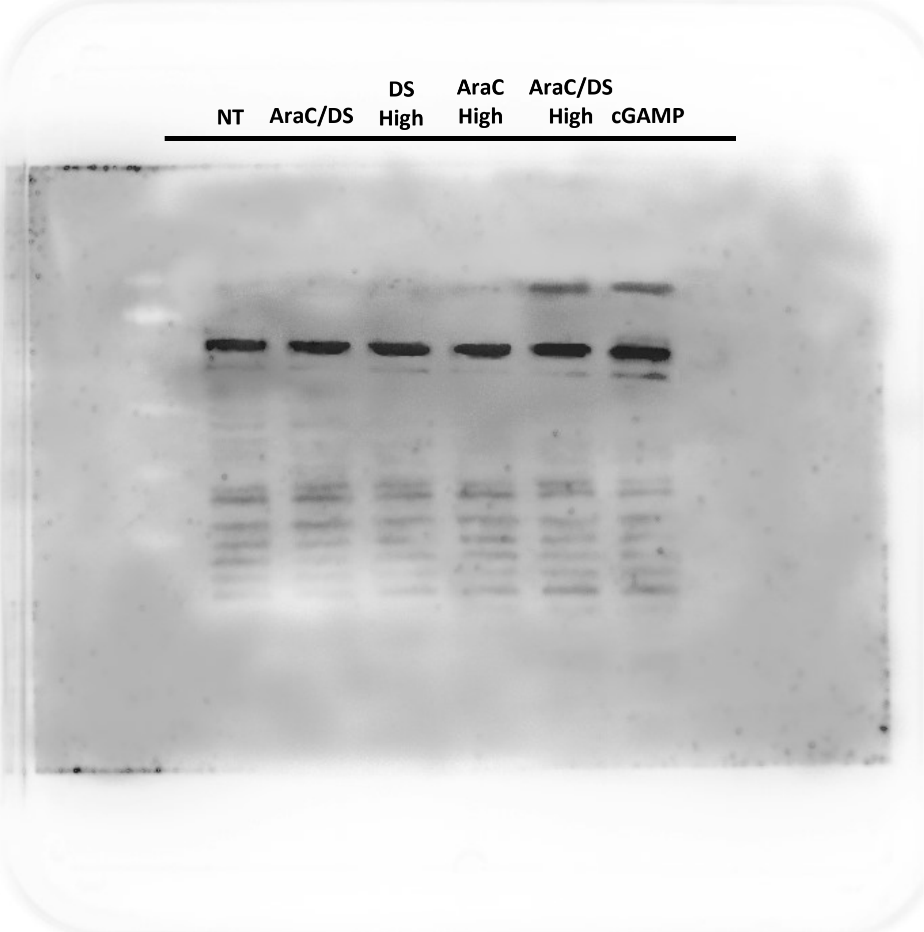

THP1-Macrophage, 6 hours, P-IRF3:

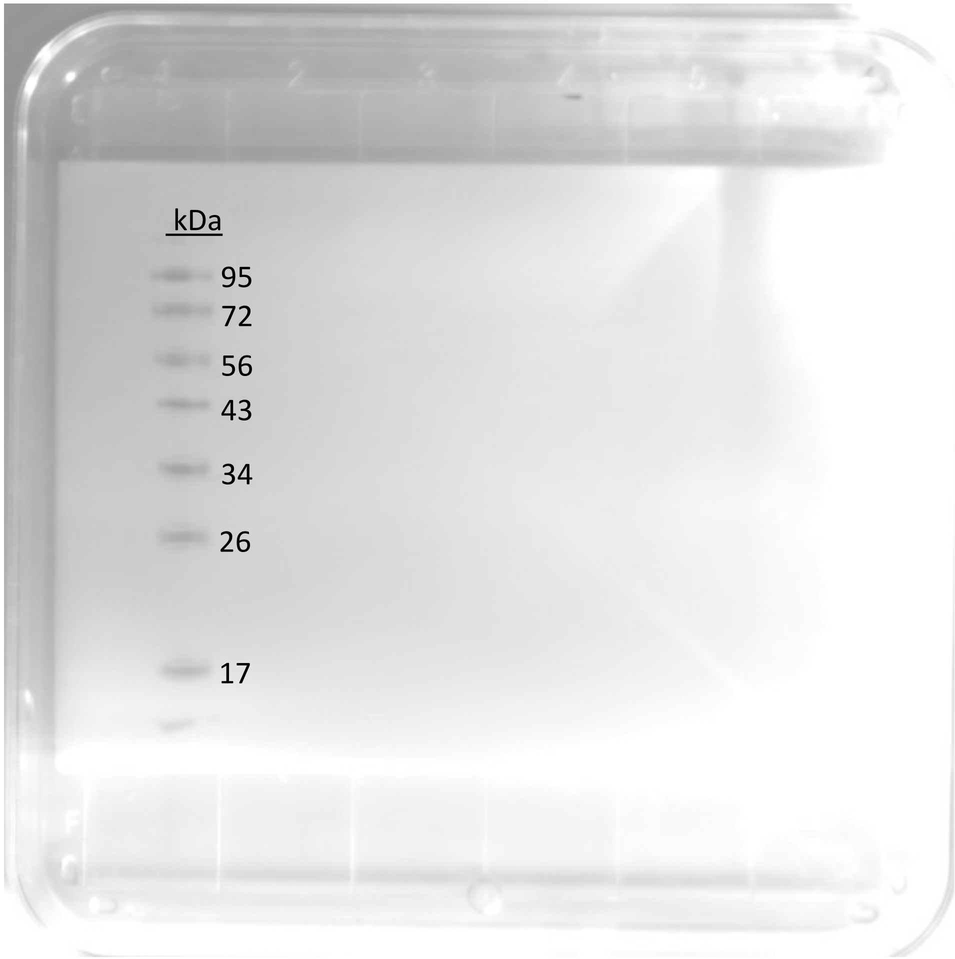

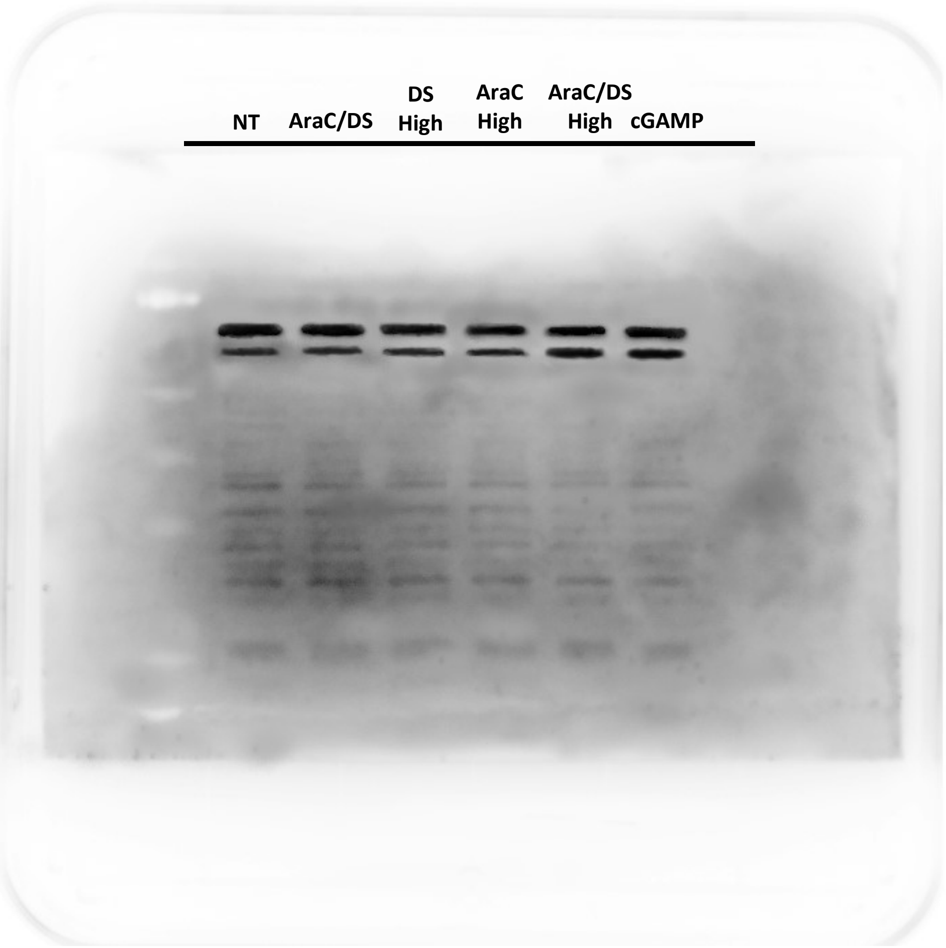

THP1-Macrophage, 6 hours, P-STING:

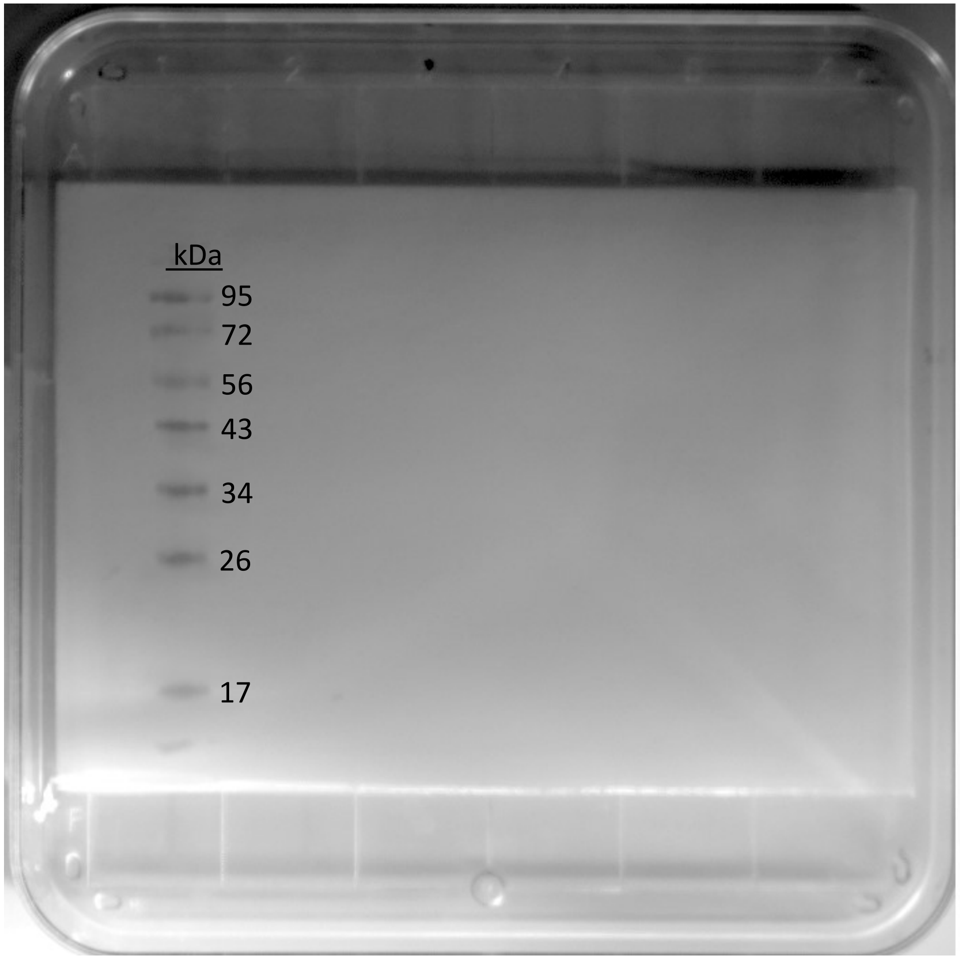

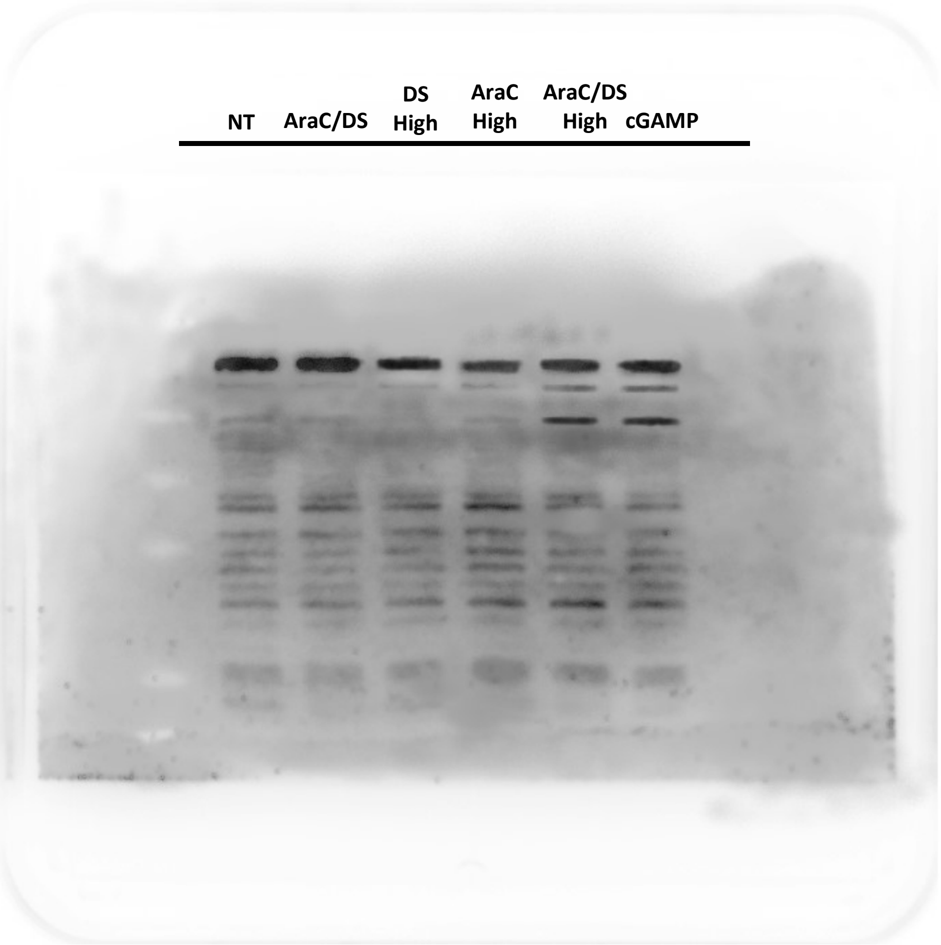

THP1-Macrophage, 6 hours, GAPDH:

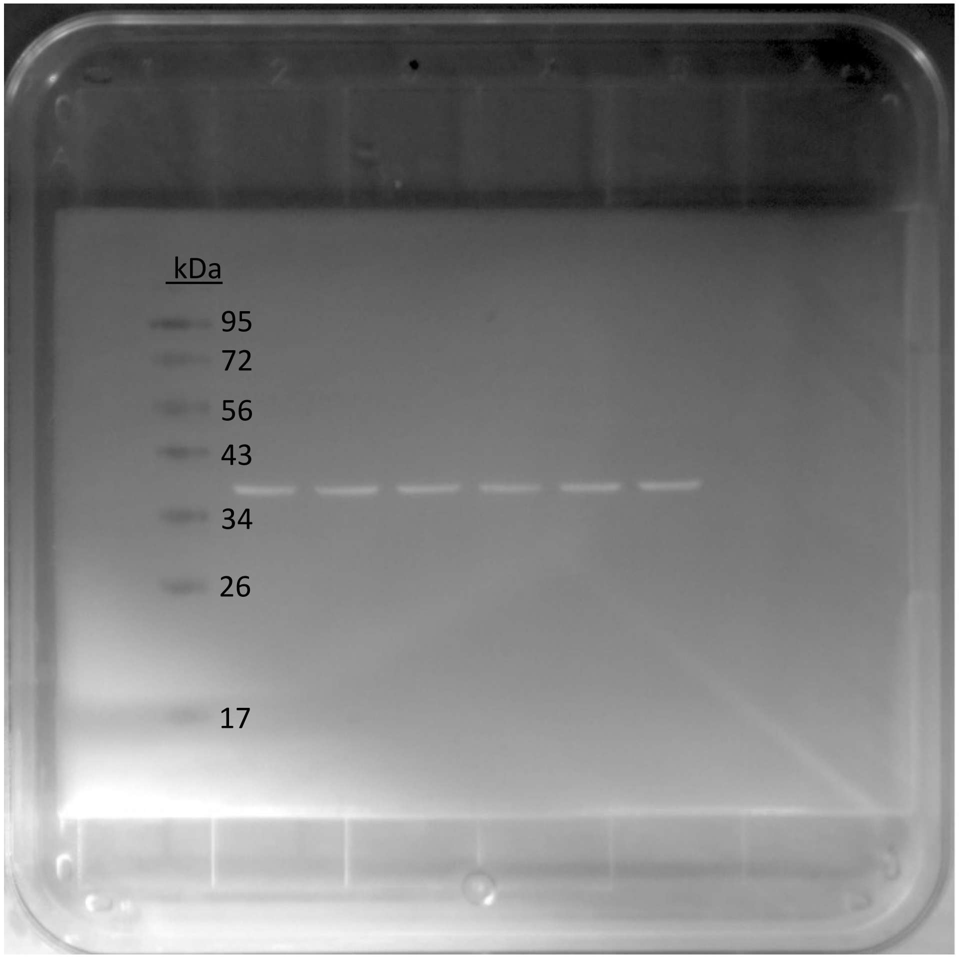

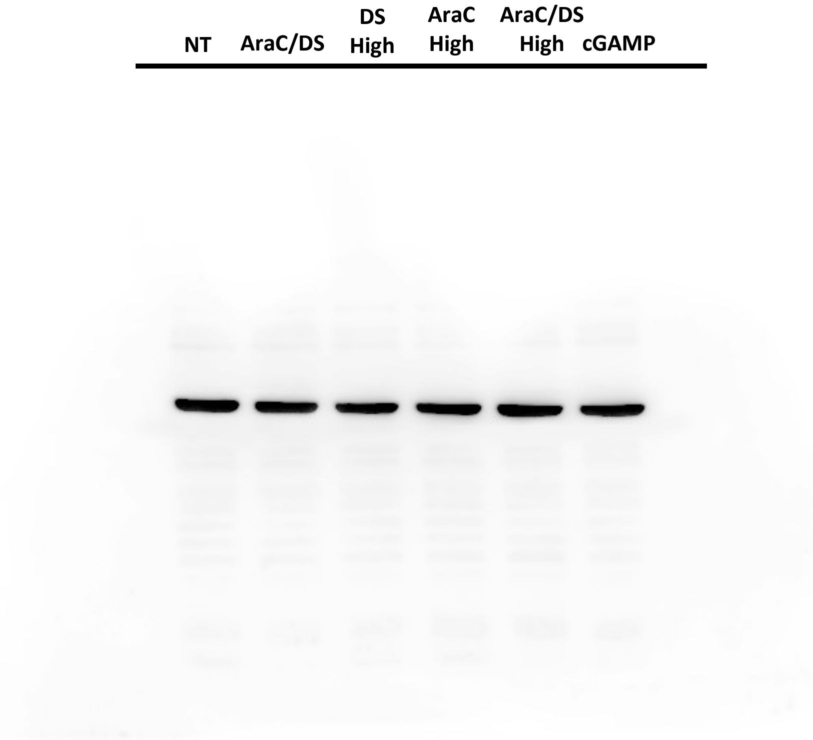

PBMC, 6 hours, P-TBK1:

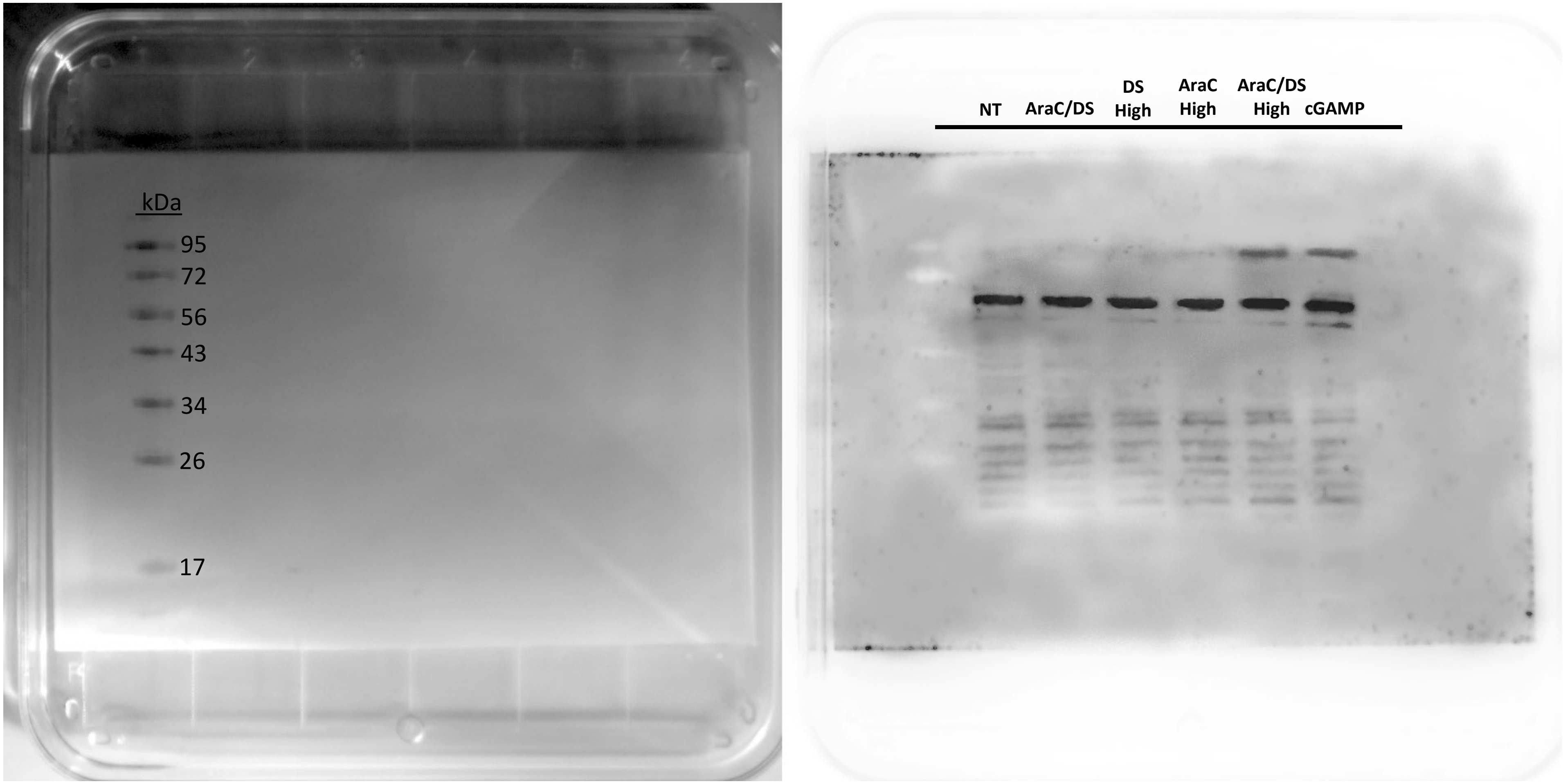

PBMC, 6 hours, P-IRF3:

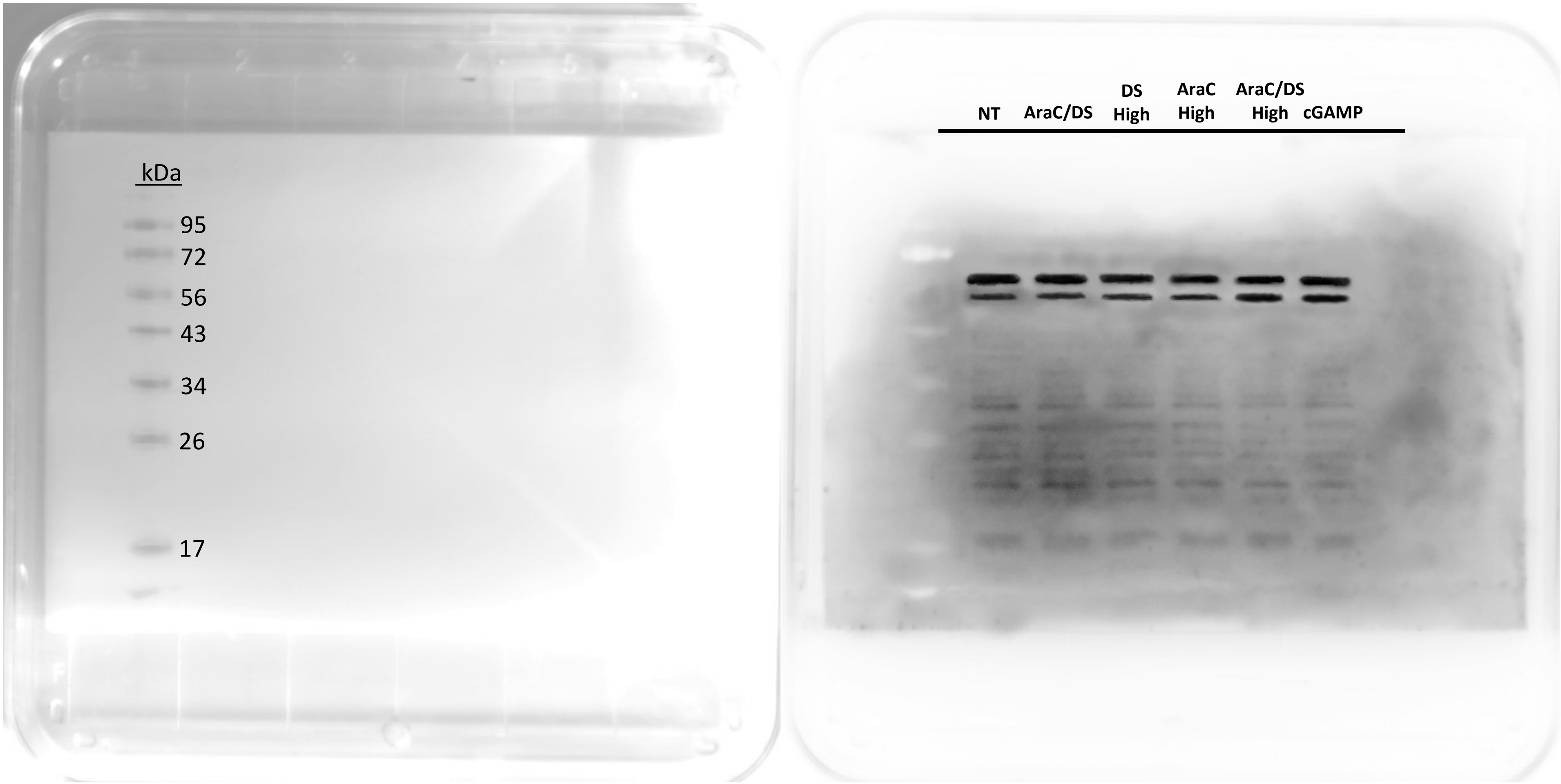

PBMC, 6 hours, P-STING:

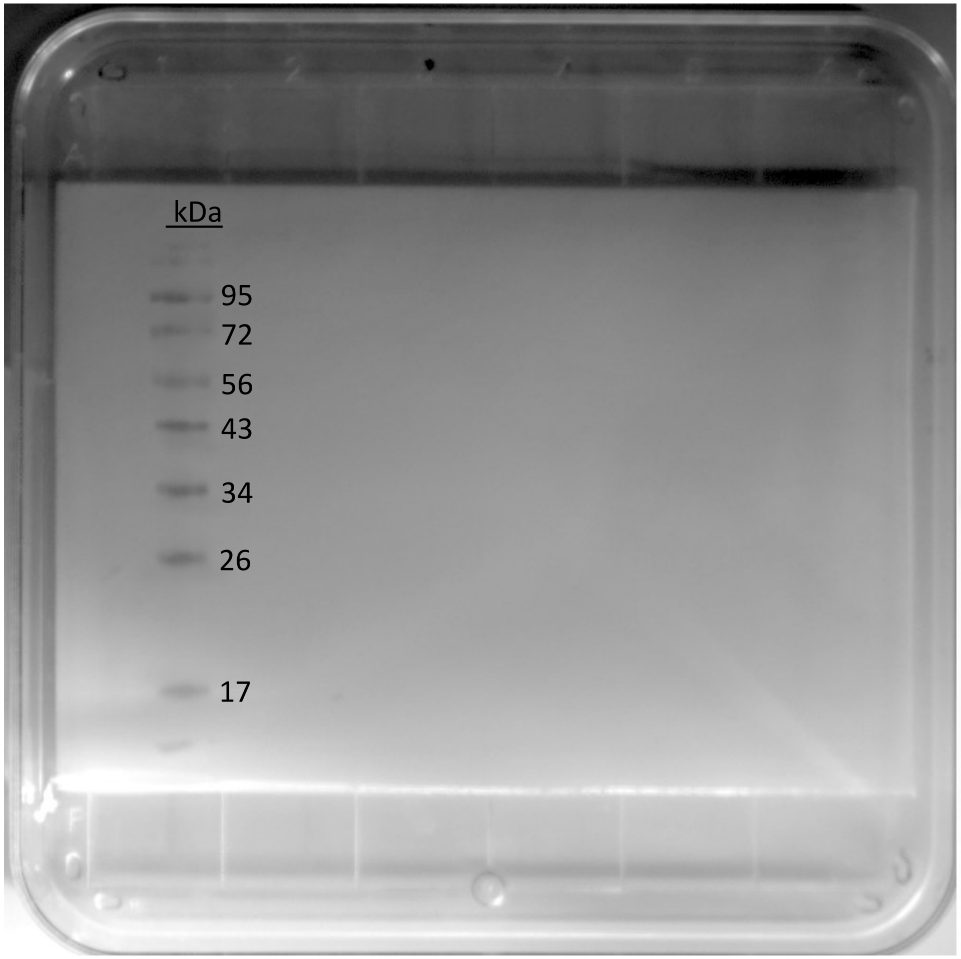

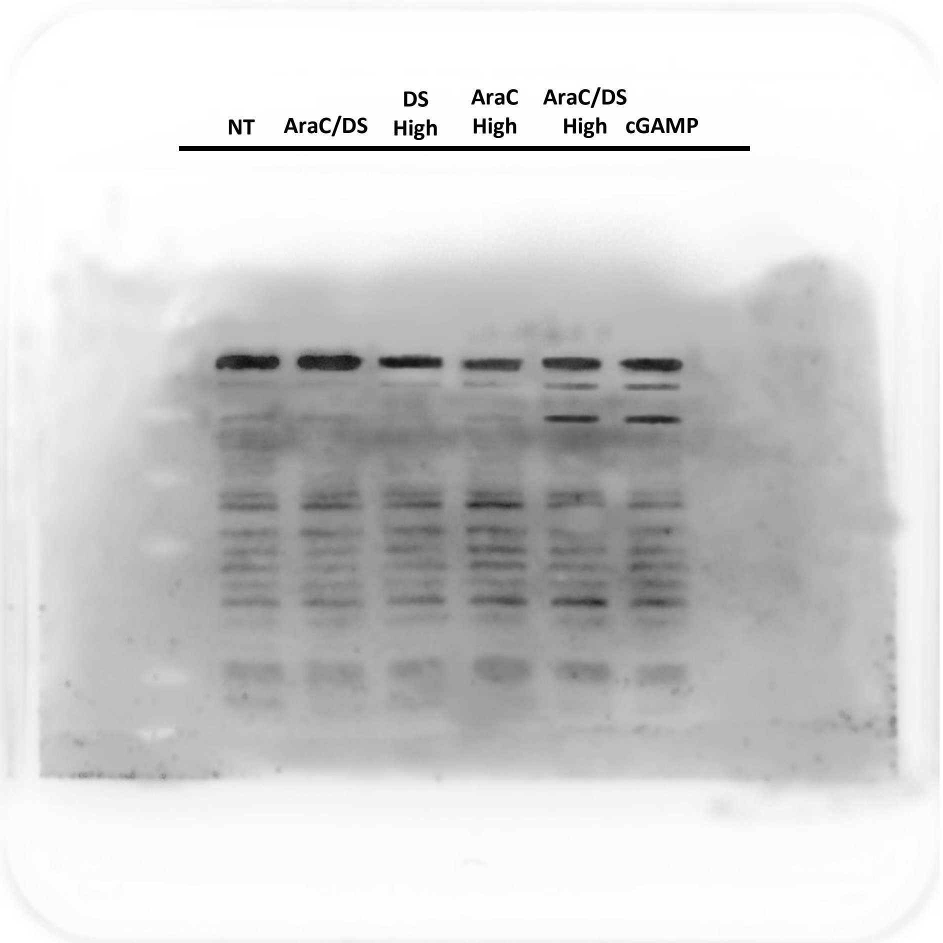

PBMC, 6 hours, GAPDH:

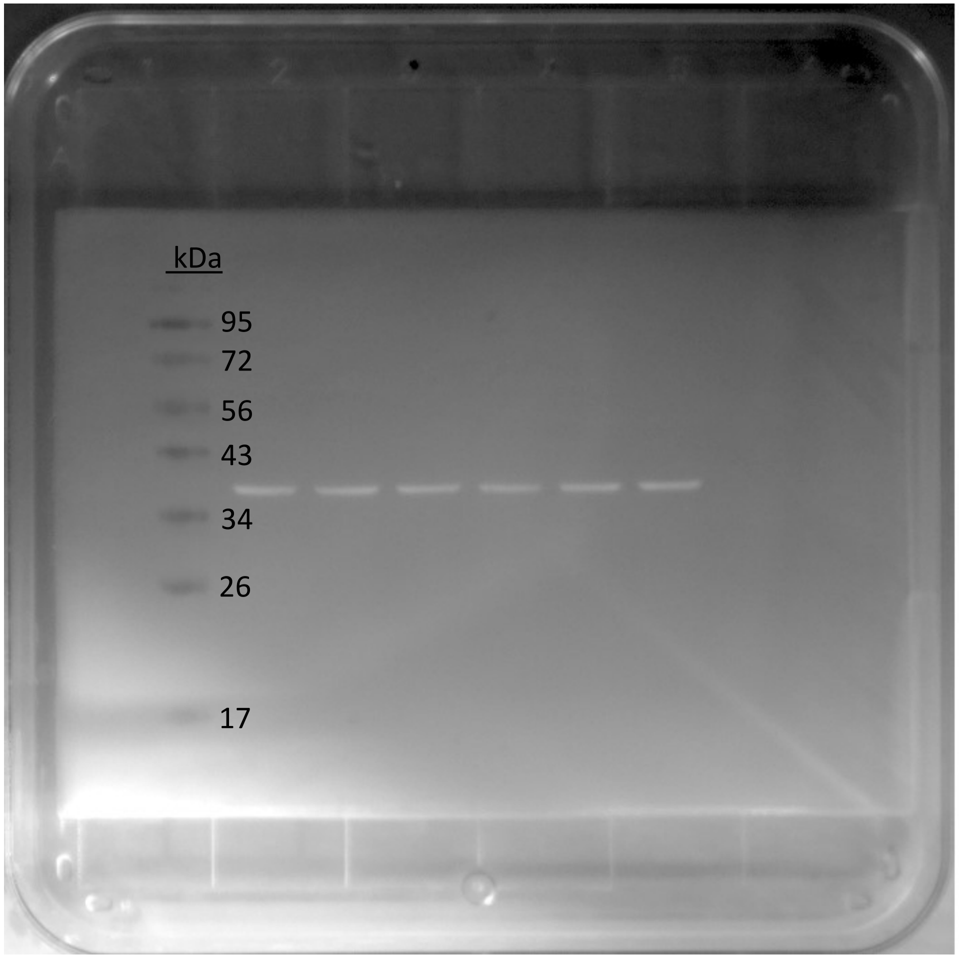

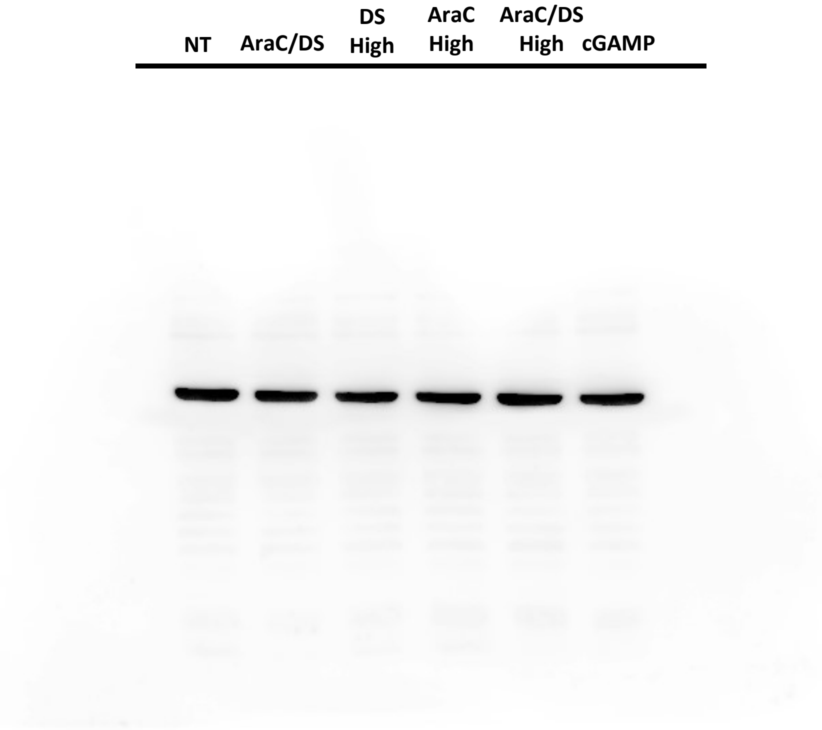

Supplement: Supplementary file 1 [file pharmaceutics-14-02710-s001.zip › pharmaceutics-2057840-supplementary.pdf]
